# A sequential MAP kinase cascade regulates mechanical signalling

**DOI:** 10.1038/s41467-026-74994-x

**Published:** 2026-07-01

**Authors:** Huy Cuong Tran, Essam Darwish, Viktor Johansson, Guadalupe Fernandez-Milmanda, Tingting Zhu, Cássio Flávio Fonseca de Lima, Brigitte Van De Cotte, Jean Colcombet, Marnik Vuylsteke, Ive De Smet, Alain Goossens, Olivier Van Aken

**Affiliations:** 1 Department of Biology, Lund University, Lund, Sweden; 2 Plant Physiology Section, Agricultural Botany Department, Faculty of Agriculture, Cairo University, Giza, Egypt; 3 Department of Plant Biotechnology and Bioinformatics, Ghent University, Ghent, Belgium; 4 VIB Center for Plant Systems Biology, Ghent, Belgium; 5 Université Paris-Saclay, CNRS, INRAE, Institute of Plant Sciences Paris-Saclay (IPS2), Gif sur Yvette, France

**Keywords:** Abiotic, Plant signalling, Kinases

## Abstract

Plants respond to mechanical stimulations like wind, touching or wounding to safeguard their development and survival. Mitogen Activated Protein Kinases (MAPKs) activation by mechanostimuli was reported 26 years ago, but the upstream regulatory mechanism and function remained unknown. We report that mechanostimulation activates a MAPKKK3/4/5-MKK4/5-MPK3/6 cascade within 60 seconds, leading to induction of ~800 genes, encompassing most of the early touch response. Most genes overlap with touch-responsive genes regulated by CAMTA1/2/3, exposing an interplay between MAPKs and CAMTA transcription factors. Furthermore, loss of MKK4/5 leads to global impairment of touch-regulated protein phosphorylation, demonstrating they are crucial regulators. In contrast, phosphorylation does not clearly affect early activation of the touch-induced jasmonic acid (JA) signalling pathway, nor does loss of JA affect the overall early touch-phosphoproteome. Lastly, loss of MAPKKK3/4/5 and MKK4/5 reduces thigmomorphogenesis, underlining the importance of the identified MAPK cascade for steering plant growth during stress. In summary, we have now identified a MAPKKK3/4/5-MKK4/5-MPK3/6 cascade as a key touch- and wounding signalling pathway in plants.

## Abstract (web summary)

Mechanical stimulation by touch triggers rapid and widespread changes in protein phosphorylation in plants. Here the authors identify a MAPKKK3/4/5-MKK4/5-MPK3/6 phosphorylation cascade as a key signaling pathway mediating plant responses to touch and wounding.

## Introduction

Plants frequently encounter adverse growth conditions such as drought, salinity, cold and high temperatures, pathogen and herbivore attacks. Plants are sessile, thus they have developed complex molecular responses to different environmental factors. Priming—the molecular memorization of past events—makes plants more battle-ready for future events^1,2^, helps them to enhance resistance to pathogens and to physiologically adapt to the physical environment^3^. Environmental factors that plants usually have to cope with include mechanical stimulations such as wind, rain, being touched/treaded on, being wounded by herbivores, or penetration of hyphae on the leaf surface. Mechanoperception is not only present in plants that have specialized sensory mechanism to respond to mechanical signals, e.g. carnivorous plants like Venus flytrap, sundew or *Mimosa pudica*^4^, but likely in every plant. Mechanical stimulations, such as touching, bending, brushing and sound vibration (mechanical waves), lead to rapid molecular responses in plants, e.g. calcium ion (Ca^2+^) spiking, enhanced reactive oxygen species (ROS) production, changes in gene expression and hormonal alteration^5–7^. Repetition of external mechanical stimulation on plants results in thigmomorphogenesis^8^, with substantial changes in plant morphology in severe cases, e.g. dwarfism, late bolting time, changes in mechanical properties of the stem, and a decrease in stomatal aperture^5^. However, experimental evidence suggests that repetitive mechanical stimulation can improve plant fitness and stress resistance. For example, periodic bending of Arabidopsis leaves increases tolerance against the necrotrophic fungus *Botrytis cinerea* and the herbivore *Trichoplusia ni*^9^, while sound vibration treatment on Arabidopsis improves tolerance against *Botrytis cinerea* and drought stress^10,11^. Mechanostimulation is also a good technique to control plant lodging by impacting the reinforcement of the aerial parts and the root anchoring^12–14^. Altogether, these suggest that thigmo-priming (“thigmo” means “I touch” in Greek) – the defense priming obtained by repetitive mechanostimulation, is a promising solution to increase agricultural productivity.

To date, several molecular players have been identified in plant mechanoperception. A major group of potential mechanoreceptors is mechanosensitive-ion (MS) channels – the transmembrane proteins that perceive membrane tension and translate the mechanical force into cellular ion flux. MS channels are divided into three groups: MS channel of small conductance-like (MSL) that prefers anion influx, Mid1-complementing activity family (MCA) and REDUCED HYPEROSMOLALITY-INDUCED CA^2+^ INCREASE (OSCA) that are Ca^2+^-permeable channels^15,16^. Another type of mechanoreceptors is plasma-membrane-localized receptor-like kinases (RLKs) that respond to cell wall damage (CWD) and/or cell wall integrity (CWI) signalling caused by mechanostimulation^17^. Of more than 600 RLKs in Arabidopsis, only FERONIA, a member of the *Catharanthus roseus* RLK1-like subfamily, is known to be involved in plant mechanical signal transduction^17,18^. Knockout of *feronia* in Arabidopsis led to compromised hypoosmotic stress signalling, a decrease in mechanoresponsive gene expression, and changes in root growth responses to mechanically challenging environments^18^. Darwish et al.^19^ showed that FERONIA represses at least part of the JA-dependent response to brushing, rather than triggering touch response signalling.

Recent studies have shed further light on the molecular mechanisms underlying how plants respond to mechanical stimulation. Interestingly, Arabidopsis touch- and wind-stimulated *mslΔ5* (*msl4;msl5;msl6;msl9;msl10*) knockout mutants showed no different phenotype compared to the wildtype, indicating that the molecular response to mechanical stimulation is more complicated than cellular mechanosensing alone^3,20–23^. A recent study showed that MSLs may be involved in cell-to-cell spreading of calcium signals after mechanical stimulation, rather than acting in local touch perception^24^. Wang et al.^25^ showed that the phosphorylation of the MAPK kinase (MKK) 1 and 2, and TOUCH-REGULATED PHOSPHOPROTEIN 1 (TREPH1) occurs rapidly after touching, and TREPH1 is required for touch-induced flowering delay. Arabidopsis jasmonic acid (JA)-deficient mutants *aos* and JA-signalling-deficient mutant *myc2 myc3 myc4* exhibit impaired thigmomorphogenesis, indicating that JA biosynthesis and signalling is important for mechanoresponse in plants^9,20^. The JA-activated transcription factors MYC2/MYC3/MYC4 are positive regulators of mechanoresponsive genes like *JASMONATE-ZIM-DOMAIN PROTEIN 8* (*JAZ8*), *ETHYLENE RESPONSE FACTOR 109* (*ERF109*) and *CALMODULIN LIKE 39* (*CML39*), which are specific marker genes in the JA-dependent pathway. Recent studies by Matsumura et al.^26^ and Darwish et al.^19^ show that the CALMODULIN-BINDING TRANSCRIPTIONAL ACTIVATOR 3 (CAMTA3) is a regulator of touch response signalling. In addition, CAMTA3 and its homologs CAMTA1 and CAMTA2 redundantly regulate touch responsive genes, e.g. *TOUCH2* (*TCH2*), *TCH4*, *CYSTEINE-RICH RECEPTOR-LIKE PROTEIN KINASE 41* (*CRK41*) and *PHLOEM PROTEIN 2A5* (*PP2A5*), in a JA-independent manner^19^. Besides JA, other hormonal players have been involved in thigmomorphogenesis. Gently rubbing the internodes of bean plants between fingers causes the release of ethylene (ET)^27^. In Arabidopsis, repetitive touching of mutant lines defective in ET biosynthesis and signalling causes a strong repression of rosette growth and delay in flowering, indicating that the ET pathway is a repressor of thigmomorphogenesis^28^. Interestingly, JA and ET pathways control thigmomorphogenesis by regulating the expression of genes coding for gibberellin (GA) catabolic enzymes in an opposite manner, i.e. JA enhances GA breakdown while ET represses it^28,29^. As GAs are growth- and flowering-promoting hormones, their inactivation may be important to trigger thigmomorphogenesis^21^.

Mechanostimulation rapidly activates plant immune responses. Twenty-six years ago, Ichimura et al.^30^ showed that the clade-A MAPKs like MPK6 are rapidly activated by various abiotic stresses, including mechanical stresses (touch and wounding). Specifically, MITOGEN-ACTIVATED PROTEIN KINASES (MAPKs) MPK3 and MPK6 phosphorylation was observed in Arabidopsis after rain- and brushing-stimulation, suggesting that MAPK cascades are of importance for mechanotransduction^26,30^. However, the upstream components of the MPK3/6 phosphorylation cascade upon touch have not been established. Here, we show that MAPK kinases, MKK4 and MKK5, are regulators of touch response. Arabidopsis loss-of-function *mkk4 mkk5* mutants exhibit a wide range of alterations in transcriptional response to touch treatment. We observed that the touch-induced transcript profile of *mkk4/5* mutants shares similarity with that of *camta1/2/3* mutants, suggesting that MKK4/5 partially cooperate with CAMTA1/2/3 in mechanotransduction. Using a phosphoproteomic approach, we showed that loss of *mkk4/5* leads to dramatic alterations in protein phosphorylation patterns in response to touch. Conversely, this response was generally similar between wild type (WT) and JA-deficient *aos* lines, suggesting that the phosphorylation cascade triggered in response to touch does not depend strongly on JA production. By mining the phosphoproteomic data, we identified MAP kinase kinase kinases (MAPKKK) 3, 4 (YODA), and 5 as the upstream activators of the MKK4/5-MPK3/6 cascade in plant touch response. Finally, we show that *mapkkk3/4/5* and *mkk4/5* mutant show a relatively smaller reduction in rosette size after touching, indicating that the MAPKKK3/4/5-MKK4/5-MPK3/6 cascade plays a role in thigmomorphogenesis.

## Results

### MKK4 and MKK5 are involved in touch-induced gene expression

**Fig. 1:**
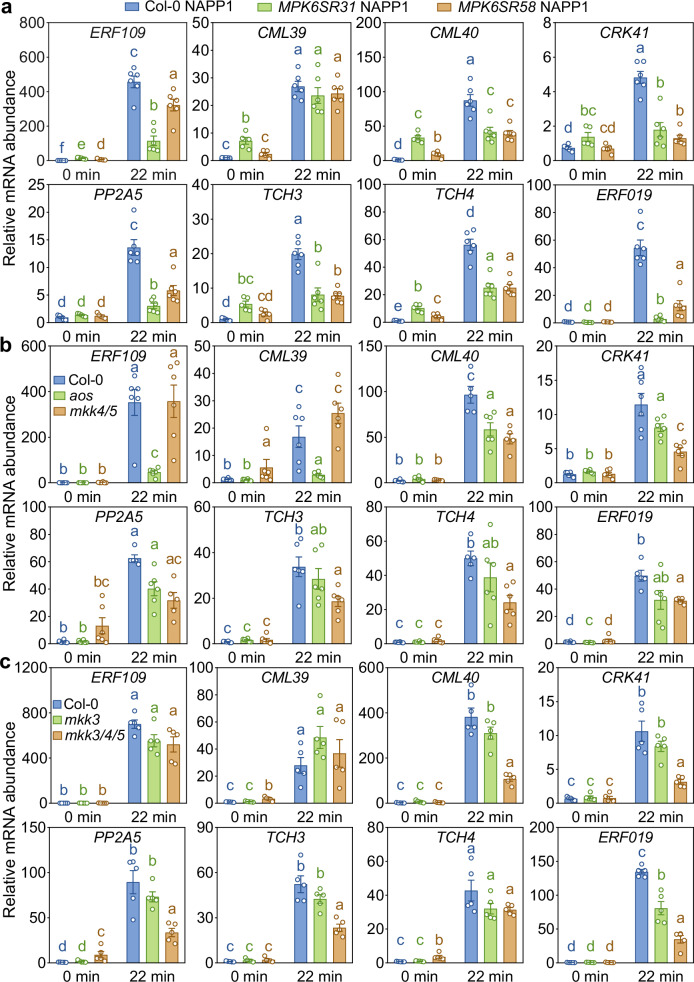
Analysis of touch marker gene expression in Arabidopsis mutants. 12-day-old seedlings before (0 min) and 22 min after touching by gentle brushing were collected for qRT-PCR. The expression of selected touch marker genes was measured in Col-0 (WT) treated with NAPP1 versus *MPK6SR31* and *MPK6SR58* treated with NAPP1 (**a**), WT versus *aos* and *mkk4/5* (**b**), WT versus *mkk3* and *mkk3/4/5* (**c**). The y axis represents the relative mRNA level. Data are presented as mean ± SE. *n* = 6 (**a**), *n* = 6 (**b**) and *n* = 5 (**c**) biologically independent samples. Each dot indicates a biological replicate. Statistical significance was based on Kruskal-Wallis test followed by Wilcoxon rank sum tests. Different letters represent the significant differences between genotypes (*p* < 0.05).

Raindrops and mechanical stimuli (brushing) on Arabidopsis rosette leaves lead to the rapid phosphorylation of MPK3 and MPK6 (within 1 min)^26^. Therefore, we performed quantitative real-time-polymerase chain reaction (qRT-PCR) on 12-day-old Arabidopsis *mpk6-3* single mutant to check if MPK6 plays a role in touch response at transcript level. We only observed a significantly lower induction of *TCH3* after touching, and there was no significant effect on JA-dependent (*ERF109*, *CML39, JAZ8* and *CML40*) and other JA-independent (*CRK41*, *PP2A5*, *TCH2*, *TCH4*, *ERF019* and *CML38*) touch genes in the *mpk6-3* mutant (Supplementary Fig. 1). As MPK3 and MPK6 function redundantly and the *mpk3 mpk6* double mutant is embryo lethal, we examined touch response in the conditional loss-of-function mutants *mpk3 mpk6 pMPK6:MPK6*^*YG*^ (*MPK6SR31* and *MPK6SR58*)^31^. MPK6^YG^ rescues the embryo lethality of *mpk3 mpk6* double mutants but is sensitive to inhibition by 4-amino-1-tert-butyl-3-(10-naphthyl)pyrazolo[3,4-d] pyrimidine (NAPP1)^31,32^. Therefore, we carried out qRT-PCR on the *MPK6SR* lines grown on half-strength MS plates containing 1 µM NAPP1 to analyze the expression of touch marker genes 22 min after gentle brushing (Fig. 1a and Supplementary Fig. 2a). We observed that the expression of *CML38*, which was suggested to be touch-regulated by TREPH1, and of the JA-responsive gene *JAZ8* was not affected by loss of MPK3 and MPK6 (Fig. 1a and Supplementary Fig. 2b). In contrast, other JA-responsive genes, *ERF109* and *CML40*, were less induced in *MPK6SR31* and *MPK6SR58* treated with NAPP1. This indicates that there might be a partial cross talk between JA and MPK3/6-related touch signalling pathways. CAMTA1/2/3-regulated (JA-independent) genes, including *CRK41*, *PP2A5* and *TCH4*, showed a significant decrease in gene expression in the *MPK6SR31* and *MPK6SR58* treated with NAPP1 (Fig. 1a and Supplementary Fig. 2b). Interestingly, *TCH3* and *ERF019*, whose touch response regulators are unknown, were less induced in NAPP1-treated *MPK6SR31* and *MPK6SR58* (Fig. 1a and Supplementary Fig. 2b). Together, these results indicate that MPK3 and MPK6 are touch regulators and might cooperate with CAMTA1/2/3 to regulate plant touch response.

In plants, the activation of clade-A MAPKs MPK3 and MPK6 by wounding relies on the upstream MAPK kinases MKK4 and MKK5, and this effect is JA-independent^33^. Previous studies showed a clear overlap between the wound and touch response, e.g. a rapid accumulation of calcium^34^ and induction of genes that are involved in both wound and touch response, including *JAZ10*, *LYPOXYGENASE 3* (*LOX3*), *ALLENE OXIDE CYCLASE* (*AOC*), *CML39*, *TCH2* and *TCH4*^9,35–37^. Thus, we hypothesized that a MKK4/5-MPK3/6 cascade could be involved in touch response in plants. Therefore, we carried out qRT-PCR to check the touch-induced genes in *mkk4/5* mutants^38^, and included the JA-deficient *aos* mutant^39^ as a control for JA-dependent signalling (Fig. 1b and Supplementary Fig. 3a). JA-dependent touch marker genes, including *ERF109*, *CML39*, *JAZ8* and *CML40* were significantly less induced by touching in the *aos* mutant compared to in WT. This is in agreement with the previous study by Van Moerkercke et al.^20^, in which these JA-genes were downregulated in the *myc2/3/4* mutant after touching. However, these JA-dependent genes, except *CML40*, were unchanged in the *mkk4/5* mutant. CAMTA1/2/3-regulated (JA-independent) genes, including *CRK41*, *PP2A5* (*p*-value = 0.052) and *TCH4*, and *TCH3* and *ERF019*, which are regulated by MPK3/6 (Fig. 1a and Supplementary Fig. 2b), showed a significant decrease in touch-induced expression in the *mkk4/5* mutant. *CML38* and *TCH2*—another CAMTA1/2/3-regulated gene—responded similarly to Col-0 in the *mkk4/5* mutant. Together, these findings suggest that MKK4 and MKK5 might directly or indirectly cooperate with CAMTA1/2/3, possibly via phosphorylation of MPK3 and MPK6, to induce touch-responsive genes in a JA-independent pathway.

In plants, the MKK3-MPK1/2/7 cascade is activated mainly through wound-induced JA production and this module is independent of the MKK4/5-MPK3/6 cascade in wound response^33^. Therefore, we examined the expression of JA-dependent and CAMTA1/2/3-dependent touch-induced genes in the *mkk3* and newly-generated *mkk3/4/5* triple mutants (Fig. 1c and Supplementary Fig. 3b). JA-dependent genes (*ERF109*, *JAZ8*, *CML39* and *CML40*), CAMTA1/2/3-induced genes (*CRK41*, *PP2A5*, *TCH2* and *TCH4*), and MKK4/5-MPK3/6-induced genes (*TCH3* and *ERF019*) showed no changes in the *mkk3* mutant. In the *mkk3/4/5* mutant, JA genes, except *CML40*, were largely unchanged, whereas CAMTA1/2/3- and MKK4/5-MPK3/6- dependent genes were significantly downregulated, which is in agreement with our qPCR results for *mkk4/5* (Fig. 1b, c and Supplementary Fig. 3). These results suggest that MKK3 may not have a major role in touch signalling.

In conclusion, we found that the MKK4/5-MPK3/6 signalling cascade plays a role in touch-induced gene expression, potentially in cooperation with CAMTA1/2/3.

### Loss of *CAMTA1/2/3* and *MKK4/5* results in widespread changes in the touch-induced transcriptome

Next, we performed RNA-sequencing (RNA-seq) analysis to examine the global transcript profile of the *mkk4/5* mutant in response to touch. We found that several CAMTA1/2/3-regulated genes were significantly downregulated in the *mkk4/5* mutant (Fig. 1b, c). Therefore, we also performed RNA-seq analysis in the *camta1/2/3* mutant not only to gain a deeper insight into the roles of CAMTA1/2/3 simultaneously in touch response, but also to compare the transcript profiles of touch-induced response between the *mkk4/5* and *camta1/2/3* knockouts. As we observed that touch-induced gene expression peaks after 22 min for several marker genes (Supplementary Fig. 4), which is consistent with Van Moerkercke et al.^20^ and Darwish et al.^19^, we performed RNA-seq analysis using seedlings before (0 min) and 22 min after touching by gentle brushing.

In this RNA-seq analysis, we found that 2032 genes significantly responded to touch after 22 min (Supplementary Data 1). Comparison of the 2032 touch responsive genes found in our RNA-seq dataset and the 1671 “core touch-responsive” genes defined previously^20^ showed that 507 of 1671 “core touch” genes were not touch-induced in our RNA-seq analysis. Therefore, we refined the list of core touch responsive genes. We observed that there were 1168 genes consistently responding to touch or water spray after 22–25 min using RNA-seq datasets from Van Moerkercke et al.^20^, Darwish et al.^19^ and this study (Supplementary Data 2). We thus defined these 1168 touch genes as “core early touch response” genes.

**Fig. 2:**
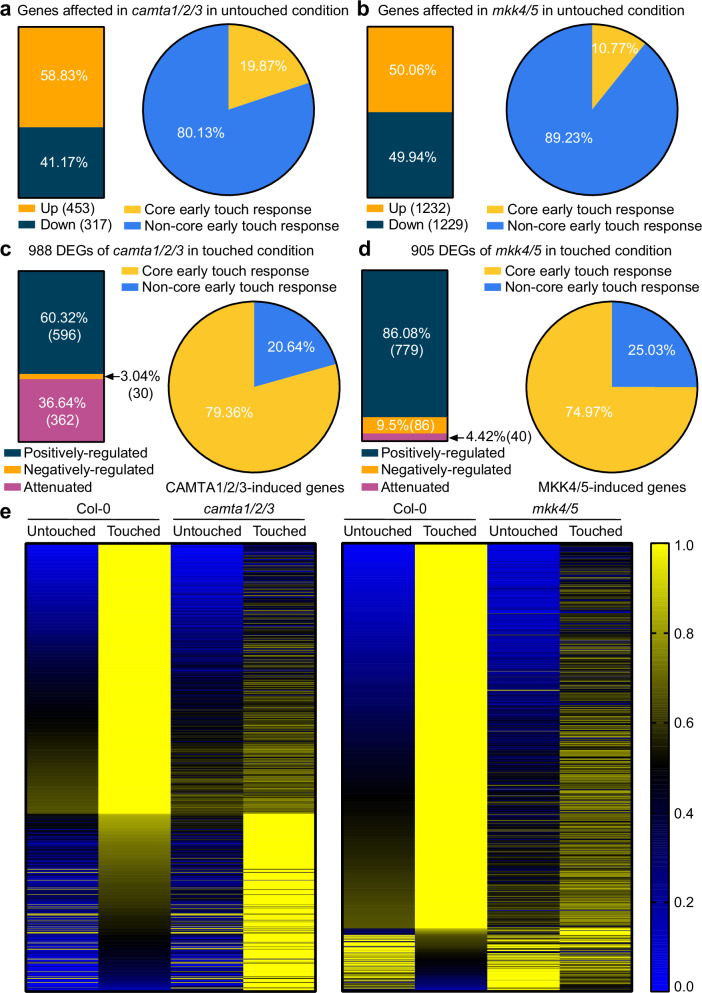
Dynamic transcript profiling of touch-induced response in the *camta1/2/3* and *mkk4/5* mutants. **a** Genes affected in the *camta1/2/3* mutant under untouched condition. Of all DEGs in *camta1/2/3* in untouched condition, ~20% belong to the “core early touch response” genes. **b** Genes affected in the *mkk4/5* mutant under untouched condition. Of all DEGs in *mkk4/5* in untouched condition, ~11% belong to the “core early touch response” genes. **c** Touch-responsive DEGs in the *camta1/2/3* mutant at 22 min versus WT at 22 min. Of 596 CAMTA1/2/3-touched induced genes, ~79% belong to the “core early touch response” genes. **d** Touch-responsive DEGs in the *mkk4/5* mutant at 22 min versus WT at 22 min. Of 779 MKK4/5-touched induced genes, ~75% belong to the “core early touch response” genes. **e** Heatmaps representing transcripts differentially expressed between Col-0 and the mutants (*camta1/2/3* and *mkk4/5*) under untouched and touched conditions. Minimum and maximum expression values were normalized between 0 and 1 for each gene.

Under untouched conditions, we observed that there were 770 and 2461 differentially expressed genes (DEGs) in the *camta1/2/3* and *mkk4/5* mutants compared to WT, respectively (Fig. 2a, b and Supplementary Data 3). Of these 770 DEGs in the *camta1/2/3* mutant, 453 genes (~59%) were upregulated and 317 genes (~41%) were downregulated, including *CAMTA1/2/3* themselves (Fig. 2a and Supplementary Data 3a). Half of the 2,461 DEGs in the *mkk4/5* mutant were up- or down-regulated (1232 (~50%) and 1229 (~50%) genes, respectively), including a decrease in *MKK5* transcript (Fig. 2a and Supplementary Data 3b). Approximately 20% and 11% of the DEGs affected by knocking out *CAMTA1/2/3* and *MKK4/5* were “core early touch response” genes (Fig. 2a, b and Supplementary Data 2, 3). This is substantially higher than expected by chance (5%), showing that MKK4/5 and CAMTA1/2/3 play a role in the regulation of some touch-induced genes even under untreated conditions. Nevertheless, the large majority of DEGs in untreated conditions appear to be involved in other processes rather than in touch response.

Next, we searched the 2032 touch-responsive genes in WT for genes that differentially responded to touch in the mutants. There were 988 and 905 DEGs in the *camta1/2/3* and *mkk4/5* mutants with altered touch response compared to WT, respectively (Fig. 2c, d and Supplementary Data 4). Of these 988 DEGs in the *camta1/2/3* mutant, 596 (~60%) and 30 (~3%) genes were positively- or negatively regulated by CAMTA1/2/3, respectively (Fig. 2c and Supplementary Data 4a). A relatively large group of 362 genes (~37%) was attenuated by CAMTA1/2/3, indicated by either exaggerated upregulation or downregulation in *camta1/2/3* compared to in WT (Fig. 2c and Supplementary Data 4a). The expression pattern was more straightforward in the *mkk4/5* mutant as compared to the *camta1/2/3* mutant, as the vast majority of DEGs (779 genes, ~86%) required MKK4/5 for their full induction, and 86 (~10%) genes required MKK4/5 for their full downregulation (Fig. 2d and Supplementary Data 4b). Only 4% (40 genes) were attenuated by MKK4/5, meaning they were more induced or more downregulated after touch in *mkk4/5* as compared to the WT (Fig. 2d and Supplementary Data 4b). Most of the DEGs (>70%) that were positively regulated by CAMTA1/2/3 and MKK4/5 were considered as “core early touch response” genes (Fig. 2c, d and Supplementary Data 2, 4). This indicates that MKK4 and MKK5 are largely positive regulators of touch response in plants. In line with the previous study^19^, we showed that CAMTA1/2/3 are also mostly positive regulators of touch response in plants, though for many genes a more complex “attenuation” role for CAMTA1/2/3 was observed. As visualized by the heatmaps, MKK4/5 indeed seems to have a more uniformly positive effect in touch response compared to CAMTA1/2/3 (Fig. 2e and Supplementary Data 5).

### MKK4/5 may cooperate with CAMTA1/2/3 in touch-induced transcriptional response

**Fig. 3:**
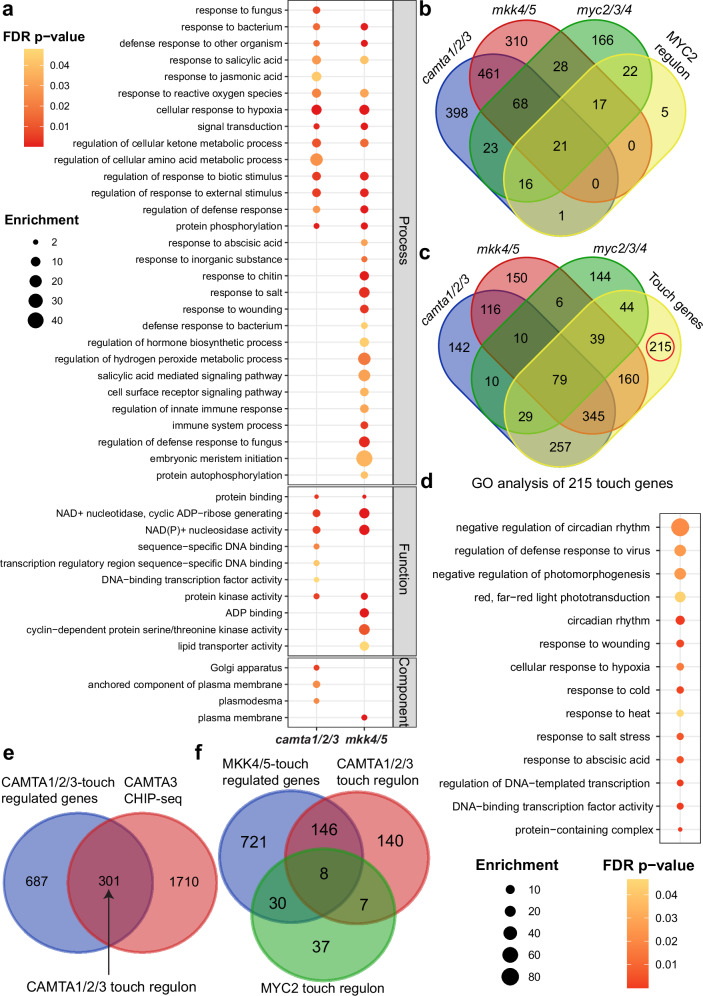
Comparison of transcription profiles of *camta1/2/3*, *mkk4/5*, and *myc2/3/4* mutants. **a** Dot plot represents comparison of GO analyses of CAMTA1/2/3- and MKK4/5-induced genes after touching. Color bar indicates *p*-values after False Discovery Rate (FDR) correction. **b** Venn diagram shows comparison of transcript profiles of *camta1/2/3*, *mkk4/5*, *myc2/3/4* and the MYC2 touch regulon^20^. **c** Venn diagram shows comparison of transcript profiles of *camta1/2/3*, *mkk4/5*, *myc2/3/4* and “core early touch responsive” genes. **d** Dot plot represents GO analysis of 215 “core early touch response” genes that are not regulated by either CAMTA1/2/3, MKK4/5 or MYC2/3/4. Color bar indicates *p*-values after FDR correction. **e** CAMTA1/2/3 touch regulon obtained by comparison of CAMTA1/2/3-induced touch genes and CAMTA3 CHIP-seq analysis^26^. **f** Comparison of MKK4/5-induced touch genes versus CAMTA1/2/3- and MYC2- touch regulon^20^.

Using qRT-PCR, we had observed similar changes in the expression of JA-dependent/independent touch responsive genes in the *mkk4/5* and *camta1/2/3* mutants (Fig. 1b, c). This suggests that *mkk4/5* shares similarity in transcriptional changes with *camta1/2/3* in touch-induced response. To further support this, we compared the RNA-seq datasets of the *mkk4/5* and *camta1/2/3* mutants (Fig. 3). We first used the touch-induced genes by CAMTA1/2/3 (596) and MKK4/5 (779) found in the RNA-seq datasets for gene ontology (GO) analysis (Supplementary Data 6). Comparison of GO analyses (enriched GO terms with p*adj* < 0.05) showed that *camta1/2/3* and *mkk4/5* mutants shared many GO categories involved in response to fungus, bacterium, salicylic acid (SA), reactive oxygen species and hypoxia, signal transduction, etc. (Fig. 3 and Supplementary Data 6). Nevertheless, *mkk4/5* and *camta1/2/3* had specific sets of genes involved in different processes or different cellular functions (Fig. 3a and Supplementary Data 6). *mkk4/5* specifically had genes involved in response to abscisic acid (ABA), inorganic substances, salt and wounding, regulation of innate immune response, embryonic meristem initiation, protein autophosphorylation, etc. *camta1/2/3* specifically had genes involved in response to JA, regulation of cellular amino acid metabolic process, transcription regulatory region sequence-specific DNA binding, Golgi apparatus, anchored component of plasma membrane, and plasmodesma. The enrichment of JA-responsive genes was interesting, as CAMTA1/2/3 largely regulates JA-independent genes. This may likely be explained by a smaller set of genes that seem to be targeted by both CAMTA1/2/3 and JA pathways, as previously reported for example *bHLH19*^19^ (see also Fig. 3f). *bHLH19* was also observed to be about 4.5-fold less induced after touch in *camta1/2/3* as compared to in Col-0, though the p just fell outside our cut-off (*p* = 0.057), likely due to the very low read counts for this gene resulting in more noise (Supplementary Data 4). Overall, knocking out *CAMTA1/2/3* and *MKK4/5* has partially overlapping effects on genes mostly involved in defense response to biotic and abiotic stresses, and protein phosphorylation after touching. However, *mkk4/5* and *camta1/2/3* mutants also respond differently to touching, e.g. response to distinct phytohormones (JA and ABA) and abiotic stresses (salt and wounding).

To further check if *mkk4/5* shares similarity in transcriptional changes with *camta1/2/3* in touch-induced response, we compared the transcriptional profiles of *mkk4/5* and *camta1/2/3* with the RNA-seq dataset of *myc2/3/4* (a JA-insensitive mutant) and the previously identified MYC2 touch regulon^20^ (Fig. 3b and Supplementary Data 7). The comparison showed that *mkk4/5* and *camta1/2/3* shared 550 similar DEGs (more than 55% of each dataset), whereas *mkk4/5* and *camta1/2/3* had 135 and 128 DEGs in common with *myc2/3/4*, respectively. Only 38 and 37 genes of MYC2 touch regulon were found in the DEGs of *mkk4/5* and *camta1/2/3*, respectively. These indicate that touch response regulated by MKK4/5 mostly resembles that regulated by CAMTA1/2/3. We also compared the RNA-seq datasets of *mkk4/5*, *camta1/2/3* and *myc2/3/4* with the list of 1168 “core early touch response” genes (Fig. 3c). Notably, we found that 215 “core early touch response” genes were not found in either the DEGs sets of *mkk4/5*, *camta1/2/3* or *myc2/3/4*, indicating that these 215 genes (~18% of 1168) are likely not touch regulated by either MKK4/5, CAMTA1/2/3 or MYC2/3/4 but are regulated by other unknown regulator(s). GO analysis of these 215 “core early touch response” genes (enriched GO terms with *p*_adj_ < 0.05) showed the enrichment of genes involved in regulation of circadian rhythm and photomorphogenesis, red and far-red light phototransduction, response to virus and abiotic stress (wounding, hypoxia, cold, heat and salt), DNA-binding transcription factor activity, and protein-containing complex (Fig. 3d and Supplementary Data 8).

To obtain a set of genes that is likely directly regulated by CAMTA1/2/3, we compared the 988 CAMTA1/2/3-regulated touch-responsive genes with a CAMTA3 chromatin immunoprecipitation sequencing (CHIP-seq) analysis previously done by Matsumura et al.^26^ (Fig. 3e). 301 genes were identified to be directly regulated by CAMTA3 and homologs, which we thus designated as the CAMTA1/2/3 touch regulon (Supplementary Data 9). Comparison of MKK4/5-touch regulated genes with the CAMTA1/2/3- and MYC2- touch regulons (Fig. 3e, f) showed that approximately half of the CAMTA1/2/3 touch regulon genes are also regulated by MKK4/5. Also, half of the MYC2 touch regulon overlapped with MKK4/5-induced touch genes, but the absolute number was quite low (38). Fifteen MYC2 regulon genes were also part of the CAMTA1/2/3 touch regulon. Approximately 80% (721 genes) of MKK4/5-touch regulated genes did not belong to the MYC2 or CAMTA1/2/3 regulons. These observations further indicate that MKK4/5 might cooperate with CAMTA1/2/3 in touch-induced response, and to a lesser extent with MYC2/3/4.

### Loss of MAPK kinases *MKK4* and *MKK5* leads to global impairment of touch-regulated protein phosphorylation

**Fig. 4:**
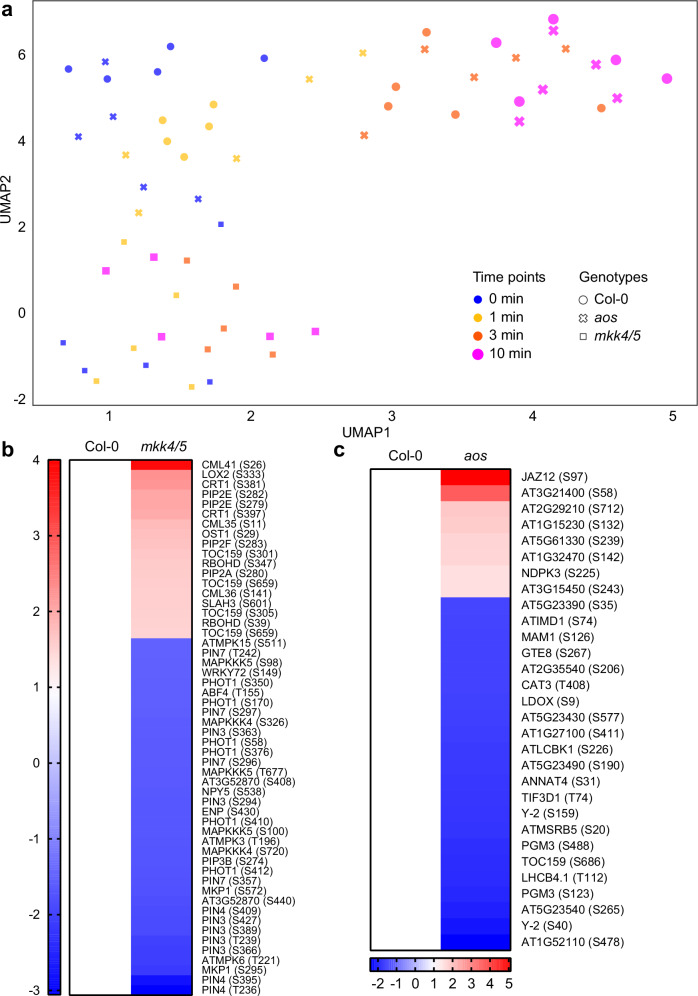
Loss of MAPK kinases *MKK4* and *MKK5* leads to global impairment of touch-regulated protein phosphorylation. **a** Uniform Manifold Approximation and Projection (UMAP) with imputed and corrected data points represents the global profiles of protein phosphorylation in each time point (0, 1, 3 and 10 min) of WT, *aos* and *mkk4/5*. **b** Heatmap representing selected differentially phosphorylated sites highlighted in *mkk4/5* compared to in Col-0. Color bar indicates linear fold changes. **c** Heatmap representing all the differentially phosphorylated sites in *aos* compared to in Col-0. Color bar indicates linear fold changes.

Given the important role for MKK4/5 in touch-responsive gene expression and that MKK4/5 are part of a phosphorylation signalling cascade, we performed a phosphoproteomic analysis to examine the global changes in protein phosphorylation after touching in the *mkk4/5* mutant. We also included the JA-deficient *aos* mutant, which has impaired touch-induced transcriptional response of JA-dependent genes (Fig. 1b), to examine if touch-regulated protein phosphorylation is in part controlled by JA. For the phosphoproteomic analysis, untouched (0 min) and touched (1, 3, or 10 min) plants (5 biological replicates/treatment) were collected, then subjected to tandem mass spectrometry (MS/MS) and statistical analyses to identify the changes in protein phosphorylation (Supplementary Data 10). We first used the Uniform Manifold Approximation and Projection (UMAP) method to visualize the major trends in phosphorylation patterns for each genotype after touching (Fig. 4a). The different replicates for the same time-points/genotypes clustered together well, supporting that the dataset is of high quality. We observed that touch treatment caused a clear change in protein phosphorylation in Col-0, especially at 3 and 10 min after treatment. The changes in phosphorylation after the touch treatment in *aos* were very similar to Col-0, suggesting that JA does not play a major role in early touch-regulated protein phosphorylation, despite its prominent role in touch-regulated gene expression (Fig. 1b)^20^. Strikingly, virtually no touch-induced alterations in global protein phosphorylation could be observed in *mkk4/5* mutants, indicating that MKK4/5 play a dominant role in touch-induced protein-phosphorylation.

The UMAP analysis indicated a basal change in protein phosphorylation in *mkk4/5* even before touch treatment, so we statistically examined the “genotype” effect on protein phosphorylation in mutants vs WT by two-way ANOVA (Supplementary Data 11 and Fig. 4b, c). In the *mkk4/5* mutant, there were 407 differentially phosphorylated sites on 290 proteins compared to Col-0 (Supplementary Fig. 5a and Data 11). Of these 407 phosphosites, there were 234 phosphosites on 159 proteins that were less phosphorylated whereas 173 phosphosites on 132 proteins were more phosphorylated than in the WT. Notably, loss of *MKK4/5* decreased phosphorylation of kinases (MPKKK4/5, MPK3/6, MPK15, phototropin protein (PHOT1)), phosphatase MKP1, auxin transporters (PIN3/4/7) and transcription factors (WRKY72 and ABF4) (Fig. 4b). Proteins involved in Ca^2+^ binding (CML41/35/36 and CRT1), chloroplast outer membrane translocon (TOC159), water channel activity (PIP2A/2E/2 F), JA-biosynthesis (LOX2) and respiratory burst oxidase homolog RBOHD were however more phosphorylated in the *mkk4/5* mutant (Fig. 4b). We only found 30 differentially phosphorylated sites on 27 different proteins affected by lack of JA in the *aos* mutant compared to in WT (Fig. 4c and Supplementary Data 11). Of these 30 phosphosites, 20 phosphosites on 22 proteins were less phosphorylated and 9 phosphosites on 8 proteins were more phosphorylated compared to WT. Interestingly, JAZ12 was more phosphorylated and proteins located in chloroplasts, e.g. transmembrane receptor TOC159 and light-harvesting protein LHCB4.1, were less phosphorylated. Only 10 phosphosites were commonly differentially phosphorylated versus WT in both mutants (Supplementary Fig. 5b), indicating there is a lack of overlap of protein phosphorylation between the *aos* and *mkk4/5* mutants. Thus, our results suggest that MKK4 and 5 control the basal phosphorylation state of several proteins, while the absence of JA has almost no impact on the phosphoproteome of Arabidopsis in control conditions.

### Touch treatment triggers dynamic changes in protein phosphorylation

**Fig. 5:**
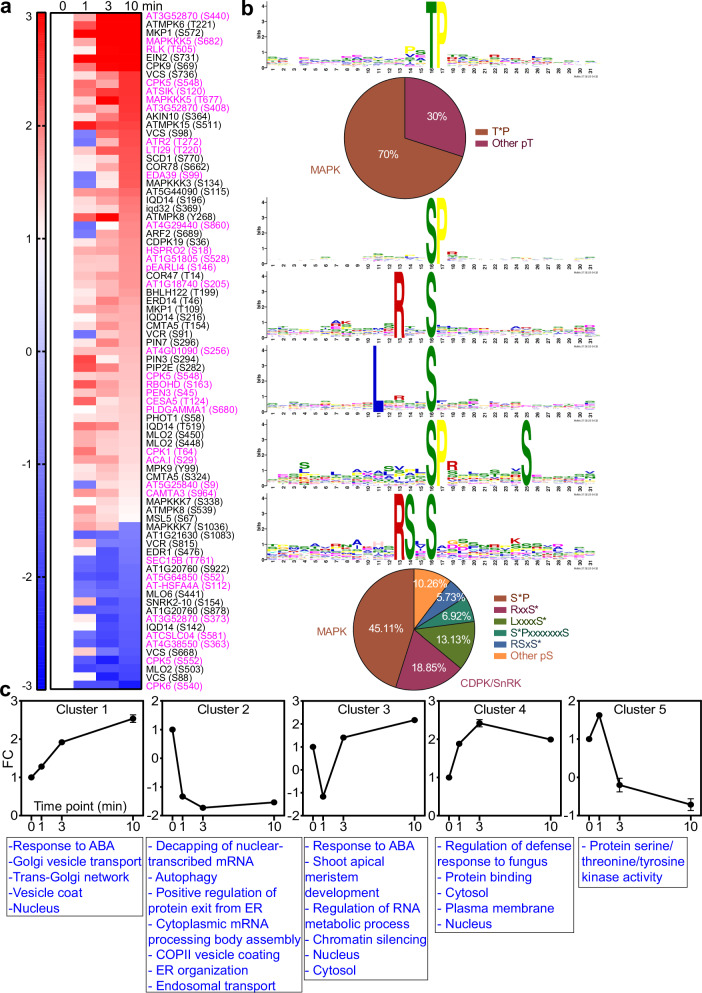
Protein phosphosites regulated by touch in plants. **a** Heatmap representing differentially phosphorylated sites in WT at 1, 3, and 10 min after touching compared to untouched condition. The “core early touch response” proteins are highlighted. Color bar indicates linear fold changes. **b** Phosphorylation motif analysis of touch-regulated phosphopeptides. The T*P, S*P, RxxS*, LxxxS*, S*PxxxxxxxS, and RSxS* motifs identified by the MoMo online motif search tool were illustrated using Weblogo^81^. The pie charts show that the MAPK motif T*P accounted for 70% of the phosphorylated Threonine (pT) population, and the MAPK motif S*P and the SnRK/CDPK motif RxxS* accounted for ~45% and ~19% of the phosphorylated Serine (pS) population, respectively. **c** Cluster analysis of differentially phosphorylated sites in WT at 0, 1, 3, and 10 min of touching. *n* = 5 biologically independent samples. Data are presented as mean ± SD. GO analysis shows the overrepresented protein functions in each cluster.

To identify phosphosites that are rapidly regulated by touch, we analyzed the phosphoproteome dataset by two-way ANOVA with only the time effect taken into consideration (Supplementary Data 12). For simplicity, we only present the phosphorylation profile of WT because genotype effects should not be considered in this case (Fig. 5 and Supplementary Data 12). We observed a gradual increase in the number of differentially phosphorylated sites compared to untreated plants (0 min) over time: 160, 321 and 380 phosphosites at 1, 3 and 10 min of touching, respectively (Supplementary Data 12). Differential phosphosites detected at 3 min showed the most similarity with those at 10 min (76% overlap), indicating that most changes in protein phosphorylation occur within 3 min and remain 10 min after touch treatment (Supplementary Fig. 5c).

We found that 488 protein sites were differentially phosphorylated by touching in WT during the time course (Supplementary Fig. 5d and Data 12). 383 of 488 differentially phosphorylated sites were more phosphorylated within 10 min after touching (Supplementary Fig. 5d and Data 12). These include phosphosites of kinases (MPK6/8/9/15, MAPKKK3/5/7, CPK1/5/9, CDPK19, ATSIK and AKIN10), phosphatase MKP1, calmodulin-binding proteins (CAMTA3/5 and IQD14/32), transcription factors (ARF2 and bHLH122), proteins involved in decapping nuclear-transcribed mRNA (VCS and VCR), proteins induced by low temperature (COR47/78), proteins involved in defense response (RBOHD and MLO2/6), phototropin proteins (PHOT1), auxin transporters (PIN3/4/7), and mechanosensitive channel protein MSL5 (Fig. 5a and Supplementary Data 12). 105 of 488 differentially phosphorylated sites were less phosphorylated within 10 min after touching, including phosphosites of kinases (MAPKKK7, EDR1, SNRK2.10 and CPK5/6), proteins involved in decapping nuclear-transcribed mRNA (VCS and VCR), transcription factor (HSFA4A), and calmodulin-binding protein in response to fungus (MLO2/6) (Fig. 5a and Supplementary Data 12). As RBOHD and PIN3/4/7 were significantly more phosphorylated in response to touch, we performed qPCR on the *rbohD*, *pin3-4* and *pin3/4/7* mutants to check if RBOHD and PIN3/4/7 play a role in touch response at transcript level (Supplementary Fig. 6). However, there were no significant changes in the touch responsive genes between the mutants and WT, except *CML40* and *ERF019* that were slightly less induced in the *pin3/4/7* mutant, suggesting that RBOHD and PIN3/4/7 are unlikely to play major roles in touch response in plants.

Protein kinases often have preferences to phosphorylate specific motifs in the target sequence. To extract phosphorylation motifs from the dataset, we searched the touch-regulated phosphopeptides using to the “Modification Motifs” (MoMo) motif search tool^40^ (Fig. 5b). We observed that the MAPK-associated motifs T*P and S*P^41^ accounted for the largest peptide populations, which are 70% and ~45% of the phosphorylated Threonine (pT) and Serine (pS), respectively (Fig. 5b). The SnRK/CDPK motif RxxS*^42^, accounted for ~19% of the pS population. Other unknown pS motifs, including LxxxS*, S*PxxxxxxxS and RSxS* accounted for ~26% of the pS population (Fig. 5b). Together, these findings further indicate that MAPKs play a major role in protein phosphorylation in plant touch response.

To better understand the touch-regulated phosphosites, we conducted a cluster analysis followed by GO analyses of the proteins that have differentially phosphorylated sites after touching (Fig. 5c and Supplementary Data 13, 14). This analysis resulted in 5 clusters representing 5 different patterns of protein phosphorylation at different time points of touching. Cluster 1 showed that proteins involved in ABA response, and Golgi vesicle coat and transport were phosphorylated increasingly over time. In contrast, cluster 2 showed a dramatic decrease in phosphorylation within 1 min, then this phosphorylation remained low until 10 min. This cluster consisted of proteins involved in decapping nuclear-transcribed mRNA, autophagy, positive regulation of protein exit from ER, cytoplasmic mRNA processing, vesicle transport, ER organization and endosomal transport. Cluster 3 also showed a rapid reduction in phosphorylation within 1 min, but this phosphorylation recovered at 3 min and increased slightly at 10 min. Phosphorylated proteins in this cluster were involved in ABA response, shoot apical meristem development, regulation of RNA metabolic process and chromatin silencing. Cluster 4 partially resembles cluster 1, showing protein phosphorylation increased within 3 min, but then decreased at 10 min. This cluster is composed of proteins involved in regulation of defense response to fungus and protein binding. Cluster 5 showed that kinases had their phosphorylation increased rapidly within 1 min then substantially decreased at 3 and 10 min of touching.

### MKK4/5 are required for MPK3/6 phosphorylation after mechanical treatment

In the previous section, we explored how WT responds to touching at a phosphoproteome level. To examine the effect of JA-deficit or impairing MKK4/5 on protein phosphorylation after touch treatment, we analyzed the phosphoproteome dataset considering the interaction between the genotype and time effect by two-way ANOVA (Supplementary Fig. 7 and Data 15). We found that 112 and 241 protein sites were differentially phosphorylated in *aos* and *mkk4/5* after touching compared with WT, respectively. There were 73 phosphosites in common between *aos* and *mkk4/5*, which accounted for ~65% and ~30% of differentially phosphorylated sites of *aos* and *mkk4/5*, respectively (Supplementary Fig. 7a). When we applied a cutoff for fold change (mutant/WT > 1.5 or < 0.6666), only 15, 18, and 12 differentially phosphorylated sites remained in *aos* at 1, 3, and 10 min, respectively, compared to 39, 79, and 115 in *mkk4/5* (Supplementary Data 15). Additionally, there was not much similarity between these mutants at each time point (Supplementary Fig. 7b and Data 15). These findings indicate that MKK4 and MKK5 are key regulators of protein phosphorylation and subsequent transcriptional regulation in response to touch, whereas JA does not seem to be required for the bulk of touch-induced phosphorylation during the first 10 min after treatment.

**Fig. 6:**
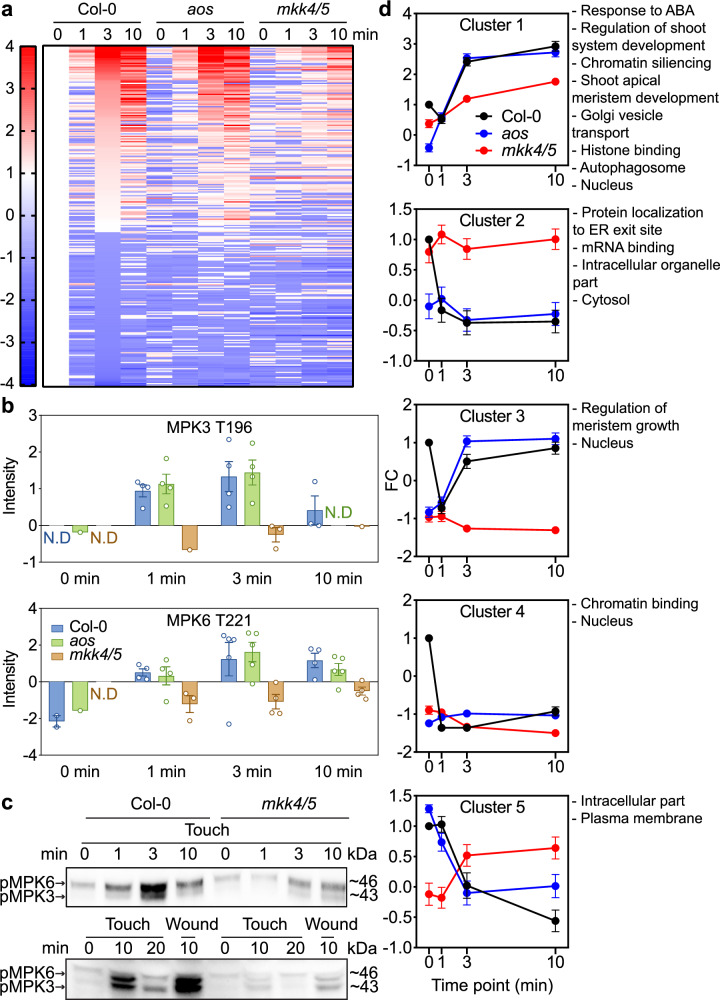
Impairment of *MKK4/5* leads to a reduction in protein phosphorylation in response to touch. **a** Heatmap representing differentially phosphorylated sites due to interaction effect in WT, *aos* and *mkk4/5* at 1, 3 and 10 min after touching compared to untouched condition (0 min). **b** Phosphorylation changes of MPK6 at threonine 211 (T221) and MPK3 at threonine 196 (T196) representing the non-imputed data. *n* = 5 biologically independent samples. Data are presented as mean ± SE. Each dot indicates a biological replicate. N.D.=not detected. **c** Immunoblots using an antibody against phosphorylated MAPKs in WT and *mkk4/5* before and after touch or wounding treatment. Similar results were obtained in three independent experiments. **d** Cluster analysis of differentially phosphorylated sites due to interaction effect in WT, *aos* and *mkk4/5* at 0, 1, 3 and 10 min of touching. *n* = 5 biologically independent samples. Data are presented as mean ± SD. GO analysis (*p *< 0.05) was done to show the protein functions in each cluster.

We created a heatmap to visualize the protein phosphorylation patterns between genotypes over time during touching (Fig. 6a and Supplementary Data 15). We observed a highly similar pattern between WT and *aos*, indicating that JA-defect does not strongly affect protein phosphorylation in response to touch. In contrast, *mkk4/5* showed a clear suppression of protein phosphorylation at 3 and 10 min compared to WT, supporting that loss of *MKK4/5* results in significant phosphorylation changes in response to touch.

Since MKK4/5 are thought to phosphorylate MPK3/6 in a variety of conditions^43^, we examined the changes in protein phosphorylation of MPK3/6. To get a better view on the actual dynamic range in phosphorylation changes, we presented the non-imputed normalized phosphoproteome values of MPK6 at threonine 211 (T221) and MPK3 at threonine 196 (T196) (Fig. 6b). We observed that the phosphorylation of MPK3/6 was very similar in WT and *aos*, whereas *mkk4/5* showed a strong decrease in MPK3/6 phosphorylation compared to WT. To verify the results of our phosphoproteomic data, we performed immunoblotting using an antibody against phosphorylated MPKs (Fig. 6c and Supplementary Fig. 7c). Wound treatment was included as a positive control since MPK3 and MPK6 are phosphorylated by MKK4 and MKK5 in response to wounding^33^. In WT, touching resulted in the rapid phosphorylation of MPK3 and MPK6 after 1 min. This phosphorylation lasted until approximately 10 min but largely disappeared after 20 min. Wounding also led to strong MPK3/6 phosphorylation after 10 min. In contrast, MPK3/6 phosphorylation was abolished in *mkk4/5* after touching and wounding (Fig. 6c). In summary, MKK4 and MKK5 phosphorylate MPK3 and MPK6 in response to touch in plants.

To gain further insight into changes in protein phosphorylation between genotypes after touching, we performed a cluster analysis of proteins that have differentially phosphorylated sites in response to touch in the mutants. We identified 5 clusters representing different patterns of protein phosphorylation between genotypes at each time point after touching (Fig. 6d and Supplementary Data 16, 17). In general, we observed a similar pattern of protein phosphorylation between WT and *aos* in all clusters. However, *mkk4/5* showed a very different pattern of protein phosphorylation compared to WT in all clusters. This further supports that protein phosphorylation in response to touch is much more affected by impairment of MKK4 and MKK5 than by JA-deficiency. GO analysis of each clusters showed that proteins involved in response to ABA, regulation of shoot system development, chromatin silencing, histone binding, mRNA binding, Golgi vesicle transport, autophagosome were more phosphorylated after 3 min-touched in WT and *aos* than in *mkk4/5* (Cluster 1). Proteins related to protein localization to ER exit site and mRNA binding were less phosphorylated after touching in WT and *aos*, but their phosphorylation remained largely unchanged in *mkk4/5* (Cluster 2).

### MAPKKK3, MAPKKK5 and YODA (MAPKKK4) are the upstream regulators of the MKK4/5-MPK3/6 cascade

**Fig. 7:**
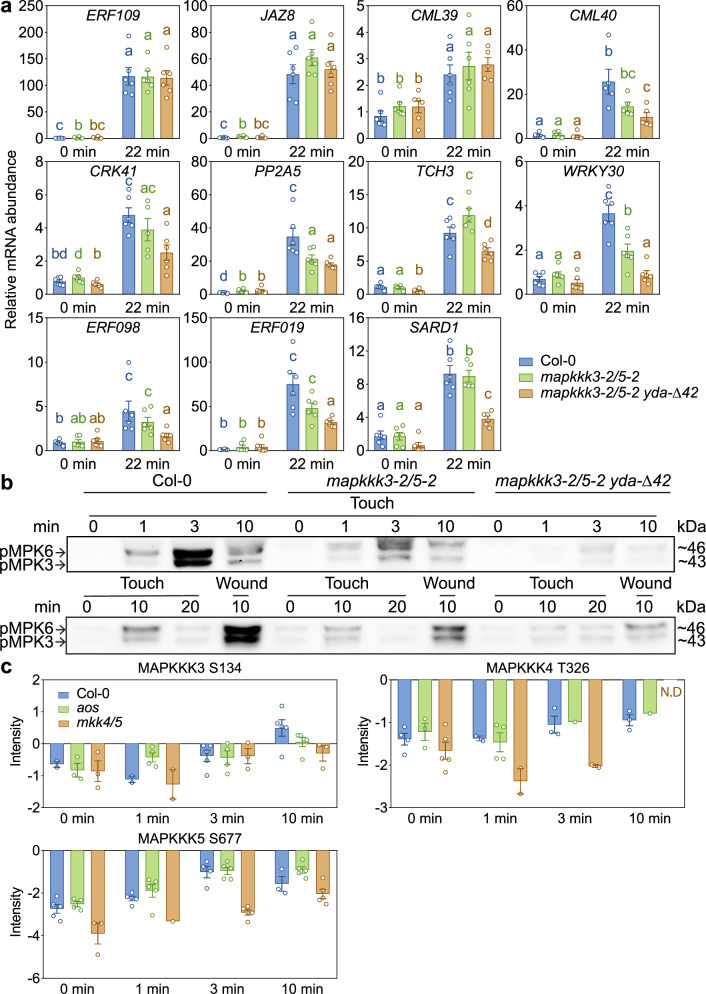
MAPKKK3/4/5 are upstream regulators of the MKK4/5-MPK3/6 cascade in plant touch response. **a** Analysis of touch marker gene expression in Col-0 (WT), *mapkkk3-2/5-2* and *mapkkk3-2/5-2 yda-∆42* mutants. 12-day-old seedlings before (0 min) and 22 min after touching by gentle brushing were collected for qRT-PCR. The y axis represents the relative mRNA level. Data are presented as mean ± SE. *n* = 6 biologically independent samples. Each dot indicates a biological replicate. Statistical significance was based on Kruskal-Wallis test followed by Wilcoxon rank sum tests. Different letters represent the significant differences between genotypes (*p* < 0.05). **b** Immunoblots using an antibody against phosphorylated MAPKs on WT, *mapkkk3-2/5-2* and *mapkkk3-2/5-2 yda-∆42* mutants before and after touch or wounding treatment. Similar results were obtained in three independent experiments. **c** Phosphorylation changes of MAPKKK3 at serine 134 (S134), MAPKKK4 at threonine 326 (T326) and MAPKKK5 at serine 677 (S677) using the non-imputed data. *n* = 5 biologically independent samples. Data are presented as mean ± SD. Each dot indicates a biological replicate. N.D = not detected.

The previous sections provided clear evidence that MKK4/5 are important for touch responses and responsible for MPK3/6 phosphorylation after touching. We therefore wanted to find the upstream MAPKKKs that are responsible for MKK4/5 activation. To do this, we further explored our phosphoproteomic data and identified that MAPKKK3, MAPKKK5, YODA (MAPKKK4/YDA), MAPKKK7 and MAPKKK9 were all differentially (usually more) phosphorylated within minutes after touching (Supplementary Data 10). To assess whether some of these identified MAPKKKs are indeed involved in touch response, we obtained *mapkkk3/5* double and *mapkkk3/5/yda-Δ42* triple mutants (with a 42 amino acid deletion in YDA)^43^. We first performed a touch experiment to assess transcriptional changes within 22 min (Fig. 7a). No effects on touch-induced expression of JA-dependent genes (*ERF109*, *JAZ8* and *CML39*) were observed in any of the mutants (Fig. 7a). However, induction of CAMTA1/2/3-regulated (JA-independent) genes, including *WRKY30* and *PP2A5*, was impaired in the *mapkkk3/5* double mutant. Additional loss of function of *YDA* (*mapkkk3/5/yda-Δ42*) resulted in an even stronger loss of induction of *WRKY30* and *PP2A5*, and also impaired loss of induction for *CRK41*, *TCH3*, *ERF098*, *ERF019*, *CML40*, and *SARD1* (Fig. 7a).

There are approximately 80 MAP3Ks, 10 MAP2Ks, and 20 MAPKs in Arabidopsis^44–46^, thus redundant functions could occur. Therefore, we also carried out qPCR on other *MAPKKK* mutants, including *m3kδ1/δ5-1/δ6-1/δ7* and *m3kδ1/δ5-2/δ6-1/δ7*^47^, to check if MAPKKK *δ*1, 5, 6 and 7 play roles in response to touch (Supplementary Fig. 8a). *TCH2* was significantly less induced in *m3kδ1/δ5-2/δ6-1/δ7* but there were no significant changes in other touch responsive genes between the mutants and WT, suggesting that MAPKKK *δ*1, 5, 6 and 7 are unlikely to be major players in plant touch response.

We suspected that MPK3/6 phosphorylation should then be downstream of MAPKKK3/5/YDA, so we performed immunoblots against phosphorylated MPK3/6 in response to touching and wounding (Fig. 7b and Supplementary Fig. 8b). Indeed, loss of *MAPKKK3/5* resulted in a reduction of touch/wounding-induced MPK3/6 phosphorylation (Fig. 7b). Furthermore, MPK3/6 phosphorylation was almost completely abolished in the *mapkkk3/5/yda-Δ42* triple mutant. Interestingly, phosphosites on MAPKKK3/4/5 themselves (MAPKKK3 at serine 134, MAPKKK4 at threonine 326 and MAPKKK5 at serine 677) were less phosphorylated after touching in *mkk4/5* mutants, indicating a phosphorylation feedback loop (Fig. 7c) (Supplementary Data 10). In summary, our data show that a MAPKKK3/4/5-MKK4/5-MPK3/6 cascade plays a key role in touch-induced protein phosphorylation and subsequent JA-independent gene expression changes.

### MAPKKK3/4/5 are required for normal thigmomorphogenesis

**Fig. 8:**
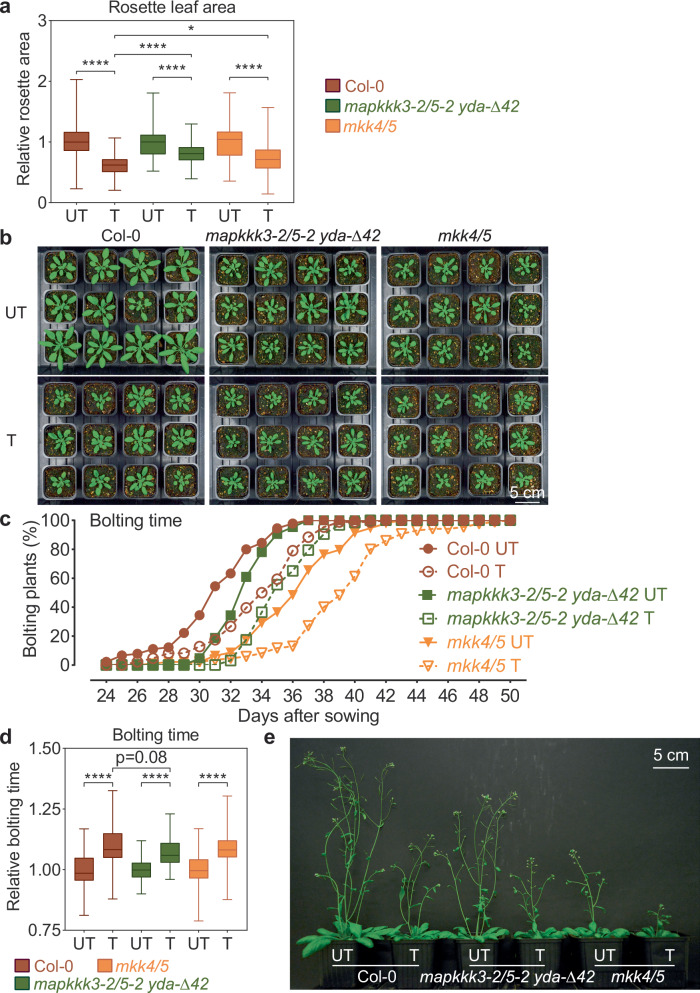
MAPKKK3/4/5 are required for normal thigmomorphogenesis. **a** Box and whisker plot represents relative rosette leaf area of 28-day-old plants, either untouched or after 14 days of regular touching. *n* = 63–93 plants (*=*p* < 0.05, ***=*p* < 0.001, ****=*p* < 0.0001). A box plot shows the minimum and maximum values (whiskers), the 25th and 75th percentiles (box bounds), and the median (center line). **b** A representative picture of 28-day-old untouched plants or plants in which touch treatment had been applied twice-daily for 14 days. Scale bar = 5 cm. **c** Line graph showing the percentage of bolting plants over the growth period (days after sowing). **d** Box and whisker plot represents relative bolting time under untouched and touched conditions. *n* = 63–93 plants (*=*p* < 0.05, ***=*p* < 0.001, ****=*p* < 0.0001). Box plot shows the minimum and maximum values (whiskers), the 25th and 75th percentiles (box bounds), and the median (center line). **e** A representative picture of 41-day-old untouched plants or plants in which touch treatment had been applied twice–daily for 27 days. Scale bar = 5 cm. UT = untouched, T = touched.

Regular touch treatment leads to a delay in plant bolting and decrease in rosette leaf size^9^. Therefore, we wanted to examine the effect of *mapkkk3-2/5-2 yda-∆42* and *mkk4/5* mutations on thigmomorphogenesis (Fig. 8). Two-week-old soil-grown seedlings were touched mildly by a brush twice a day until the plants had bolted. After 2 weeks of touching, WT plants showed significantly reduced rosette leaf area by ~38% compared to untouched WT plants (Fig. 8a, b). However, in *mapkkk3-2/5-2 yda-∆42* rosette size after regular touching was only reduced by ~20% compared to untouched plants, which was a significantly smaller reduction than in WT (*p* < 0.0001) (Fig. 8a, b). *mkk4/5* showed an intermediate phenotype, with a relative reduction in rosette size by only ~28% (Fig. 8a, b). Touched WT plants showed a delay in bolting time of around three days (Fig. 8c, d, e). We observed that the relative touch-induced delay in bolting time in *mapkkk3-2/5-2 yda-∆42* tended to be smaller than in WT (*p* = 0.08) (Fig. 8c, d, e). *mkk4/5* plants overall flowered later than WT, which may be due to their slower development^33,38^ (Fig. 8c, d, e). Nevertheless, a clear delay in flowering could still be observed in the touched *mkk4/5* mutant, that was similar to that in WT (Fig. 8c, d, e). In addition, we observed a clear delay in flowering and smaller rosette size in the touched *mpk6-3* mutants and *MPK6SR* lines, which resemble WT, suggesting that MPK3 and MPK6 redundantly respond to thigmomorphonenesis (Supplementary Fig. 9). In conclusion, these findings demonstrate that the MAPKKK3/4/5-MKK4/5 cascade is required for normal thigmomorphogenesis.

## Discussion

In this study, we expose the major role of MAPK signalling cascades in touch response. The qRT-PCR and RNA-seq data showed that mostly JA-independent touch responsive genes were less induced by touch after 22 min in the *mkk4/5* mutant, indicating that MKK4/5 is a novel regulator of touch response in a largely JA-independent manner. The phosphoproteome analysis further showed the major impact that MKK4/5 have on touch-induced phosphorylation, suggesting they are the main players in early touch-induced phosphoproteome changes. Although a previous study had shown that loss of JA-signalling (in the *myc2* mutant) had significant effects on JA-induced phosphoproteome changes 2 h after JA treatment^48^, our data strongly suggest that JA is not involved in the early phosphoproteome changes occurring within 10 min of touching (as observed in the *aos* mutant). It is not fully clear if rapid activation of the JA pathway requires phosphorylation, but our data did not show strong evidence of touch-induced phosphorylation of JA signalling components, except that less JAZ12 phosphopeptides were measured after touching. The MKK4/5-regulated pathway at least does not seem to be required for full activation of the JA signalling pathway. In addition, our results indicate that there is no clear evidence that MKK3 is required for JA-dependent pathways to be activated in response to touch, although MKK3 phosphorylation is activated through wound-induced JA production^33^.

Comparison of *mkk4/5*, *camta1/2/3* and *myc2/3/4* RNA-seq datasets showed a major overlap between *mkk4/5* and *camta1/2/3* (550 common DEGs), but only 135 common DEGs between *mkk4/5* and *myc2/3/4*. This suggests that MKK4/5 may cooperate with CAMTA1/2/3 to regulate touch signalling to a larger extent than MKK4/5 with MYC2/3/4. How MKK4/5 cooperate with CAMTA1/2/3 is, however, unclear. We initially hypothesised that MKK4/5 might phosphorylate MPK3/6, which in turn directly phosphorylate and activate CAMTA1/2/3, thereby activating the expression of JA-independent touch responsive genes. Jiang et al.^49^ showed that CAMTA3 is phosphorylated by MPK3 and MPK6 at multiple targeted sites, leading to CAMTA3 destabilization after flagellin 22 (flg22) treatment. Of these flg22-affected MPK3/6-targeted phosphorylation sites of CAMTA3, S8 and S587 were detected in our data, but only S587 was slightly less phosphorylated 10 min after touching (0.7× after 3 min). CAMTA3 S587 was also slightly less phosphorylated (0.89×) in *mkk4/5*, but did not pass statistics for the combined time × genotype effect. Previous studies showed that mutation of S587 did not show significant effects on MPK3/6-dependent phosphorylation of CAMTA3, even in combination with 4 other MPK3/6 phosphorylation site mutations^49^. Opposite to flg22 treatment, cold stress did not lead to CAMTA3 phosphorylation and destabilization, yet even seemed to enhance CAMTA3 stability, indicating that regulation of CAMTA3 activity and stability is variably determined by different stress contexts^49^. CAMTA3 phosphosite S964, which was shown to be directly phosphorylated by CPK5 leading to its protein instability in effector-triggered immunity (ETI)^50^, was more phosphorylated by touching (up to 1.8×) in our data, yet not affected by loss of MKK4/5 function. In contrast, the CAMTA3 T586 phosphosite was mildly affected by loss of MKK4/5, but did not respond to touching. No phospho-sites for CAMTA1 were detected in our study. For CAMTA2, a S984 phosphosite was found that did not show touch- or genotype- dependent changes, while three CAMTA5 phosphosites were more phosphorylated by touching but were unaffected by any of the genotypes. Taken together, there is no clear evidence that CAMTA1/2/3 are direct substrates of MKK4/5 or MPK3/6 in response to touch. It is possible that cold stress response is alike to touch response, in which phosphorylation of CAMTA3 enhances its stability to regulate downstream touch responsive genes. Nevertheless, upstream regulator(s) of CAMTA3 phosphorylation in response to touch are still unknown.

A previous study also reported a phosphoproteome 45 s after brushing in WT Arabidopsis^25^, so we compared both early phosphoproteome datasets (Supplementary Data 18). Of the 2,525 phosphorylated proteins identified by Wang et al.^25^, 268 were also found as differentially phosphorylated by touching in our data. Interestingly, MAPKKK3/5/7, several MPKs and calmodulin-domain protein kinase (CPKs) were common to both datasets, suggesting they are consistently touch-phosphorylated proteins. The study by Wang et al.^25^ also identified TREPH1 as a touch-phosphorylated protein. In our data, we observed seven phosphosites on TREPH1, of which one (S367) was slightly less phosphorylated at 3 min after brushing. We also identified the TREPH1 phosphosite at S625, which was suggested by Wang et al.^25^ as being important for TREPH1 function and subsequent effects on gene expression and thigmomorphogenesis. However, we did not find evidence for touch-induced changes in TREPH1 S625 phosphorylation. For five of the TREPH1 phosphosites (including S625), we found slight but significantly lower overall phosphorylation in the *mkk4/5* mutant, but no evidence for touch-induced phosphorylation changes. Based on our data here and previously reported^19^, we did not find strong evidence for TREPH1 as a target of touch-induced phosphorylation and downstream touch-responsive regulation.

By mining the phosphoproteomic data, we found that MAPKKK3/4/5 are phosphorylated within minutes after touching. Transcript analysis in *mapkkk3/4/5* triple mutants revealed that MAPKKK3/4/5 regulate the expression of overlapping JA-independent touch genes with MKK4/5 and MPK3/6. Notably, our immunoblot analysis showed that MAPKKK3/4/5 are also the previously unknown upstream regulators of MKK4/5 and MPK3/6 in wound response. The touch-phosphorylated amino acids T196 (MPK3) and T221 (MPK6) identified in our study are both within the predicted activation loop^51^, supporting that touching indeed activates the kinase activity of MPK3/6. In our phosphoproteomic data, MKK5 itself was also significantly more phosphorylated within 1 min after touching on serine S12 (MKK4 could not be detected).

MAPKKK3/4/5 were found to be less phosphorylated after touching in the *mkk4/5* mutant, indicating that MAPKKK3/4/5 are themselves targets of MKK4/5. This further indicates that a phosphorylation feedback loop occurs in response to touch. This could be a positive feedback loop, which reinforces the signalling cascade, or a negative feedback loop, which turns off the cascade relatively fast to avoid unnecessary false alarms. Previous works showed that the activated MPK3 and MPK6 can phosphorylate MAPKKK5 at serine 682/692 (S682/692) and serine 90 (S90), respectively, to enhance the MAPK cascade activation and immunity^52,53^. MAPKKK5 S682 was found to be significantly more phosphorylated within 10 min in WT and significantly less phosphorylated in the *mkk4/5* mutant compared to WT. MAPKKK5 S90 was, however, not differentially phosphorylated in our data. Additionally, EDR1, which may negatively regulate MAPK cascade activation by repressing the MPK3-mediated feedback regulation of MAPKKK5^53^, was less phosphorylated in response to touch within 10 min. Altogether, these observations suggest that a positive feedback mechanism enhances the signalling cascade activity in response to touch.

In Arabidopsis, the MAPKKK4-MKK4/5-MPK3/6 and MAPKKK3/5-MKK4/5-MPK3/6 cascades apparently have common MKKs and MPKs, but do not necessarily collaborate. The MAPKKK4-MKK4/5-MPK3/6 cascade is involved in plant development by negatively regulating stomatal production^54^, whereas the MAPKKK3/5-MKK4/5-MPK3/6 cascade is involved in plant immune response^52,55^. As reduction of MAPKKK4/YODA activity results in an increase in MPK3 and MPK6 activation induced by flg22, these two MAPK cascades thus have an antagonistic interaction under specific circumstances, most likely because MAPKKK3/5 and MAPKKK4/YODA might compete for downstream MKKs and MPKs^55^. Interestingly, our study suggests that MAPKKK3/5 and MAPKKK4/YODA cooperate in touch response, as MAPKKK3/5 has an intermediate effect on transcriptional response and MPK3/6 phosphorylation, whereas the triple mutant with also reduced MAPKKK4/YODA activity results in a much stronger effect, nearly completely blocking MPK3/6 phosphorylation after touch. Such an overlapping function of MAPKKK4 and MAPKKK3/MAPKKK5 as upstream regulators of MPK3/6 in plant immunity, growth and development has also been described previously^43,56^. We thus propose that MAPKKK3/4/5-MKK4/5-MPK3/6 is likely one cascade in response to mechanostimulation, including touching and wounding. Other MAPKKKs may also be involved in touch signalling such as MAPKKK7/9, which were also phosphorylated after touching, or perhaps kinases involved in rapid auxin signalling like rapidly accelerated fibrosarcoma (RAF)-like protein kinases^57^. MAPKKK δ1, 5, 6 and 7 do not appear to play an obvious role in touch signalling, however (Supplementary Fig. 8a).

As MAPKKK3/5 and MAPKKK4 are likely to redundantly regulate downstream MKK4/5 and MPK3/6 in mechanostimulation, the question arises as to what the upstream regulators are that can activate MAPKKK3/4/5-MKK4/5-MPK3/6 as one cascade in response to mechanostimulation. Indeed, very little is known about the upstream regulators activating MAPKKK3/4/5. The receptor-like cytoplasmic kinase (RLCK) BSK1 phosphorylates MAPKKK5 at S289 in vitro^58^, which was not found in our phosphoproteomic data. Another RLCK PBL27 phosphorylates MAPKKK5 at 6 amino acid residues in vitro^59^, including T677, which was differentially phosphorylated in the *mkk4/5* mutant after touching. However, Sun et al.^55^ could not observe a decrease in MAPK activation and defense gene expression in the *pbl27* mutant plants in immune response. The MAPKKK4-MKK4/5-MPK3/6 cascade can be activated by recognition of peptide ligands EPF1 and EPF2 by receptor complexes including the ERECTA family of receptor-like kinases (ER, ERL1, and ERL2), the receptor protein TMM and SERK family receptor-like kinases on the plasma membrane^54^. The MAPKKK4-MKK4/5-MPK3/6 cascade can also be triggered by an increase in brassinosteroid (BR) level that is recognized by the BR receptor BRI, leading to BIN2 inactivation, which results in activation of MAPKKK4 and downstream MKKs and MPKs^60^. The upstream regulators of MAPKKK3 seem not to be identified yet. We hypothesize that mechanical stimulation could be perceived by mechanoreceptors, leading to activation of RLCKs, which then triggers the MAPKKK3/4/5-MKK4/5-MPK3/6 as one cascade responding to touch.

Finally, thigmomorphogenesis analysis by repetitive touching showed that MKK4/5 and especially MAPKKK3/4/5 are required for normal reduction in rosette size. In addition, the *mapkkk3-2/5-2 yda-∆42* mutants showed less touch-induced delay in bolting time compared to touched WT. These results show that the MAPKKK3/4/5-MKK4/5 cascade is required for normal thigmomorphogenesis. This is comparable to *camta1/2/3*, *aos* and *myc234* mutants, which also show reduced thigmomorphogenesis^9,19^. MAPKKK3/4/5 and MKK4/5 are well-known for their important roles in plant defense responses^38,61^, so we propose that the MAPKKK3/4/5-MKK4/5-MPK3/6 cascade within touch response may affect both thigmomorphogenic and defence responses. This is further supported by the strong overrepresentation in MKK4/5 controlled GO categories involved in defense against biotic stresses. The phosphoproteomic data presented here will thus be a resource for functional analysis to find important downstream phosphosites that execute touch-induced effects on gene expression, plant growth and defence.

**Fig. 9:**
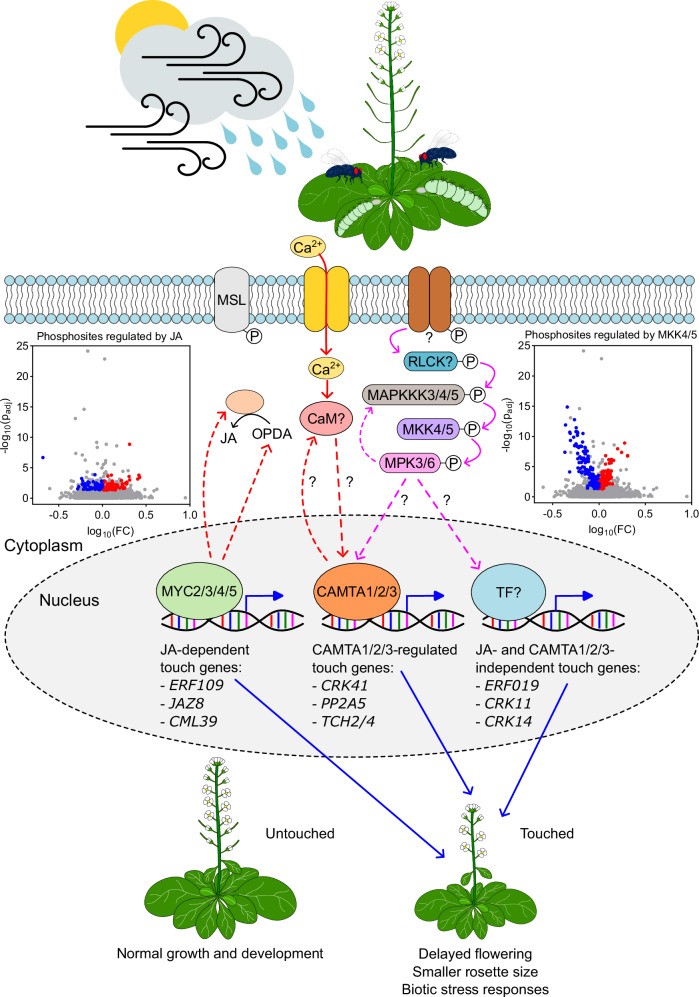
A model of touch-induced response in plants. Plants frequently encounter mechanical stimulations, such as wind, rain and insect herbivory. Mechanical stimulation may be perceived by plasma membrane-associated proteins, such as mechanosensitive channel-like (MSL) protein. Calcium channels activated by mechanostimulation lead to Ca^2+^ influx from the apoplast to the intracellular space. Ca^2+^ influx might be recognized by camodulins (CaM), which in turn may activate the activity of downstream regulators, such as the transcription factors CAMTA1/2/3, leading to the activation of JA-independent touch genes, e.g. *CRK41*, *PP2A5* and *TCH2/4*. Mechanostimulation also results in an increase in intracellular JA concentration, leading to the activation of the transcription factors MYC2/3/4/5, which in turn express JA-dependent touch genes, e.g. *ERF109*, *JAZ8* and *CML39*. Simultaneously, mechanical stimulation triggers a sequential MAPKKK3/4/5-MKK4/5-MPK3/6 phosphorylation cascade, which might be activated by receptor-like cytoplasmic kinase (RLCK) phosphorylation. Phosphorylated MPK3 might phosphorylate MAPKKK5 as a positive feedback loop that enhances the signalling cascade activity in touch response. Phosphorylated MPK3/6 can activate downstream regulators, which then activate the expression of JA- and CAMTA1/2/3-independent touch genes, e.g. *ERF019*, *CRK11* and *CRK14*. The volcano plots (red=more differentially phosphorylated, blue=less differentially phosphorylated, gray=not differentially phosphorylated) indicate that MKK4/5 are the main players in early mechanically-induced phosphorylation changes, whereas JA may play no or only a minor role in early touch-induced phosphoproteome changes. Overlaps among these three touch signalling pathways occur, but the cascade MAPKKK3/4/5-MKK4/5-MPK3/6 appears to cooperate with CAMTA1/2/3 to a larger extent than with MYC2/3/4/5 in touch-induced response. Collectively, all these touch-induced signalling pathways can lead to several thigmomorphogenic and biotic defense responses in plants.

Rapid activation of MPK6 by mechanical stimuli was already reported 26 years ago^30^, however, the mechanism and function of this phenomenon has remained unknown. Here we report that mechanical stimulation activates a MAPKKK3/4/5-MKK4/5-MPK3/6 cascade within 60 seconds, which leads to the induction of nearly 800 genes, encompassing the majority of the core early touch response. Most of these genes overlap with the touch-responsive genes regulated by CAMTA1/2/3, suggesting an interplay between MAPKs and CAMTAs. Clear evidence for MKK4/5 phospho-regulation of CAMTA1/2/3 could, however, not be obtained, suggesting that the potential functional interaction happens via intermediate regulatory mechanisms, or that both pathways independently converge and co-regulate the same genes (Fig. 9). This may explain why most genes do not completely lose touch-induction in *camta1/2/3* or *mkk4/5* mutants. The MAPKKK3/4/5-MKK4/5-MPK3/6 cascade also seems to partly overlap with the JA-pathway, but many prominent JA-regulated genes respond normally to touch in *mkk4/5* and *mapkkk3-2/5-2 yda-∆42* mutants. We also could not find clear evidence for a role of phosphorylation in early activation of the JA signalling pathway, nor did loss of JA affect the overall early touch phosphoproteome. The identification of this regulatory cascade brings our knowledge closer to the most upstream touch sensors and signal transducers, though these seem to remain elusive for now.

## Methods

### *Arabidopsis thaliana* genotypes

*Arabidopsis thaliana* Col-0 (WT) and mutants were either obtained from the Nottingham Arabidopsis Stock Centre (NASC) or from other research groups. *camta1/2/3* triple mutants were kindly provided by Dr. Michael F. Thomashow (Michigan State University), *rbohD* mutants were kindly provided by Dr. Michael Wrzaczek (Czech Academy of Sciences), *pin3-4* mutants were kindly provided by Dr. Stephanie Robert (Umeå University), *pin3/4/7* (*pin3-3 pin4-2 pin7*^*En*^) were kindly provided by Dr. Enrico Scarpella (University of Alberta) and Dr. Cezary Waszczak (University of Helsinki), *m3kδ1/δ5-1/δ6-1/δ7* and *m3kδ1/δ5-2/δ6-1/δ7* quadruple mutants were kindly provided by Dr. Julian Schroeder (University of California – San Diego), *mkk4/5* double and *mkk3-1* single mutants were kindly provided by Dr. Jean Colcombet (INRAE), *mkk3/4/5* triple mutants were obtained by crossing *mkk4/5* and *mkk3-1*. The mutants *mapkkk3-2/5-2*, *mapkkk3-2/5-2 yda-∆42*, and *MPK6SR* lines (*MPK6SR31* and *MPK6SR58*) were kindly provided by Dr. Shuqun Zhang (University of Missouri). The *aos* and *mpk6-3* mutants were described previously^39,62^.

### Plant growth conditions and touch treatment

Seeds were surface sterilized by gas sterilization overnight. Sterilized seeds were sown on half-strength MS agar plates (MS salt mixture (Duchefa, M0221.0050), 0.05% (w/v) MES (Biomol, 6010.100), 1% (w/v) sucrose (Duchefa, S0809.5000), 0.8% (w/v) phytoagar (Duchefa, P1003.1000), pH 5.7). The plates were stratified at 4 °C in the dark for 2 days prior to transferring to the growth chamber. Plants were grown under long-day conditions (16 h light/8 h dark, approximately 120 µmol photons m^−2^ s^−1^). Touch treatment was performed on seedlings as previously described^19^. Briefly, 12-day-old seedlings were gently brushed ~10 times using a soft paintbrush, taking about 30–45 s per plate. The pressure of the brushing was approximately 2 g/cm^2^. For *MPK6SR31* and *MPK6SR58* lines, seeds were sown on half-strength MS agar plates containing either DMSO (mock) or 1 µM NAPP1.

### qRT-PCR

Twelve-day-old seedlings grown on half-strength MS plates were collected at 0 and 22 min after touching, snap-frozen in liquid nitrogen and stored at -80 ⁰C until use. Approximately 5 to 6 seedlings were sampled for one biological replicate and 5 to 6 (*n* = 5-6) biological replicates were used for statistical analysis. Total RNA was isolated using the NucleoSpin™ RNA Plant Kit (Macherey-Nagel, 12721021). cDNA synthesis and qRT-PCR was carried out as previously described^63^ by using the iScript™ cDNA Synthesis Kit (BioRad, 1708891) and CFX384 Real-time PCR Detection System (BioRad) using Sso Advanced Universal SYBR green detection assays (BioRad, 172-5271), respectively. *UBIQUITIN-CONJUGATING ENZYME 21/PEROXIN4* (*UBC21/PEX4*) was used as the house keeping gene for data normalization. All qPCR experiments used CML39 F1 and R1 primers, except that qPCR experiments on *MPK6SR* lines, *pin3-4* and *pin3/4/7* used CML39 F2 and R2 primers. All primers were listed in Supplementary Dataset 19. Because the data were not normally distributed, statistical tests were performed using Kruskal-Wallis test followed by Wilcoxon rank sum tests. Different letters representing the significant differences between genotypes (*p* < 0.05) were obtained using R.

### RNA-seq analysis

Twelve-day-old seedlings grown on half-strength MS plates were collected at 0 and 22 min after touching, snap-frozen in liquid nitrogen and stored at −80 ⁰C until use. Approximately 5 to 6 seedlings were sampled for one biological replicate and four biological replicates (*n* = 4) were used. Total RNA was isolated using the Spectrum™ Plant Total RNA Kit (Sigma Aldrich, STRN250-1KT, DNASE70-1set). Subsequently, total RNA was treated with Ambion Turbo DNase (Thermo Fisher Scientific, AM1907) and quantified by the Qubit RNA BR Assay Kit (Invitrogen, Q10210). 500 ng of RNA was used as the template for library preparation using Illumina TruSeq mRNA (poly-A selection) and TruSeq RNA UD Indexes for up to 96 samples (Illumina, 20022371). Then, sequencing was done using pooled samples on a half Illumina NovaSeq6000 S4 lane, 2 × 150 bp reads, incl Xp kit. Sequencing data were processed by using demultiplexing and quality controlled with FastQC^64^. Read alignment was mapped against the TAIR10 annotation using STAR^65^. About 32 million reads/sample were obtained on average. Counts were given to genes using featureCounts^66^, and analysis of DEGs was carried out using DeSEQ2.

In WT, genes were considered significantly responding to touch after 22 min if WT 0 min versus WT 22 min fold change (FC) > 1.5x (FC > 1.5 or FC < 0.6666) and p_*adj*_ < 0.05 (Supplementary Dataset 1). “Core early touch response” genes were determined if WT 0 min versus WT 22 min, FC > 1.5x (FC > 1.5 or FC < 0.6666) and p_*adj*_ < 0.05 using RNA-seq datasets from Van Moerkercke et al.^20^, Darwish et al.^19^ and this study (Supplementary Data 2). Under untouched conditions, genes were considered expressed differentially in the *camta1/2/3* and *mkk4/5* mutants compared to WT if WT 0 min versus mutant 0 min FC > 1.5x (FC > 1.5 or FC < 0.6666) and p_*adj*_ < 0.05 (Supplementary Data 3). Under touched condition, genes were considered expressed differentially in the *camta1/2/3* and *mkk4/5* mutants compared to WT if WT 22 min versus mutant 22 min FC > 1.5x (FC > 1.5 or FC < 0.6666) and p_*adj*_ < 0.05 (Supplementary Data 4). Genes were considered expressed differentially in response to water spray in the *myc2/3/4* mutant compared to WT if WT 25 min versus mutant 25 min FC > 1.5x (FC > 1.5 or FC < 0.6666) and p_*adj*_ < 0.05 (Supplementary Data 7).

GO analysis of CAMTA1/2/3- and MKK4/5- touch-induced genes was done using GOrilla^67^. GO analysis of 215 touch genes not regulated by either CAMTA1/2/3, MKK4/5 and MYC2/3/4 was done using PANTHER^68^. Both GOrilla and PANTHER used Fisher’s exact test for statistical analysis, and *p*-values were corrected for multiple testing using the FDR method. GO analyses were represented as dot plots using R (enriched GO terms with p_*adj*_ < 0.05).

### Protein extraction and phosphopeptide enrichment

For the phosphoproteome data set, 14-day-old plants were gently touched with a paint brush for 25 s. Two full 12 × 12 cm^2^ plates (about 100 seedlings/plate) were sampled and pooled for one biological replicate, and five biological replicates (*n* = 5) were used for each “genotype” × “time of treatment combination”. After the corresponding time points, samples were quickly harvested and immediately frozen in liquid nitrogen. Tissues were reduced to a fine powder by manually grinding with a mortar and pestle. About 1 mL of tissue powder was resuspended with 5 mL homogenization buffer (30% sucrose, 50 mM Tris-HCl, pH 8, 0.1 M KCl, 5 mM EDTA, 500 mM DTT, 2 phosphatase and 1 protease inhibitor tablet). Samples were sonicated for a total of 30 s and centrifuged at full speed for 15 min at 4 °C. The supernatant was collected and then the following solvents were sequentially added without mixing in between: (a) 3 parts of methanol, (b) 1 part of chloroform (5 mL) and (c) 4 parts of H_2_O (20 mL). Samples were vortexed and centrifuged at full speed and room temperature for 15 min. The upper aqueous phase was discarded, and 4 parts of methanol were added to interface and lower phase remaining in each tube. Samples were mixed and centrifuged at full speed and room temperature for 10 min. The supernatant was discarded and the pellet was washed using 80 % acetone. Samples were centrifuged at full speed and room temperature for 10 min. The supernatant was discarded and the pellet was dried in the air for 1 h. Samples were solubilized in 0.5 mL of 8 M urea in 50 mM TEAB. For S-reduction and S-alkylation, samples were incubated for 15 min at 30 °C in the dark with 15 mM TCEP-HCl, pH 8, 30 mM iodoacetamide. 3 mg of protein (determined with a nanodrop) was diluted in 8x TEAB 50 mM and pre-digested with 0.18 AU EndoLysC (SignalChem) at 37 °C while mixing, 2 to 4 h in the dark. Then, 10 μg of trypsin (Promega) per mg protein was added, and the reaction was incubated for 17 h at 37 °C with agitation. To arrest digestion, TFA (≥4 μL/1 mL, pH ≤  3) was added to a final concentration of 0.2%. Samples were desalted with 5 mL of C18 SPE cartridges.

For the phosphopeptide enrichment, MagReSyn® Ti-IMAC (MR-TIM002) hyper-porous magnetic microspheres (ReSyn Biosciences) were used. Briefly, the dried protein-digested pellet was resuspended in 500 μL of loading buffer and mixed with the equilibrated microspheres. Samples were incubated for 20 min at room temperature with continuous mixing. Unbonded and unspecific peptides were removed by a series of washes with loading (1 time) and washing (3 times) buffers. Between each washing step, samples were placed in a magnetic separator to clear the microspheres from the solution and the supernatant was discarded. Bound peptides were eluted from the microspheres by incubating twice with 80 μL of elution buffer for 15 min with agitation (600 rpm). Eluates were acidified by adding 6 μL of 100% formic acid. Finally, samples were dried in a Speedvac.

### LC-MS/MS analysis

Each sample was analyzed by LC-MS/MS on an Ultimate 3000 RSLC nano LC (Thermo Fisher Scientific, Bremen, Germany) in-line connected to a Q Exactive mass spectrometer (Thermo Fisher Scientific). The peptides were first loaded on a trapping column [made in-house, 100 μm internal diameter (ID) × 20 mm, 5 μm beads C18 Reprosil-HD, Dr. Maisch, Ammerbuch-Entringen, Germany]. After flushing the trapping column, peptides were loaded in solvent A (0.1% formic acid in water) on a reverse-phase column (made in-house, 75 µm ID × 250 mm, 1.9 µm Reprosil-Pur-basic-C18-HD beads, Dr Maisch, packed in the needle) and eluted by an increase in solvent B (0.1% formic acid in acetonitrile) using a linear gradient from 2% solvent B to 55% solvent B in 120 min, followed by a washing step with 99% solvent B, all at a constant flow rate of 300 nl min^–1^. The mass spectrometer was operated in data-dependent, positive ionization mode, automatically switching between MS and MS/MS acquisition for the five most abundant peaks in each MS spectrum. The source voltage was set at 4.1 kV and the capillary temperature at 275 °C. One MS1 scan (*m*/*z* 400−2000, AGC target 3 × 10^6^ ions, maximum ion injection time 80 ms), acquired at a resolution of 70,000 (at 200 m/z), was followed by five tandem MS scans (resolution 17,500 at 200 m/z) of the most intense ions fulfilling pre-defined selection criteria (AGC target 5 × 10^4^ ions, maximum ion injection time 80 ms, isolation window 2 Da, fixed first mass 140 m/z, spectrum data type: centroid, under-fill ratio 2%, intensity threshold 1.3 × E4, exclusion of unassigned, 1, 5–8, >8 positively charged precursors, peptide match preferred, exclude isotopes on, dynamic exclusion time 12 s). The HCD collision energy was set to 25% normalized collision energy and the polydimethylcyclosiloxane background ion at 445.120025 Da was used for internal calibration (lock mass).

### Phosphoproteomic database searching

MS/MS spectra were searched against the Araport11 database for *Arabidopsis thaliana* with MaxQuant software (version 2.1.4.0), which was carried out using the high-performance computing facilities of the University of Ghent (https://www.ugent.be/hpc/en/infrastructure), a program package allowing MS1-based label-free quantification acquired from Orbitrap instruments^69,70^. The precursor mass tolerance was set to 20 ppm for the first search (used for nonlinear mass re-calibration) and to 4.5 ppm for the main search. Trypsin was used as the enzyme setting. Cleavages between lysine/arginine-proline residues and up to two missed cleavages were allowed. S-Carbamidomethylation of cysteine residues was selected as a fixed modification, and oxidation of methionine residues was selected as a variable modification. The false discovery rate for peptide and protein identification was set to 1% and the minimum peptide length was set to 7. The minimum score threshold for both the modified and unmodified peptides was set to 30. The MaxLFQ algorithm allowing for label-free quantification^70^ and the “matching between runs” feature were enabled. To calculate the protein ratios, both unique and razor peptides (non-unique peptides assigned to a protein group with the largest number of identified peptides) were selected.

### Phosphoproteomic data analysis

For quality control and data analysis, we used a custom Python script executed in a Jupyter notebook^71^. Python libraries used included Pandas^72^, Numpy^73^, and Seaborn^74^. Our files from MaxQuant were loaded and data were filtered as follows: Phosphorylated sited identified as reverse, potential contaminants or with localization probability less than 75% were removed. Intensity values were transformed using variance-normalizing stabilization (VSN), using the package “justvsn” in R^75^. Batch effect was corrected using ComBat^76^ implemented for proteomics in the HarmonizR package from R^77^, coupled with a prior imputation step using the Random Forest Decision Tree^78^ and MissForest module^79^ with 100 iterations. The resulting data was used for visualization and statistical analysis.

Uniform Manifold Approximation and Projection (UMAP) dimension reduction was plotted using the Python UMAP package^80^ with the following parameters: number of neighbors = 10, minimum distance = 0.3 and metric = Euclidean.

The “genotype” effect on protein phosphorylation in mutants vs WT was determined if FDR_genotype < 0.05 in “mat1”, then in “mat2” p_COMP1 < 0.05 for WT versus *aos*, p_COMP2 < 0.05 for WT versus *mkk4/5*, 1.5x FC (FC > 1.5 or < 0.6666) (Supplementary Data 11). The “time” effect on protein phosphorylation at the time points 1, 3 and 10 min (touched) vs 0 min (untouched) in WT was determined if FDR_TIME < 0.05 in “mat1”, then in “mat2” p_COMP7 < 0.05 and COMP7 > 1.5 or < 0.6666 for 1 min versus 0 min, p_COMP8 < 0.05 and COMP8 > 1.5 or < 0.6666 for 3 min versus 0 min, p_COMP9 < 0.05 and COMP9 > 1.5 or < 0.6666 for 10 min versus 0 min (Supplementary Data 12). The “interaction” effect (genotype x time) on protein phosphorylation in mutants vs WT was determined if FDR_int < 0.05 in “mat1”, then in “mat2” p_COMP1_time for *aos* versus WT, p_COMP2_time < 0.05 for *mkk4/5* versus WT (Supplementary Data 15). Average linkage hierarchical clustering with Pearson correlation and *k*-means clustering were done using the multiple experiment viewer (MeV) software. GO analysis of differentially phosphorylated sites in each cluster was done using Gorilla^67^ (enriched GO terms with p < 0.05 and *p* < 0.05 for “time” and “interaction” effect, respectively) (Supplementary Data 13, 14, 16 and 17).

To extract phosphorylation motifs, the touch-regulated phosphopeptides were submitted to “Modification Motifs” (MoMo) online motif search tool (https://meme-suite.org/meme/tools/momo)^40^. Parameters set for the algorithm, width, minimum number of occurrences, *p*-value threshold and background were motif-x, 31, 20, 10^−6^ and the Ensembl Plants Genomes and Proteins - *Arabidopsis thaliana* (version 57), respectively. Identified phosphorylation motifs by MoMo were illustrated using Weblogo^81^.

### Immunoblotting analysis

Twelve-day-old seedlings grown on half-strength MS plates were subjected to touch and wounding treatment. Touch treatment was performed as described above. Wounding treatment was performed by squeezing rosette leaves using tweezers as previously described^33^. Approximately 5–6 seedlings were sampled for one biological replicate and three biological replicates (*n* = 3) were used for each mechanical treatment for 0, 1, 3, 10 and 20 min samples of Col-0, *mkk4/5*, *mapkkk3-2/5-2* and *mapkkk3-2/5-2 yda-∆42*. After the treatments, plant samples were snap-frozen in liquid nitrogen and stored at −80 ⁰C until use. Total protein isolation was performed as previously described^26^. Briefly, ~300 µl of protein isolation buffer (50 mM Tris-HCl pH 7.5, 150 mM NaCl, 2 mM DTT, 2.5 mM NaF, 1.5 mM Na_3_VO_4_, 0.5% (w/v) Nonidet P-40, 50 mM β-glycerophosphate, and proteinase inhibitor cocktail) was added to ~500 mg of frozen ground samples. The samples were then vortexed until thawed, centrifuged once at 6000 *g* for 20 min at 4 °C, and twice at 17,000 *g* for 10 min at 4 °C. The supernatant was collected, and the protein concentration was determined by Bradford assays (BioRad). Isolated proteins (50 µg) were separated by SDS-PAGE and transferred to a PVDF membrane using the Trans-Blot Turbo Mini PVDF Transfer Pack (BioRad) and the Trans-Blot Turbo Transfer System (BioRad). Blots were probed with the primary antibody: anti-phospho-p44/42 MAPK polyclonal antibody (Cat#9101; Cell Signaling Technology, MA, USA) (1:1000 dilution). Anti-rabbit antibody conjugated with horseradish peroxidase (HRP) (A9169, Sigma Aldrich) (1:10,000 dilution) was used as the secondary antibody. Chemiluminescence was detected using Clarity Western ECL Substrate (BioRad) and the ChemiDoc XRS+ System (BioRad).

### Thigmomorphogenesis analysis

Seeds were sown in the soil mixture (soil, perlite, and vermiculate 4:1:1) followed by stratification at 4 °C in the dark for 2 days prior to transferring to the growth chamber. Plants were grown under long-day conditions (16 h light/8 h dark, approximately 120 µmol photons m^−2^ s^−1^). Touch treatment was performed when plants were 2 weeks old. Plants were gently brushed twice per day for 10 times using a soft paint brush. Touch treatment was carried out on day 14 until all plants had bolted depending on the genotypes. Plants were recognized to have bolted once the inflorescence stock had reached 1 cm height, and percentage of bolting was determined as previously described^25^. The rosette leaf size of 28-day-old plants was measured using ImageJ. Statistical significance was based on Student’s *t* test.

### Reporting summary

Further information on research design is available in the Nature Portfolio Reporting Summary linked to this article.

## Supplementary information

Supplementary Information

Description of Additional Supplementary Information

Supplementary Data 1

Supplementary Data 2

Supplementary Data 3

Supplementary Data 4

Supplementary Data 5

Supplementary Data 6

Supplementary Data 7

Supplementary Data 8

Supplementary Data 9

Supplementary Data 10

Supplementary Data 11

Supplementary Data 12

Supplementary Data 13

Supplementary Data 14

Supplementary Data 15

Supplementary Data 16

Supplementary Data 17

Supplementary Data 18

Supplementary Data 19

Reporting Summary

Transparent Peer Review file

## Source data

Source Data

## Data Availability

The mass spectrometry proteomics data generated in this study have been deposited in the ProteomeXchange Consortium via the PRIDE^82^ partner repository under accession code PXD060728. The raw RNA-seq data generated in this study have been deposited in the ArrayExpress database under accession number E-MTAB-15045. Source data are provided with this paper.
