# Supplementary material for: A sequential MAP kinase cascade regulates mechanical signalling
Source: Nat Commun. 2026 Jul 1;17:5729. doi: 10.1038/s41467-026-74994-x (PMC13324856; doi:10.1038/s41467-026-74994-x)
Supplement: Supplementary file 1 — Supplementary Information [file 41467_2026_74994_MOESM1_ESM.pdf]

A sequential MAP kinase cascade regulates mechanical signalling

Huy Cuong Tran[1], Essam Darwish[1, 2], Viktor Johansson[1], Guadalupe Fernandez-Milmanda[3, 4], Tingting Zhu[3, 4], Cássio Flávio Fonseca de Lima[3, 4], Brigitte Van De Cotte[3, 4], Jean Colcombet[5], Marnik Vuylsteke[3, 4], Ive De Smet[3, 4], Alain Goossens[3, 4], Olivier Van Aken[1*]

[1] Department of Biology, Lund University, Lund, Sweden

[2] Plant Physiology Section, Agricultural Botany Department, Faculty of Agriculture, Cairo University, Egypt.

[3] Ghent University, Department of Plant Biotechnology and Bioinformatics, B-9052 Ghent, Belgium

[4] VIB Center for Plant Systems Biology, B-9052 Ghent, Belgium

[5] Université Paris-Saclay, CNRS, INRAE, Institute of Plant Sciences Paris-Saclay (IPS2), 91190 Gif sur Yvette, France

*Corresponding author

Olivier Van Aken
Molecular Cell Biology Unit
Department of Biology
Lund University
Sölvegatan 35
Lund 223 62 – Lund, Sweden
Tel: +46 76 210 14 03
E-mail: olivier.van_aken@biol.lu.se

SUPPLEMENTARY FIGURES

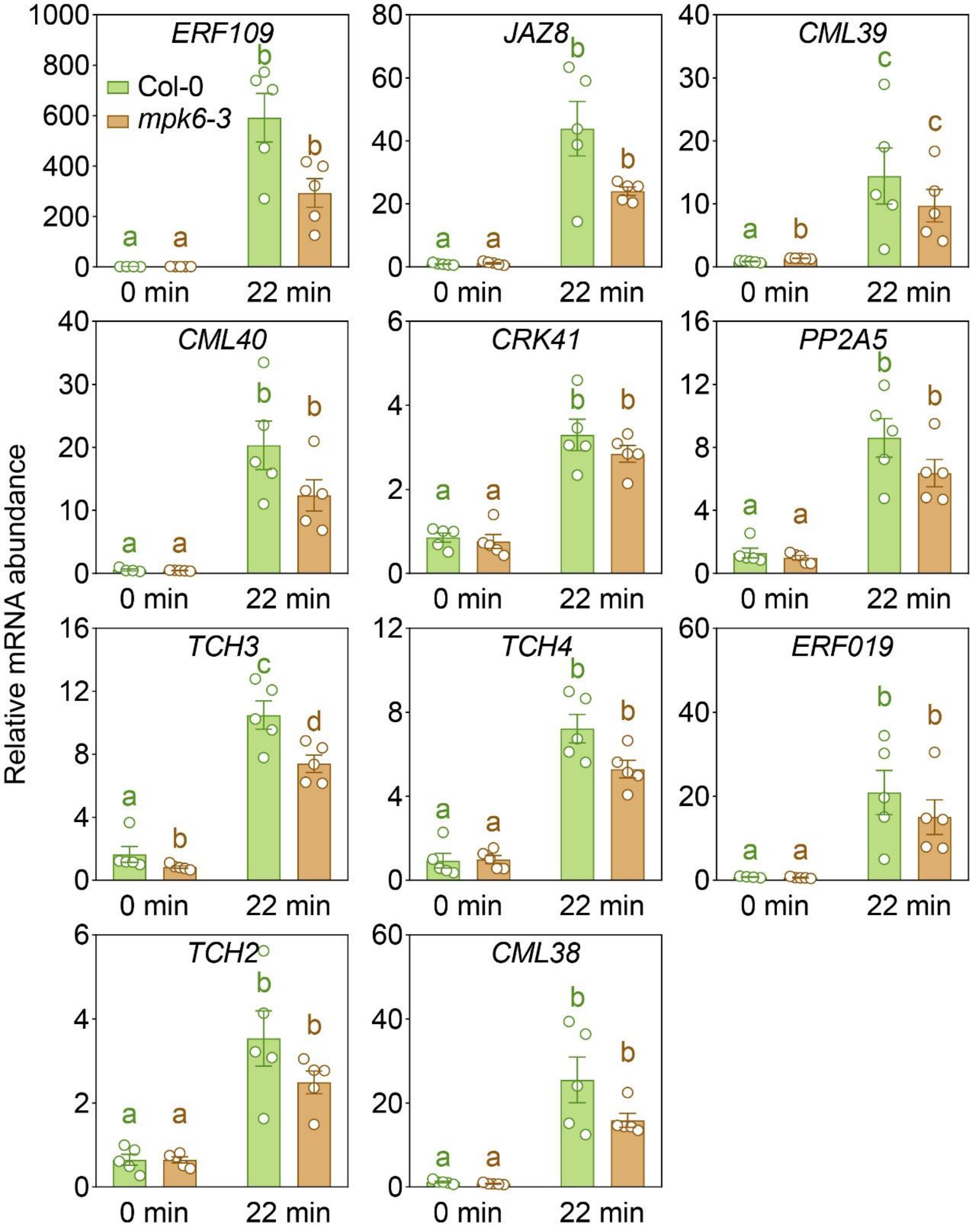

Supplementary Fig. 1 Analysis of touch marker gene expression in the *mpk6-3* mutant. 12-day-old seedlings before (0 min) and 22 min after touching by gentle brushing were collected for qRT-PCR. The expression of selected touch marker genes was measured in Col-0 (WT) versus the *mpk6-3* mutant. The y axis represents the relative mRNA level. Data are presented as mean ±SE. n=5 biologically independent samples. Each dot indicates a biological replicate. Statistical significance was based on Kruskal-Wallis test followed by Wilcoxon rank sum tests. Different letters represent the significant differences between genotypes ($p < 0.05$).

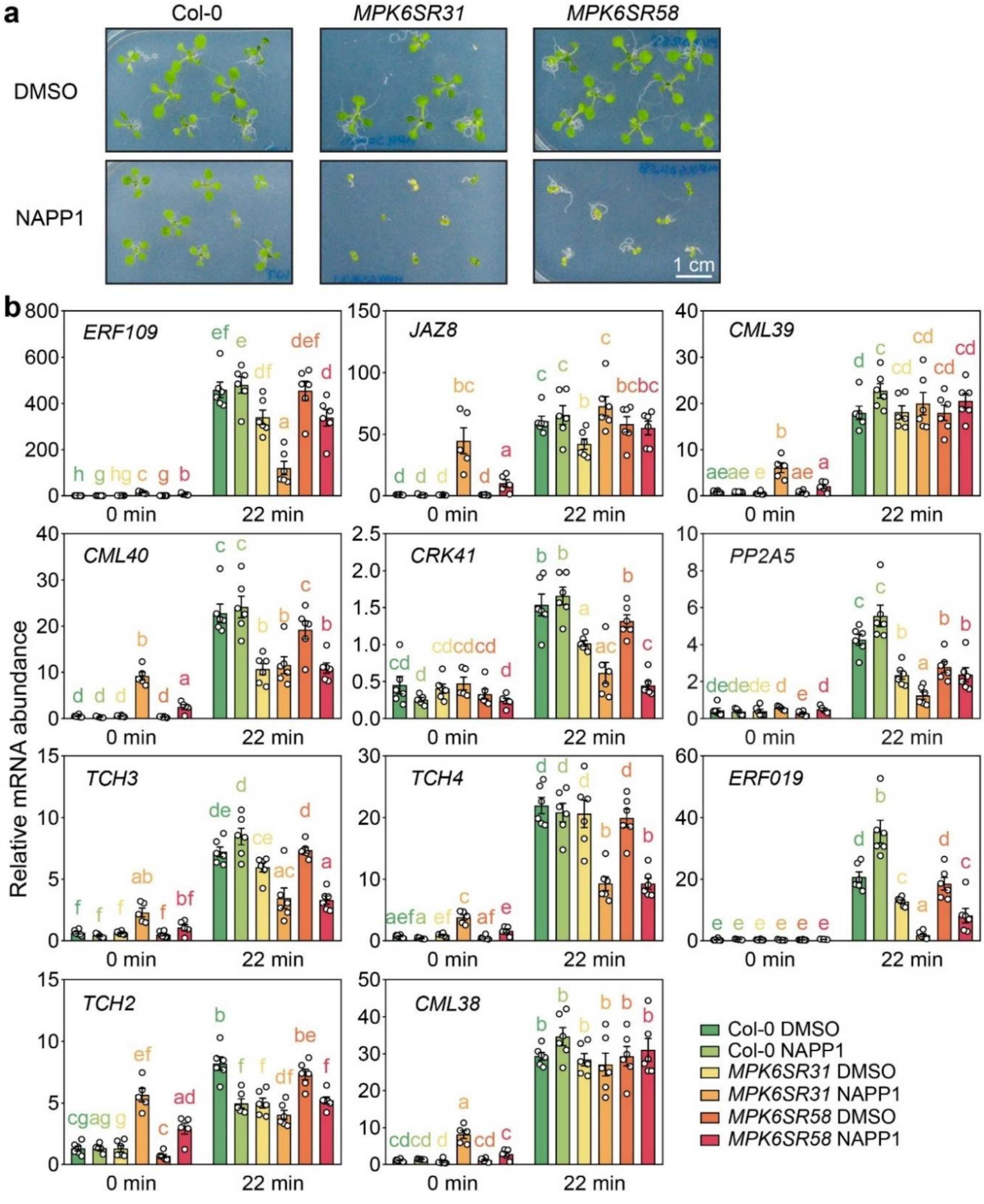

Supplementary Fig. 2 Analysis of touch marker gene expression in the *MPK6SR* lines. **a** A representative picture of 10-day-old WT and *MPK6SR* seedlings grown on half-strength MS containing either DMSO (mock) or 1 µM NAPP1. Scale bar = 1 cm. **b** Touch marker gene expression in the *MPK6SR* lines. 12-day-old seedlings before (0 min) and 22 min after touching by gentle brushing were collected for qRT-PCR. The expression of selected touch marker genes was measured in Col-0 (WT) versus the *MPK6SR* lines (*MPK6SR31* and *MPK6SR58*). The y axis represents the relative mRNA level. Data are presented as mean ±SE. n=6 biologically independent samples. Each dot indicates a biological replicate. Statistical significance was based on Kruskal-Wallis test followed by Wilcoxon rank sum tests. Different letters represent the significant differences between genotypes ($p < 0.05$).

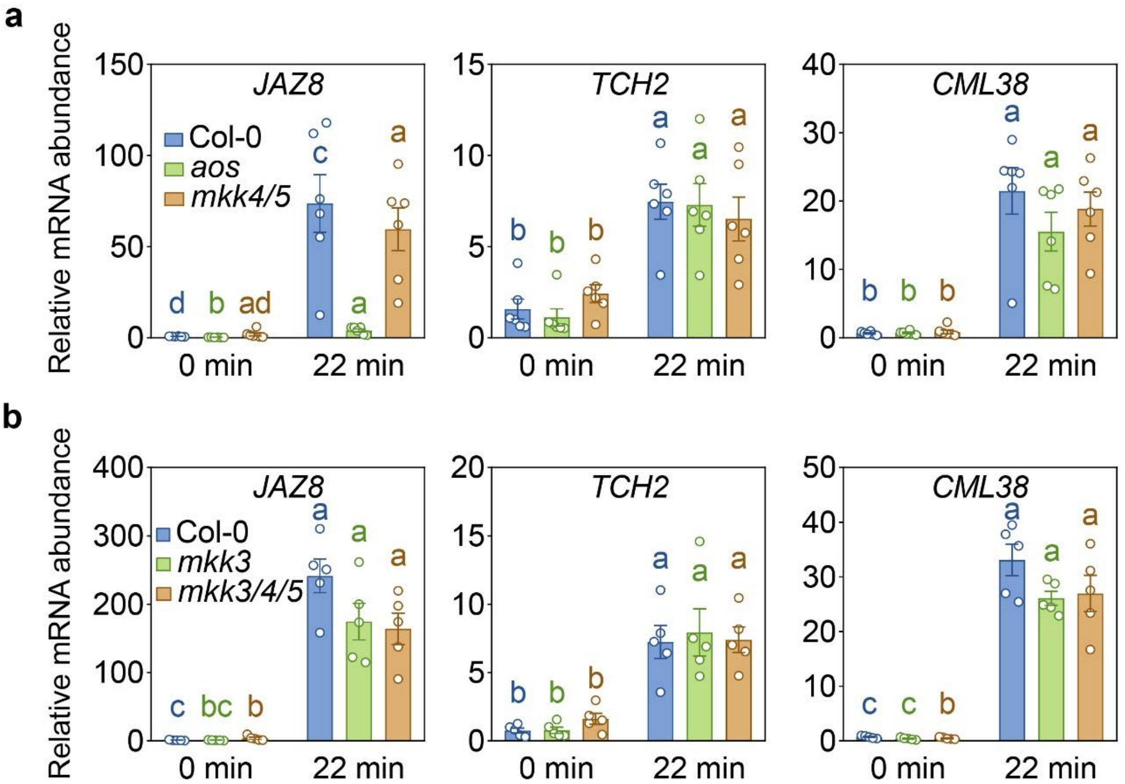

Supplementary Fig. 3 Analysis of touch marker gene expression in the mutants *aos*, *mkk4/5*, *mkk3* and *mkk3/4/5*. 12-day-old seedlings before (0 min) and 22 min after touching by gentle brushing were collected for qRT-PCR. The expression of selected touch marker genes was measured in Col-0 (WT) versus the *aos* and *mkk4/5* mutants (**a**), and WT versus the *mkk3* and *mkk3/4/5* mutants (**b**). The y axis represents the relative mRNA level. Data are presented as mean ±SE. n=6 (**a**) and n=5 (**b**) biologically independent samples. Each dot indicates a biological replicate. Statistical significance was based on Kruskal-Wallis test followed by Wilcoxon rank sum tests. Different letters represent the significant differences between genotypes ($p < 0.05$).

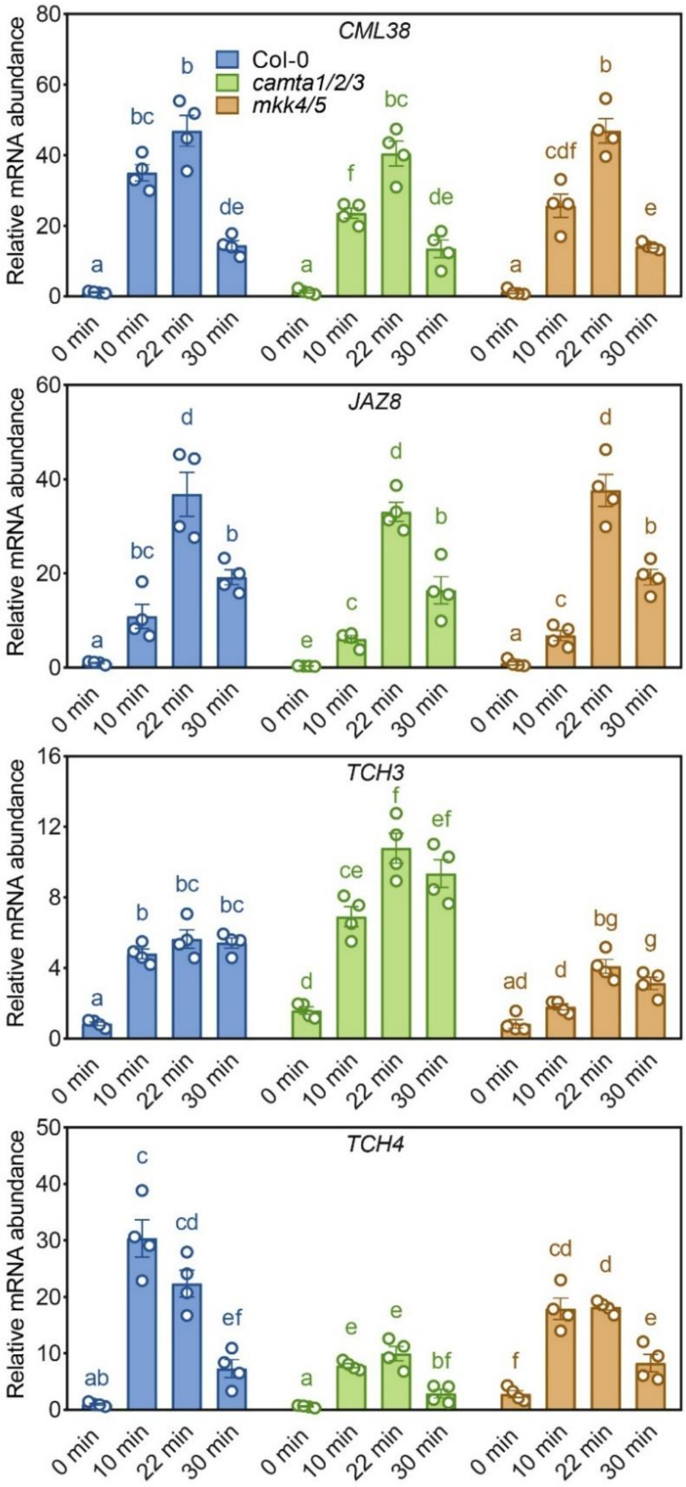

Supplementary Fig. 4 Analysis of touch marker gene expression at different time points in the mutants *camta1/2/3* and *mkk4/5*. 12-day-old seedlings before (0 min) and 10, 22 and 30 min after touching by gentle brushing were collected for qRT-PCR. The expression of selected touch marker genes was measured in Col-0 (WT) versus *camta1/2/3* and *mkk4/5*. The y axis represents the relative mRNA level. Data are presented as mean ±SE. n=4 biologically independent samples. Each dot indicates a biological replicate. Statistical significance was based on Kruskal-Wallis test followed by Wilcoxon rank sum tests. Different letters represent the significant differences between genotypes ($p < 0.05$).

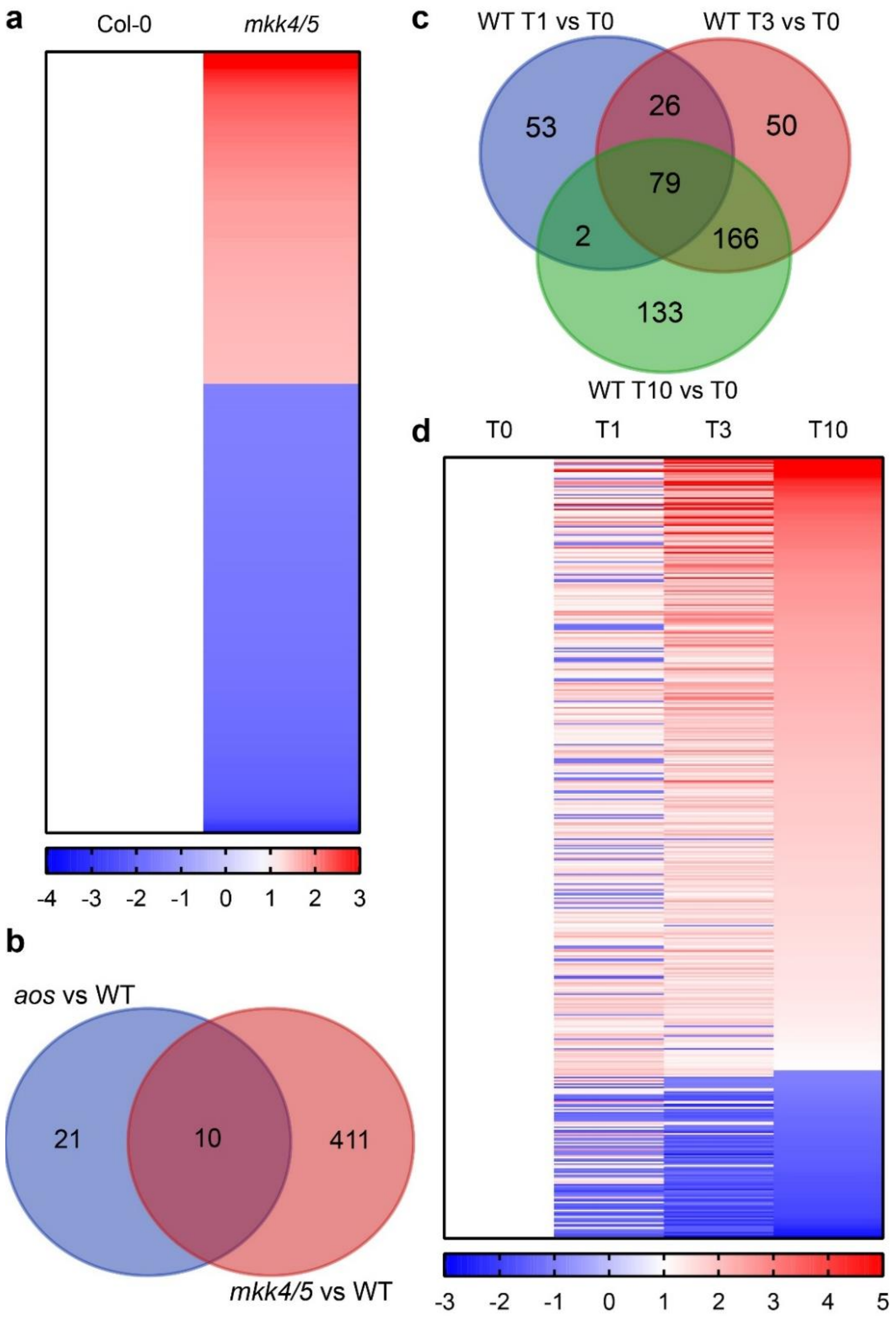

Supplementary Fig. 5 Dynamic changes in protein phosphorylation due to genotype effect and time effect. a Heatmap represents all the differentially phosphorylated sites due to genotype effect in *mkk4/5* compared to in Col-0. Color bar indicates linear fold changes. **b** Venn diagram represents the number of phosphosites due to genotype effect between the *aos* and *mkk4/5* mutants (FDR_genotype < 0.05, p_COMP1 < 0.05 for WT versus *aos*, p_COMP2 < 0.05 for WT versus *mkk4/5*, 1.5x FC (FC > 1.5 or <0.6666)). **c** Venn diagram representing the number of differentially phosphorylated sites due to time effect in WT at 1, 3 and 10 min after touching compared to untouched condition. **d** Heatmap represents all the differentially phosphorylated sites due to time effect in WT at 1, 3 and 10 min after touching compared to untouched condition. Color bar indicates linear fold changes.

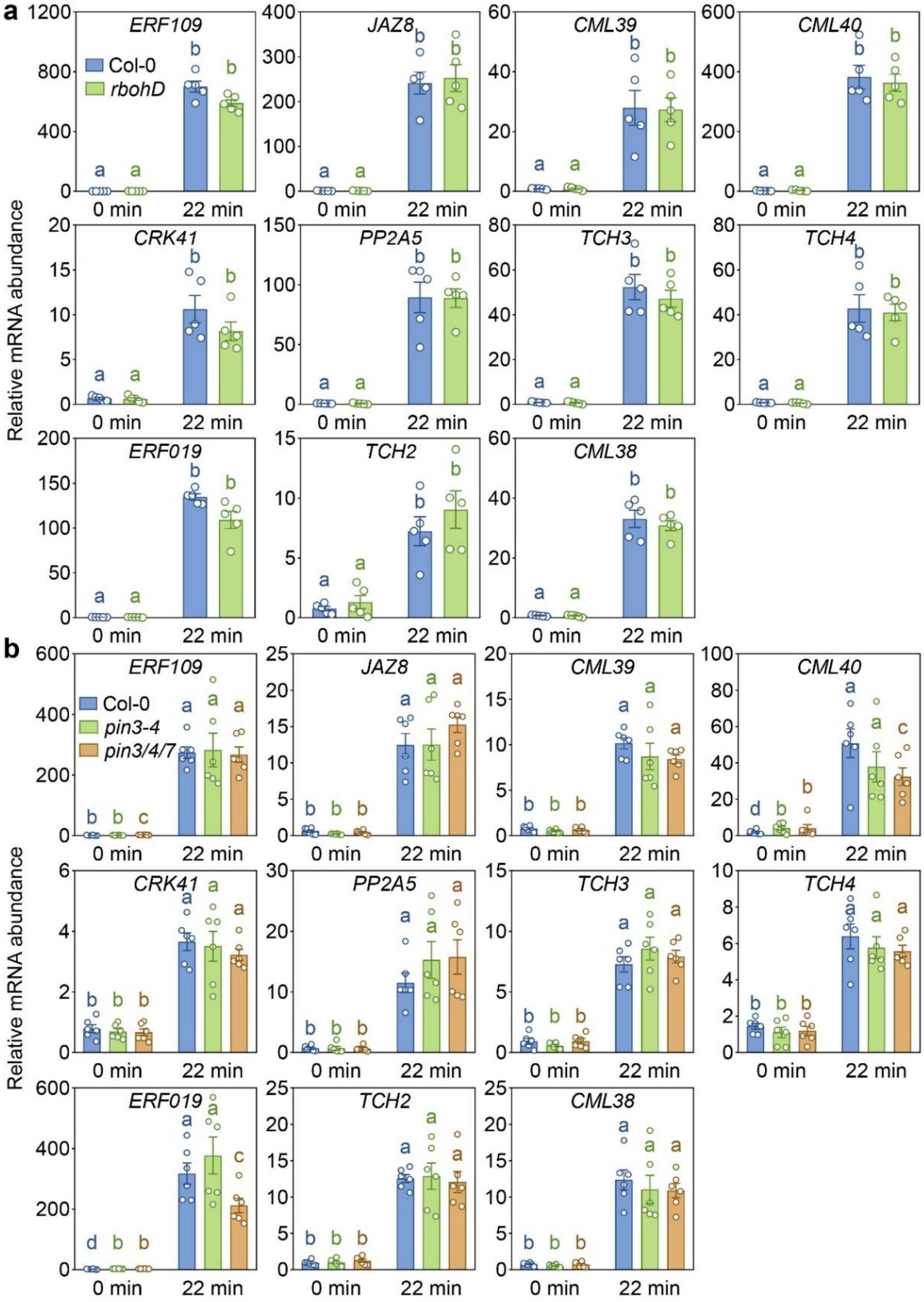

Supplementary Fig. 6 Analysis of touch marker gene expression in the *rbohD*, *pin3-4* and *pin3/4/7* mutants. 12-day-old seedlings before (0 min) and 22 min after touching by gentle brushing were collected for qRT-PCR. The expression of selected touch marker genes was measured in Col-0 (WT) versus the mutants *rbohD* (**a**) and *pin3-4* and *pin3/4/7* (**b**). The y axis represents the relative mRNA level. Data are presented as mean ±SE. n=5 (**a**) and n=6 (**b**) biologically independent samples. Each dot indicates a biological replicate. Statistical significance was based on Kruskal-Wallis test followed by Wilcoxon rank sum tests. Different letters represent the significant differences between genotypes ($p < 0.05$).

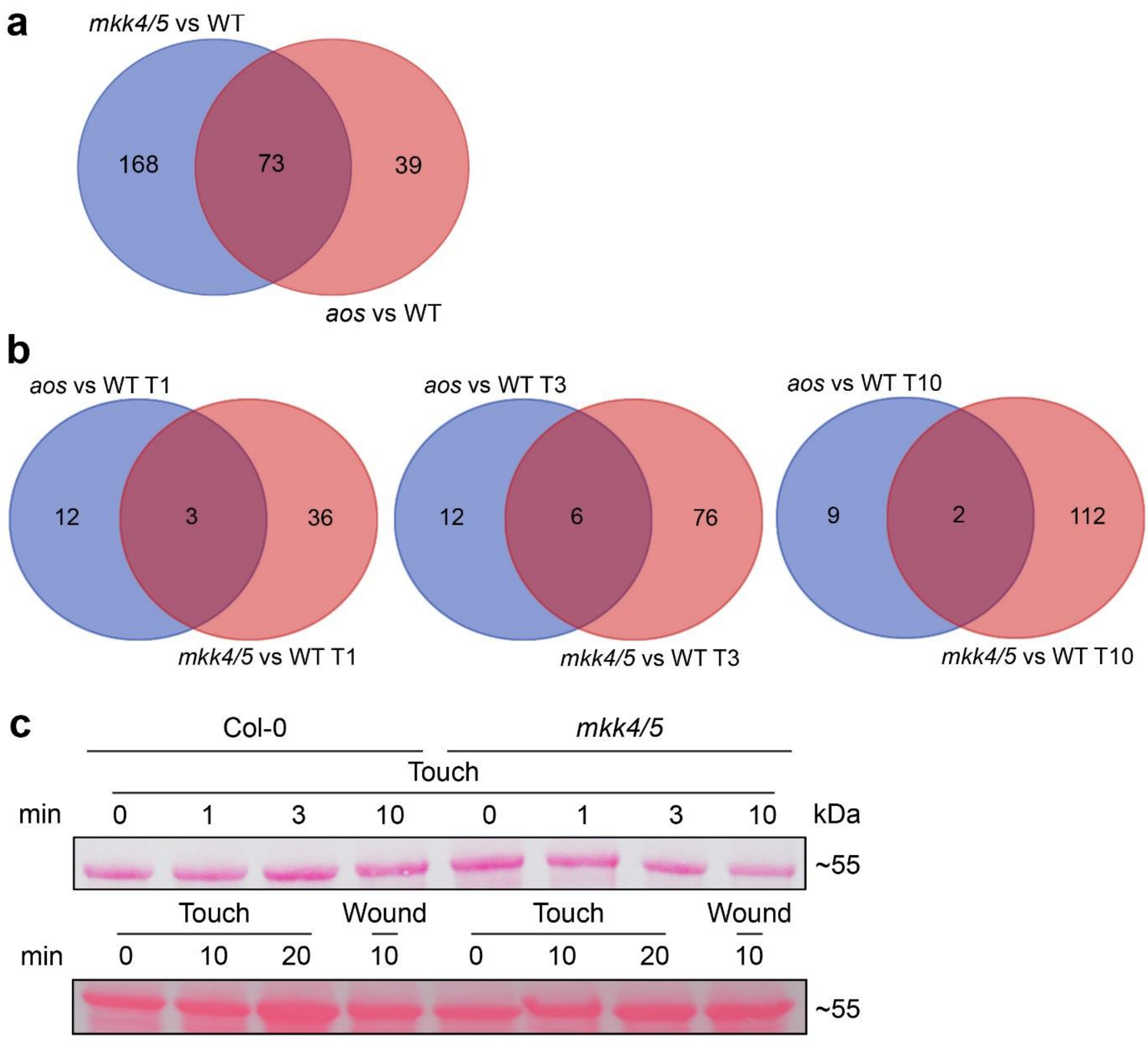

Supplementary Fig. 7 Comparison of protein phosphosites due to interaction effect between the *aos* and *mkk4/5* mutants and immunoblot analysis of phosphorylated MPKs in *mkk4/5* before and after touch or wounding treatment. a Venn diagram represents the number of phosphosites due to interaction effect over the time course of touch treatment between the *aos* and *mkk4/5* mutants (FDR_int < 0.05, p_COMP1_time for *aos* versus WT, p_COMP2_time <0.05 for *mkk4/5* versus WT). **b** Venn diagram represents the number of phosphosites due to interaction effect at each time point of touch treatment (1, 3 and 10 min) between the *aos* and *mkk4/5* mutants (FDR_int < 0.05, p_COMP1_time for *aos* versus WT, p_COMP2_time <0.05 for *mkk4/5* versus WT). A cutoff in FC was applied for each time point as followed: *aos* T1/WT T1 >1.5 or <0.66666 and *mkk4/5* T1/WT T1 >1.5 or < 0.66666; *aos* T3/WT T3 >1.5 or <0.66666 and *mkk4/5* T3/WT T3 >1.5 or < 0.66666; *aos* T10/WT T10 >1.5 or <0.66666 and *mkk4/5* T10/WT T10 >1.5 or < 0.66666. **c** Ponceau staining of immunoblots using an antibody against phosphorylated MAPKs in WT and *mkk4/5* before and after touch or wounding treatment. Similar results were obtained in three independent experiments.

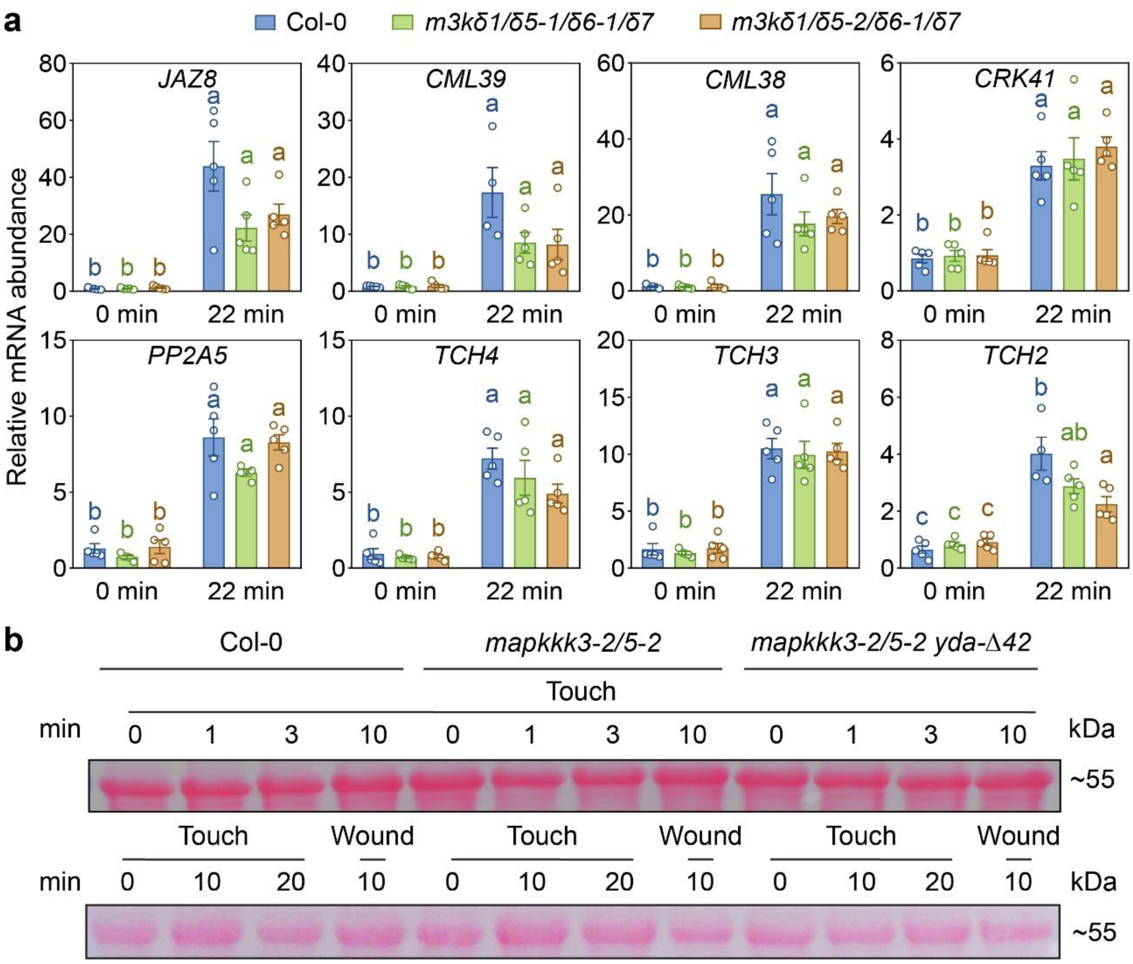

Supplementary Fig 8 Analysis of touch marker gene expression in the *m3kδ1/δ5-1/δ6-1/δ7* and *m3kδ1/δ5-2/δ6-1/δ7* mutants and immunoblot analysis of phosphorylated MPKs in *mapkkk3-2/5-2 and mapkkk3-2/5-2 yda-Δ42* before and after touch or wounding treatment. a 12-day-old seedlings before (0 min) and 22 min after touching by gentle brushing were collected for qRT-PCR. The expression of selected touch marker genes was measured in Col-0 (WT) versus the *m3kδ1/δ5-1/δ6-1/δ7* and *m3kδ1/δ5-2/δ6-1/δ7* mutants. The y axis represents the relative mRNA level. Data are presented as mean ±SE. n=5 biologically independent samples. Each dot indicates a biological replicate. Statistical significance was based on Kruskal-Wallis test followed by Wilcoxon rank sum tests. Different letters represent the significant differences between genotypes ($p < 0.05$). **b** Ponceau staining of immunoblots using an antibody against phosphorylated MAPKs on WT, *mapkkk3-2/5-2* and *mapkkk3-2/5-2 yda-Δ42* mutants before and after touch or wounding treatment. Similar results were obtained in three independent experiments.

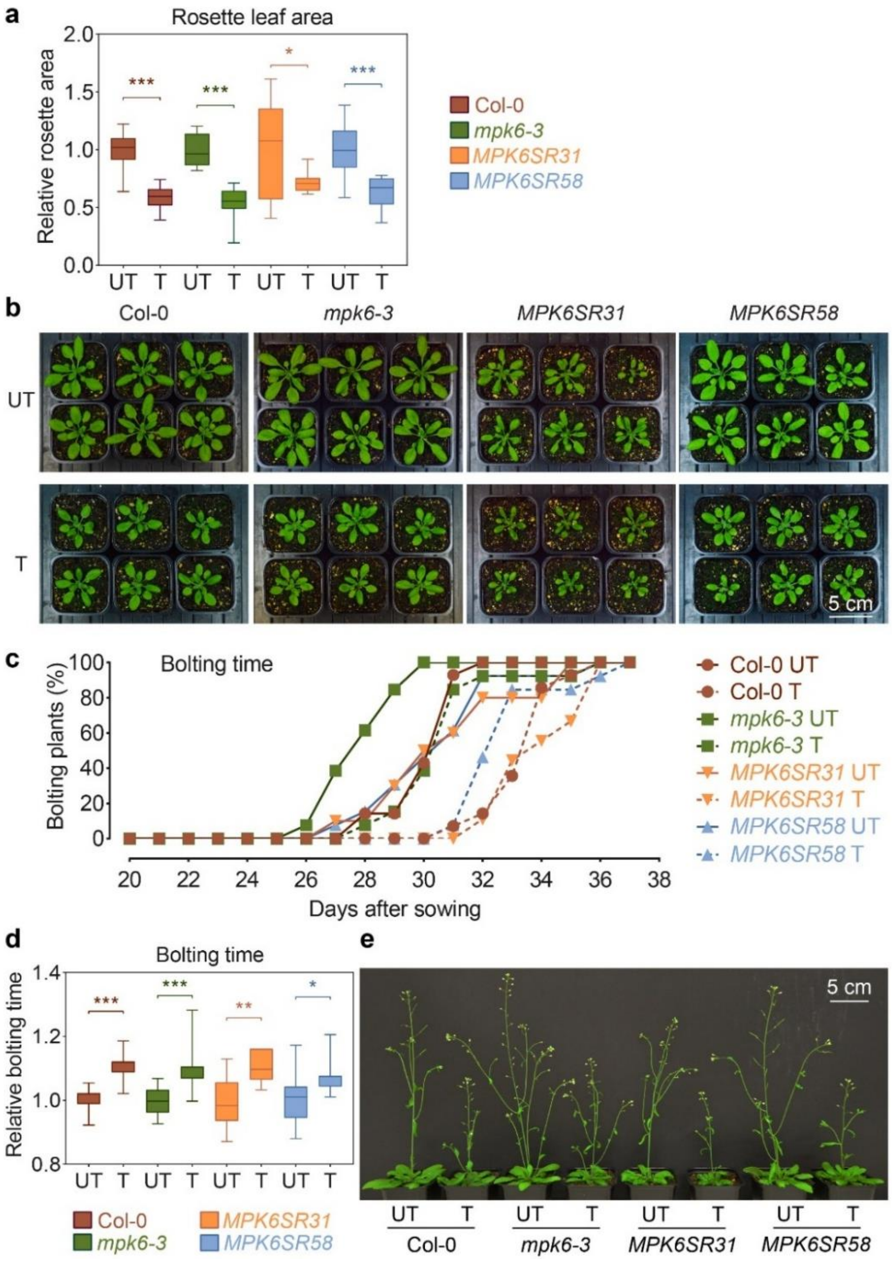

Supplementary Fig. 9 Analysis of the *mpk6-3* mutant and *MPK6SR* lines on thigmomorphogenesis. a Box and whisker plot representing relative rosette leaf area of 28-day-old plants, either untouched or after 14 days of regular touching. n=9-14 plants (*=p<0.05, ***=p<0.001, ****=p<0.0001). Box plot shows the minimum and maximum values (whiskers), the 25th and 75th percentiles (box bounds), and the median (center line). **b** A representative picture of 28-day-old untouched plants or plants in which touch treatment had been applied twice-daily for 14 days. Scale bar = 5 cm. **c** Line graph showing the percentage of bolting plants over the growth period (days after sowing). **d** Box and whisker plot represents relative bolting time under untouched and touched conditions. n=9-14 plants (*=p<0.05, ***=p<0.001, ****=p<0.0001). Box plot shows the minimum and maximum values (whiskers), the 25th and 75th percentiles (box bounds), and the median (center line). **e** A representative picture of 38-day-old untouched plants or plants in which touch treatment had been applied twice-daily for 24 days. Scale bar = 5 cm. UT=untouched, T=touched.
