# Supplementary material for: A sequential MAP kinase cascade regulates mechanical signalling
Source: Nat Commun. 2026 Jul 1;17:5729. doi: 10.1038/s41467-026-74994-x (PMC13324856; doi:10.1038/s41467-026-74994-x)
Supplement: Supplementary file 2 — Description of Additional Supplementary Information [file 41467_2026_74994_MOESM2_ESM.pdf]

Description of Additional Supplementary Files

Supplementary Data 1. RNA-seq analysis of the touch response in the mutants *camta1/2/3* (**a**) and *mkk4/5* (**b**).

Supplementary Data 2. List of "core early touch response" genes.

Supplementary Data 3. Genes affected in the mutants *camta1/2/3* (**a**) and *mkk4/5* (**b**) under untouched condition.

Supplementary Data 4. Touch-responsive DEGs of the mutants *camta1/2/3* (**a**) and *mkk4/5* (**b**) at 22 min versus WT at 22 min.

Supplementary Data 5. Normalized expression values used for the heatmap of transcripts differentially expressed between Col-0 and the mutants *camta1/2/3* (**a**) and *mkk4/5* (**b**) under untouched and touched conditions.

Supplementary Data 6. GO analyses of CAMTA1/2/3- and MKK4/5-induced genes after touched.

Supplementary Data 7. DEGs in response to touch in the myc2/3/4 mutant (**a**) and identified MYC2 touch regulon (**b**)[1].

Supplementary Data 8. GO analysis of touch responsive genes that are likely not regulated by either MKK4/5, CAMTA1/2/3 or MYC2/3/4.

Supplementary Data 9. List of identified CAMTA1/2/3 touch regulon genes.

Supplementary Data 10. Phosphoproteomic analysis of WT, *aos* and *mkk4/5* subjected to touch treatment. (**a**) Non-imputed dataset. (**b**) Imputed dataset.

Supplementary Data 11. The "genotype" effect on protein phosphorylation in WT versus the mutants *aos* (**a**) and *mkk4/5* (**b**).

Supplementary Data 12. The "time" effect on protein phosphorylation in response to touch.

Supplementary Data 13. Cluster analysis of the proteins that have differentially phosphorylated sites in WT after touching.

Supplementary Data 14. GO analysis of clusters containing proteins that have differentially phosphorylated sites in WT after touching.

Supplementary Data 15. The "interaction" (genotype x time) effect on protein phosphorylation in WT versus the mutants *aos* (**a**) and *mkk4/5* (**b**).

Supplementary Data 16. Cluster analysis of the proteins that have differentially phosphorylated sites between genotypes at each time point after touching.

Supplementary Data 17. GO analysis of clusters containing proteins that have differentially phosphorylated sites between genotypes at each time point after touching.

Supplementary Data 18. Common touch-responsive phosphoproteins identified in this study and in the phosphoproteomic dataset reported by Wang et al.[2].

Supplementary Data 19. Oligonucleotide sequences used in this study.

References

1. Van Moerkercke, A., *et al.* A MYC2/MYC3/MYC4-dependent transcription factor network regulates water spray-responsive gene expression and jasmonate levels. *Proceedings of the National Academy of Sciences of the United States of America* **116**, 23345-23356 https://doi.org/10.1073/pnas.1911758116 (2019).
2. Wang, K., *et al.* Quantitative and functional posttranslational modification proteomics reveals that TREPH1 plays a role in plant touch-delayed bolting. *Proc Natl Acad Sci U S A* **115**, E10265-E10274 https://doi.org/10.1073/pnas.1814006115 (2018).
