# Supplementary material for: A sequential MAP kinase cascade regulates mechanical signalling
Source: Nat Commun. 2026 Jul 1;17:5729. doi: 10.1038/s41467-026-74994-x (PMC13324856; doi:10.1038/s41467-026-74994-x)
Supplement: Supplementary file 23 — Transparent Peer Review file [file 41467_2026_74994_MOESM23_ESM.pdf]

nature portfolio

Peer Review File

A sequential MAP kinase cascade regulates mechanical signalling

Corresponding Author: Professor Olivier Van Aken

This file contains all reviewer reports in order by version, followed by all author rebuttals in order by version.

Version 0:

Reviewer comments:

Reviewer #1

(Remarks to the Author)
The MAPKKK3/4/5-MKK4/5-MAPK3/6 module has been extensively studied (Sun et al., EMBO Rep. 2018; Liu et al., J Integr Plant Biol. 2022; Wang et al., Plant Physiol. 2022; Shao et al., Mol Plant. 2020), supporting its role in regulating various aspects of plant growth and development. In this manuscript, the authors explored the functions of MAPKKK3/4/5 and MKK4/5 in thigmomorphogenesis. Using transcriptomic, phosphoproteomic and molecular approaches, the authors expanded the known functions of the well-studied MAPK module, which plays an important role in early touch response. The manuscript is generally interesting; however, key additional experiments should be provided to support the authors' conclusions.

1. In their previous paper (Darwish et al., Sci. Adv. 2022), the camta3 mutant shows strong phenotypic defects in thigmomorphogenesis and flowering time. However, mutations in the MAPKKK3/4/5-MKK4/5 module genes exhibit much weaker phenotypic defects. Since the Arabidopsis genome has 20 MAPKs, 10 MAPKKs and 80 MAPKKKs (Kumar et al., J. Plant Biochem. Biotechnol. 2020), redundant functions likely exist.
2. In order to demonstrate the role of MAPK3/6 in thigmomorphogenesis, phenotypic analysis of the mpk6-3 mutant and MPK6SR lines in response to touch treatment should be performed.
3. Dataset S12. In the WT, CAMTA3 was phosphorylated 1 min after touching (COL_T01/COL-T0=1.814903735), while in mkk4/5, the phosphorylation level of CAMTA3 was decreased (MKK vs COL T1=0.607394125). The authors claimed that “Clear evidence for MKK4/5 phosphoregulation of CAMTA1/2/3 could however not be obtained”, it is necessary to verify whether MKK4/5 or MPK3/6 can phosphorylate CAMTA1/2/3, and whether the phenotypic defects in the camta3 mutant can be rescued (or partially rescued) in the mkk4 mkk5 camta3 triple mutant.
4. Figure 9, the authors proposed a model that incorporates Mechanosensitive ion channel proteins (MSLs) as touch sensors. Previous research found a connection between MSL10 and calcium signaling after injuring leaves (Jacob et al., Sci. Adv. 2021). However, in this paper, phosphoproteomic analysis showed that MSL5, not MSL10, was differentially phosphorylated after touch. MSL10 and MSL5 are not in the same phylogenetic subclade, and their functional similarity requires verification. It would be better if the authors discuss the role of MSL proteins more clearly in this early mechanical signalling network.
5. With regard to the term ‘early mechanical signalling’ in the title, I do believe it is appropriate to assess expression levels and phosphorylation levels as detection metrics. However, this term may be less applicable to the phenotypic changes after continuous stimulation of plants for 27 days, as the resulting phenotypes are not caused by merely “early mechanical signalling”, it better to revise the title as well as other related terms.
6. Activation of MAPKKK after mechanical stimuli should be discussed. For example, Many MAPKKKs such as MAPKKK3/5/7/9 are also rapidly phosphorylated after mechanical stimuli (Fig. 5A), is the phosphorylation related to touch-induced calcium wave, or fast auxin signaling response (Kuhn et al., Cell. 2024)?

Other concerns are:
1. Abstract: “…we report that mechanical stimulation activates a MAPKKK3/4/5-MKK4/5-MAPK3/6 cascade within 60 seconds, which leads to the induction of nearly 800 genes…”. I could not find supporting evidence for this in the manuscript.
2. The expression of touch marker genes and RNA-seq analysis are all carried out 22 min after gentle brushing. Why did the authors use this time point? Did the authors check any other time points (both shorter and longer periods after brushing) before using 22 min?
3. Fig. 1 and Fig. S1, the authors present the expression of different touch marker genes in different Arabidopsis mutants. In

order to make a proper comparison, the same set of marker genes should be used.
4. In Fig. 4B and 4C, differentially phosphorylated sites are shown, however different sets of phosphorylated proteins/peptides were selected in 4B and 4B, how did the authors select the candidates?
5. Fig. 6B-6C, compared to WT, mkk4/5 showed significant (statistical tests should be performed) decrease phosphorylation levels of MPK3/6 at 1 min after touching. However, the authors performed immunoblotting using plant materials collected 10 min and 20 min after touching! Such immunoblotting assays cannot verify the results of the phosphoproteomic data, the same time points should be used.
6. Many MAPKKKs were differentially phosphorylated within minutes after touch including MAPKKK3/4/5/7/9, Why did the authors focus on MAPKKK3/4/5 rather than MAPKKK7/9? Although the authors demonstrated that MAPKKK3/4/5 are required for thigmomorphogenesis, they cannot rule out redundant functions of MAPKKK7/9.
7. Fig. 7B, similarly, short time points (1 min after touching) should be used.

Reviewer #2

(Remarks to the Author)
Tran et al. demonstrate that the MKK4/5-MPK3/6 cascade plays a central role in touch-induced signaling (thigmomorphogenesis) in Arabidopsis. By performing RNA-seq, phosphoproteomics, and genetic analyses using mkk4/5 and mapkkk3/5/yda mutants, the authors systematically characterize how this MAPK cascade mediates mechanotransduction, clarifying previously unresolved aspects of mechanical stimulus signaling and extending prior work that had largely focused on defense and wound responses.

One of the notable aspects of this work is its multi-layered experimental design, which combines transcriptomic, phosphoproteomic, and genetic approaches to examine the role of MKK4/5 in mechanosensory signaling. The loss-of-function analyses show that MPK3/6 activation is almost completely abolished in mkk4/5 mutants (Figure 6), providing molecular evidence consistent with the observed impairment of touch responses. The attenuation of thigmomorphogenetic traits, such as leaf area reduction and bolting delay, in mkk4/5 and mapkkk3/5/yda mutants (Figure 8) supports the link between early signaling events and later morphological adaptation. Transcriptomic data further indicate that approximately 86% of MKK4/5-dependent differentially expressed genes are unchanged under untouched conditions and specifically induced after touch stimulation, suggesting that MKK4/5 primarily acts as a positive regulator of touch responses rather than by broadly altering basal states. Taken together, these findings position the MKK4/5-MPK3/6 cascade as a key component in defense-related, cell wall, and growth-associated processes during mechanosensory adaptation.

Functional validation of key phosphosites, for instance, in RBOHD, MSL5, or PIN3, could clarify whether the phosphorylation events are directly involved in touch responses. Such analysis would help to link the extensive phosphoproteomic dataset to specific functional outcomes, thereby strengthening the interpretation of how the MKK4/5-MPK3/6 cascade mediates mechanotransduction. Clarifying the functional relevance of key phosphosites would enhance the discussion.

Reviewer #3

(Remarks to the Author)
This study employs transcriptomics and phosphoproteomics to reveal the interplay between MAPKs and CAMTA transcription factors under touch induction. It demonstrates that MKK4/5 serves as a crucial regulator of touch-induced phosphorylation, while also examining phosphorylation alterations in the touch-induced jasmonic acid (JA) signaling pathway. The research has identified, for the first time, the MAPKKK3/4/5-MKK4/5-MPK3/6 cascade as a key touch- and wounding signaling pathway in plants, which regulates plant growth under stress conditions. The study exhibits significant innovation.

The research data robustly support the conclusions, with systematic data analysis and interpretation. The methodology is described in sufficient detail, though two issues require clarification:

1. The use of gentle brushing with soft brushes to simulate mechanical stimulation may have limitations. Does this approach truly replicate natural mechanical stimuli? How were the duration and intensity of leaf brushing determined?
2. Transcript-level analysis used 12-day-old plants treated for 22 minutes, whereas phosphoproteomic analysis employed 14-day-old plants treated for 25 seconds. Please explain the discrepancy in sampling timeframes.

Reviewer #4

(Remarks to the Author)
I co-reviewed this manuscript with one of the reviewers who provided the listed reports. This is part of the Nature Communications initiative to facilitate training in peer review and to provide appropriate recognition for Early Career Researchers who co-review manuscripts.

Version 1:

Reviewer comments:

Reviewer #1

(Remarks to the Author)
The authors have carefully addressed my previous concerns, and I am satisfied with their responses. The quality of the manuscript has been greatly improved. Here I have several additional minor concerns that should be addressed:

1. Did the authors compare the phosphorylation levels of MKK4/5 themselves before and after touch, given that the upstream MAPKKK regulators of MKK4/5 were also differentially phosphorylated within minutes after touching?
2. L147: “By mining the phosphoproteome data” should be “By mining the phosphoproteomic data”.
3. L160-164: “Twenty-six years ago, Ichimura et al. (2000) showed …… lead to the rapid phosphorylation of MPK3 and MPK6 (within 1 min) (Matsumura et al., 2022)”. These sentences should be moved to the last paragraph of the Introduction.
4. L281: What does it mean by “The expression pattern was more straightforward in the mkk4/5 mutant”? Do the authors intend to say “more up-regulated genes in the mkk4/5 mutant”?

Reviewer #2

(Remarks to the Author)
The authors have addressed my previous comment in a satisfactory manner.

In particular, the authors have clarified the significance of the phosphoproteomic dataset by expanding the discussion and by positioning it as a resource for future functional analyses. They also tested candidate downstream components, including RBOHD and PIN3/4/7, and showed that these factors are unlikely to play a major role in the touch responses examined here. This additional analysis improves the interpretation of the phosphoproteomic results and helps narrow the list of plausible downstream effectors.

Direct site-specific functional validation of individual phosphosites would, of course, further strengthen the mechanistic link between the phosphoproteomic dataset and touch-induced signaling outputs. However, I agree that such work is beyond the scope of the present manuscript. The current revision adequately addresses my original concern at the level expected for this study.

Reviewer #3

(Remarks to the Author)
The authors have adequately addressed all the concerns I raised in the previous round. I have no further comments.

Reviewer #4

(Remarks to the Author)
I co-reviewed this manuscript with one of the reviewers who provided the listed reports. This is part of the Nature Communications initiative to facilitate training in peer review and to provide appropriate recognition for Early Career Researchers who co-review manuscripts.

Open Access This Peer Review File is licensed under a Creative Commons Attribution 4.0 International License, which permits use, sharing, adaptation, distribution and reproduction in any medium or format, as long as you give appropriate credit to the original author(s) and the source, provide a link to the Creative Commons license, and indicate if changes were made.
In cases where reviewers are anonymous, credit should be given to 'Anonymous Referee' and the source.
The images or other third party material in this Peer Review File are included in the article's Creative Commons license, unless indicated otherwise in a credit line to the material. If material is not included in the article's Creative Commons license and your intended use is not permitted by statutory regulation or exceeds the permitted use, you will need to obtain permission directly from the copyright holder.
To view a copy of this license, visit https://creativecommons.org/licenses/by/4.0/

REVIEWER COMMENTS

Reviewer #1 (Remarks to the Author):

The MAPKKK3/4/5-MKK4/5-MAPK3/6 module has been extensively studied (Sun et al., EMBO Rep. 2018; Liu et al., J Integr Plant Biol. 2022; Wang et al., Plant Physiol. 2022; Shao et al., Mol Plant. 2020), supporting its role in regulating various aspects of plant growth and development. In this manuscript, the authors explored the functions of MAPKKK3/4/5 and MKK4/5 in thigmomorphogenesis. Using transcriptomic, phosphoproteomic and molecular approaches, the authors expanded the known functions of the well-studied MAPK module, which plays an important role in early touch response. The manuscript is generally interesting; however, key additional experiments should be provided to support the authors' conclusions.

1. In their previous paper (Darwish et al., Sci. Adv. 2022), the camta3 mutant shows strong phenotypic defects in thigmomorphogenesis and flowering time. However, mutations in the MAPKKK3/4/5-MKK4/5 module genes exhibit much weaker phenotypic defects. Since the Arabidopsis genome has 20 MAPKs, 10 MAPKKs and 80 MAPKKKs (Kumar et al., J. Plant Biochem. Biotechnol. 2020), redundant functions likely exist.

Thank you for this constructive comment, we have now discussed this potential redundancy. We also carried qPCR out on other MAPKKK mutants, including *m3kδ1/δ5-1/δ6-1/δ7* and *m3kδ1/δ5-2/δ6-1/δ7*, to check if MAPKKK δ1, 5, 6 and 7 play roles in response to touch (Fig. S8A). *TCH2* was significantly less induced in *m3kδ1/δ5-2/δ6-1/δ7* but there were no significant changes in other touch-responsive genes between the mutants and WT, suggesting that MAPKKK δ1, 5, 6 and 7 are unlikely to be major players in plant touch response.

The different thigmomorphogenesis phenotypes between *camta3* plants and the lines with altered expression of the components of the MPKKK-MKK signalling cascade could be a consequence of only partially overlapping expression of touch-induced genes. While we observed a large overlap in the transcriptomic profile of touched *camta1/2/3* and *mkk4/5* plants, both genotypes also showed a unique set of exclusively regulated genes (Fig. 3). These sets of genes may contribute differently to the final phenotypes.

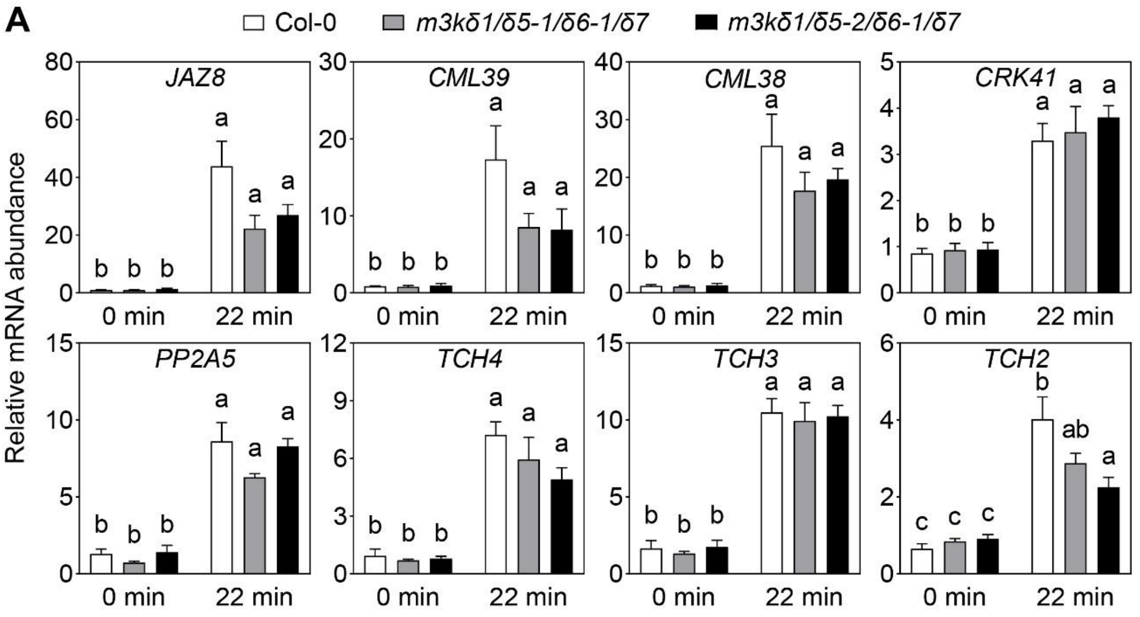

Figure S8A. Analysis of touch marker gene expression in the m3kδ1/δ5-1/δ6-1/δ7 and m3kδ1/δ5-2/δ6-1/δ7 mutants*. 12-day-old seedlings before (0 min) and 22 min after touching by gentle brushing were collected for qRT-PCR. The expression of selected touch marker genes was measured in Col-0 (WT) versus the m3kδ1/δ5-1/δ6-1/δ7 and m3kδ1/δ5-2/δ6-1/δ7 mutants. The y axis represents the relative mRNA level (n=5, ±SE). Statistical significance was based on Kruskal-Wallis test followed by Wilcoxon signed-rank tests. Different letters represent the significant differences between genotypes ($p < 0.05$).*

2. In order to demonstrate the role of MAPK3/6 in thigmomorphogenesis, phenotypic analysis of the mpk6-3 mutant and MPK6SR lines in response to touch treatment should be performed.

We have now performed thigmomorphogenesis analysis in and *mpk6-3* and two alleles of the MPK6SR lines. However, we could not detect significant differences with WT (Fig S9). It should be noted that it was not feasible to apply Na-PP1 to the MPK6SR plants during their entire lifecycle due to severe developmental effects, so the inhibition of MPK3/6 function would not have been complete.

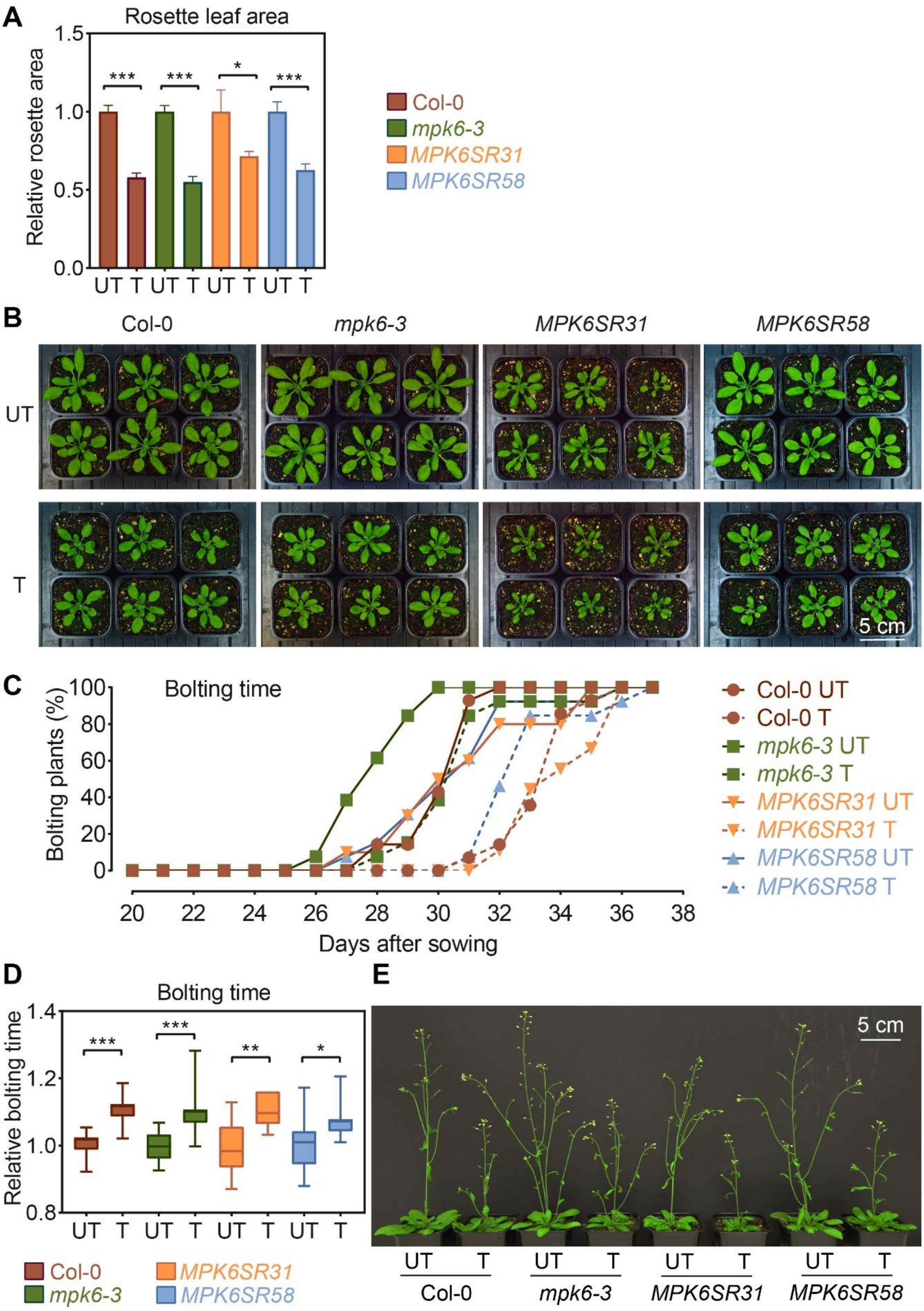

Figure S9. Analysis of the mpk6-3 mutant and MPK6SR lines on thigmomorphogenesis. A. *Relative rosette leaf area of 28-day-old plants, either untouched or after 14 days of regular touching (n=9-14, ±SE) (*=p<0.05, ***=p<0.001, ****=p<0.0001).* ***B.*** *A representative picture of 28-day-old untouched plants or plants in which touch treatment had been applied twice-daily for 14 days. Scale bar = 5 cm.*

***C.** Line graph showing the percentage of bolting plants over the growth period (days after sowing). D. Box and whisker plot shows relative bolting time under untouched and touched conditions (n=9-14, ±SE) (*=p<0.05, ***=p<0.001, ****=p<0.0001). E. A representative picture of 38-day-old untouched plants or plants in which touch treatment had been applied twice-daily for 24 days. Scale bar = 5 cm. UT=untouched, T=touched.*

3. Dataset S12. In the WT, CAMTA3 was phosphorylated 1 min after touching (COL_T01/COL-T0=1.814903735), while in mkk4/5, the phosphorylation level of CAMTA3 was decreased (MKK vs COL T1=0.607394125). The authors claimed that "Clear evidence for MKK4/5 phosphoregulation of CAMTA1/2/3 could however not be obtained", it is necessary to verify whether MKK4/5 or MPK3/6 can phosphorylate CAMTA1/2/3, and whether the phenotypic defects in the camta3 mutant can be rescued (or partially rescued) in the mkk4 mkk5 camta3 triple mutant.

This was well observed. CAMTA3 S964 is indeed phosphorylated 1.814x in Col-0 at 1 min. And this is significant as a Time effect (p-value 0.01). However, there is no Genotype or Genotype x Time (Interaction) effect, so according to the statistics there is no evidence that MKK4/5 affect the phosphorylation of that site. A previous study reported several potential MAPK-phosphorylation sites on CAMTA3 (https://academic.oup.com/plphys/article/184/2/1056/6117871), however the S964 site is not likely a MAPK target as it lacks the SP motif (the sequence in that region is RVK**S**MVQYPE). S964 phosphorylation was also not affected in the *mkk4/5* mutants. The S587 is a potential MAPK phosphorylation site, but the touch-dependent effect on its phosphorylation is very limited (0.7x after 3 minutes). The above study also showed that mutation of S587 does not show significant effects on MPK3/6-dependent phosphorylation, even in combination with 4 other MPK3/6 phosphorylation site mutations, indicating a very high degree of redundancy between MPK3/6 phosphorylation sites on CAMTA3, and no obvious link with touch-dependent phosphorylation. We have broadened the discussion (including this citation) to further clarify why we hypothesized that phosphorylation of CAMTA3 by MKK4/5 or MPK3/6 in response to touch is unlikely.

Regarding creation of mutants, both *mkk45* and *camta123* have a comparable gene expression phenotype. Therefore, creating a triple mutant will likely not rescue the *camta3* phenotype (it may make it even stronger). Also, we would possibly need to make a quintuple mutant with *camta123*, as there is redundancy (*camta3* single mutants have a much weaker transcriptional phenotype as reported by Darwish et al., 2022, Sci Adv).

4. Figure 9, the authors proposed a model that incorporates Mechanosensitive ion channel proteins (MSLs) as touch sensors. Previous research found a connection between MSL10 and calcium signaling after injuring leaves (Jacob et al., Sci. Adv. 2021). However, in this paper, phosphoproteomic analysis showed that MSL5, not MSL10, was differentially phosphorylated after touch. MSL10 and MSL5 are not in the same phylogenetic subclade, and their functional similarity requires verification. It would be better if the authors discuss the role of MSL proteins more clearly in this early mechanical signalling network.

Thank you for highlighting the potential role of MSL proteins in touch signalling. We previously showed that even quintuple *msl4/5/6/9/10* mutants do not show a clear effect on touch-induced gene expression (Van Moerkercke et al., 2019, PNAS). A recent study showed that MSLs may be involved in cell-to-cell spreading of calcium signals after mechanical stimulation, rather than acting

in local touch perception (https://www.nature.com/articles/s41467-025-61770-6.pdf). This has now been discussed in the Introduction.

5. With regard to the term 'early mechanical signalling' in the title, I do believe it is appropriate to assess expression levels and phosphorylation levels as detection metrics. However, this term may be less applicable to the phenotypic changes after continuous stimulation of plants for 27 days, as the resulting phenotypes are not caused by merely "early mechanical signalling", it better to revise the title as well as other related terms.

We understand your concern. With 'early' in the title, we wanted to emphasize strong effects of MAPK mutants in the phosphorylation and transcriptomics data within minutes after touching. We agree that the thigmomorphogenesis data also suggests a longer-term effect. We have removed 'early' from the title.

6. Activation of MAPKKK after mechanical stimuli should be discussed. For example, Many MAPKKKs such as MAPKKK3/5/7/9 are also rapidly phosphorylated after mechanical stimuli (Fig. 5A), is the phosphorylation related to touch-induced calcium wave, or fast auxin signaling response (Kuhn et al., Cell. 2024)?

Thank you for this suggestion, we have now further elaborated on this aspect in the discussion.

Other concerns are:
1. Abstract: "…we report that mechanical stimulation activates a MAPKKK3/4/5-MKK4/5-MAPK3/6 cascade within 60 seconds, which leads to the induction of nearly 800 genes…". I could not find supporting evidence for this in the manuscript. Mutating MAPKKK3/4/5 blocks this MAPK3/6 activation.

We supported this statement based on the observation that we see significantly increased phosphorylation of e.g. MAPKKK5, MKK5 and MAPK3/6 already within one minute (see Table S10, as well as figure 7B in the revised manuscript). In addition, RNA-seq analysis of the *mkk4/5* mutant shows that 779 genes are significantly less induced 22 minutes after touching as compared to in the WT. Therefore, we believe this claim is supported by the data.

2. The expression of touch marker genes and RNA-seq analysis are all carried out 22 min after gentle brushing. Why did the authors use this time point? Did the authors check any other time points (both shorter and longer periods after brushing) before using 22 min?

Previous reports have indicated that the peak in gene expression for many genes is approximately 22-25 minutes after mechanical treatment (e.g. Van Moerkercke et al., 2019, PNAS; Darwish et al., 2022, Sci Adv.). As we agree that this is an important point, we have now conducted an additional time course experiment including time points before and after 22 minutes in *camta123* and *mkk4/5* mutants (Figure S4). The results confirm that in general the peak in gene expression sits in between 10 and 30 minutes after touching.

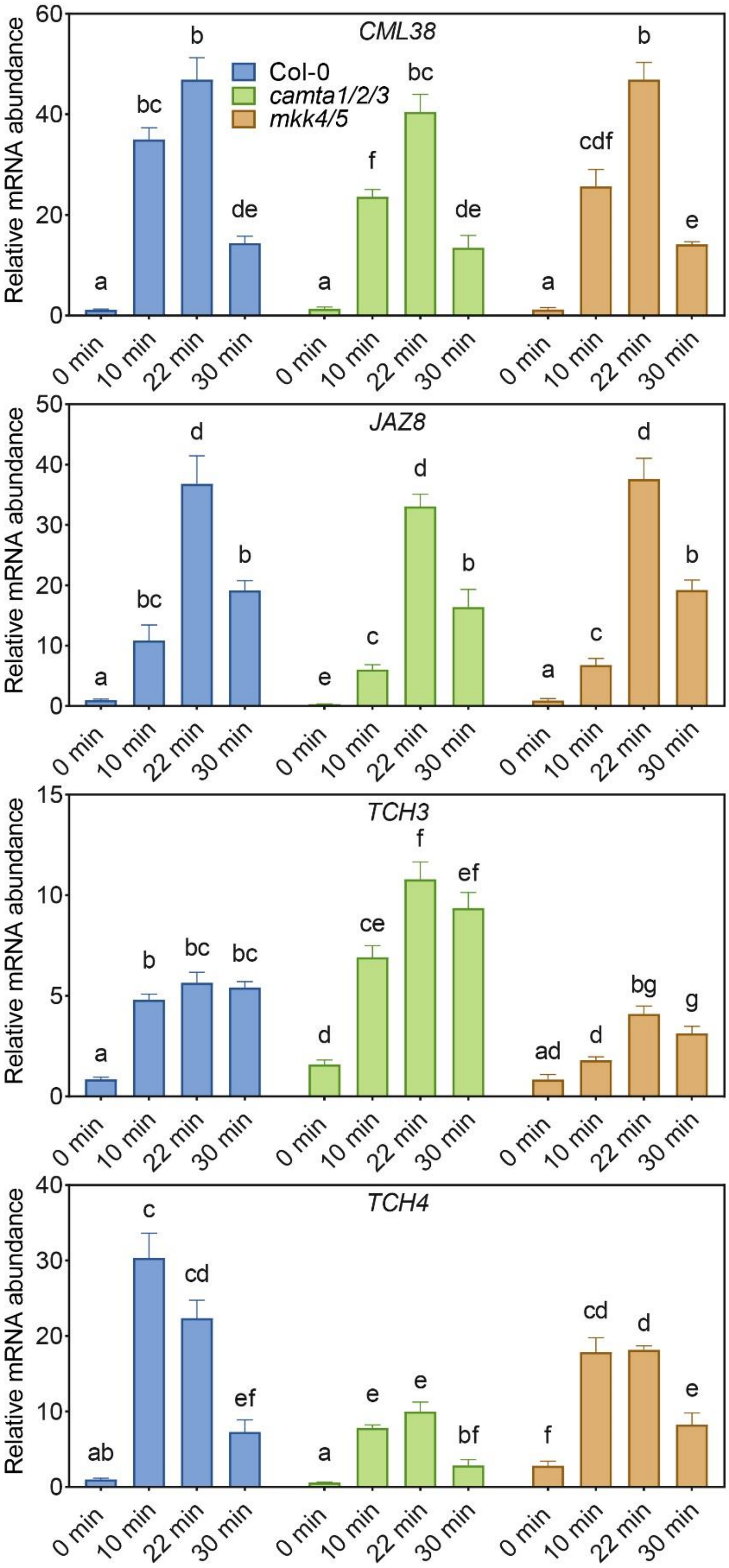

Figure S4. Analysis of touch marker gene expression at different time points in the mutants camta1/2/3 and mkk4/5*. 12-day-old seedlings before (0 min) and 10, 22 and 30 min after touching by gentle brushing were collected for qRT-PCR. The expression of selected touch marker genes was measured in Col-0 (WT) versus camta1/2/3 and mkk4/5. The y axis represents the relative mRNA*

level (n=4, ±SE). Statistical significance was based on Kruskal-Wallis test followed by Wilcoxon signed-rank tests. Different letters represent the significant differences between genotypes ($p < 0.05$).

3. Fig. 1 and Fig. S1, the authors present the expression of different touch marker genes in different Arabidopsis mutants. In order to make a proper comparison, the same set of marker genes should be used.

We agree that it is useful to have a consistent set of marker genes analysed in the different mutants. Therefore, we have now performed additional qRT-PCR measurements to have the same marker genes throughout the manuscript. The new data was added to Figure 1 and S1-3.

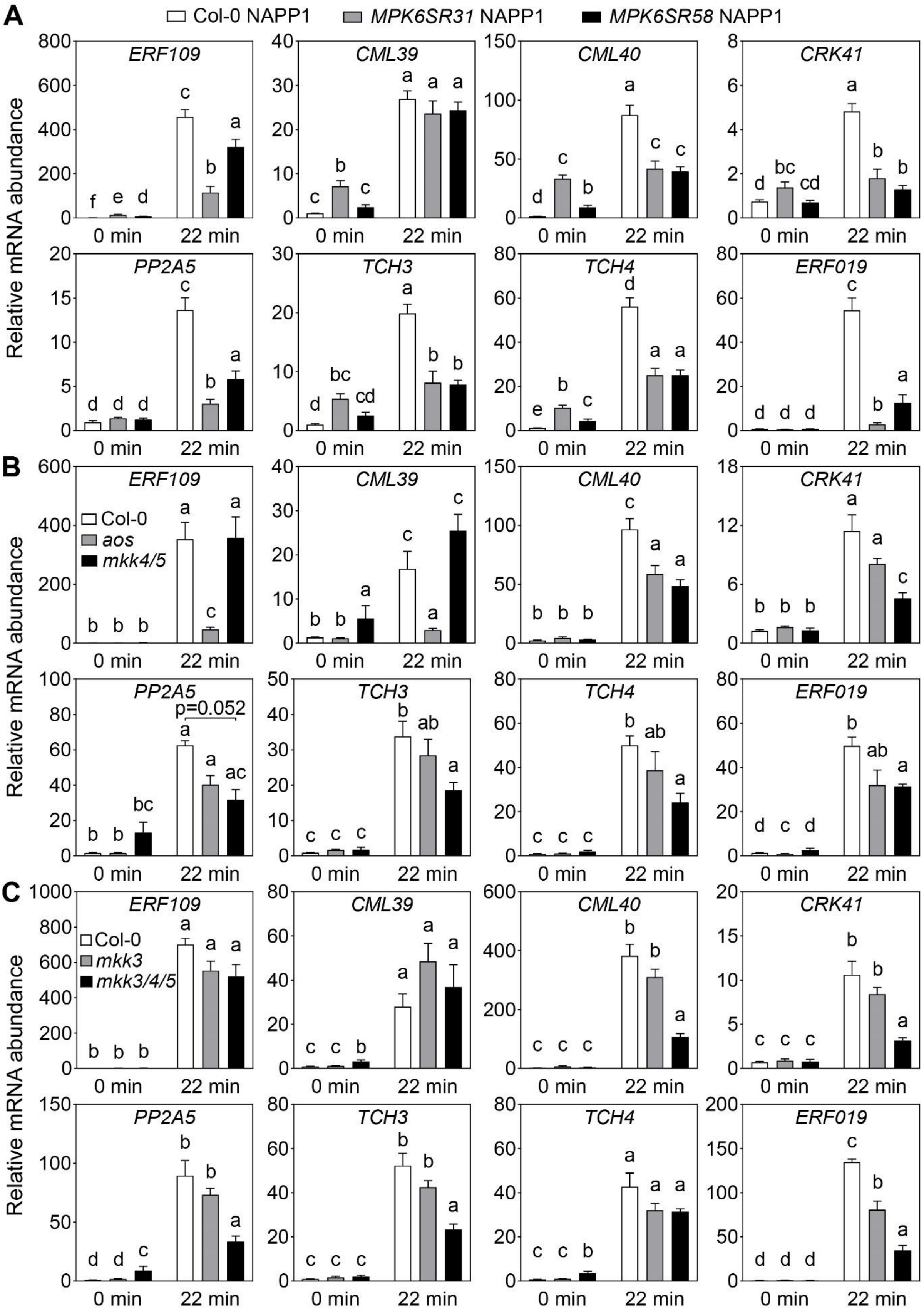

Figure 1. Analysis of touch marker gene expression in Arabidopsis mutants. *12-day-old seedlings before (0 min) and 22 min after touching by gentle brushing were collected for qRT-PCR. The expression of selected touch marker genes was measured in Col-0 (WT) treated with NAPP1 versus*

MPK6SR31 and MPK6SR58 treated with NAPP1 ***(A)****, WT versus aos and mkk4/5* ***(B)****, WT versus mkk3 and mkk3/4/5* ***(C)****. The y axis represents the relative mRNA level (n=6 (A), n=6 (B), n=5 (C), ±SE). Statistical significance was based on Kruskal-Wallis test followed by Wilcoxon signed-rank tests. Different letters represent the significant differences between genotypes (p < 0.05).*

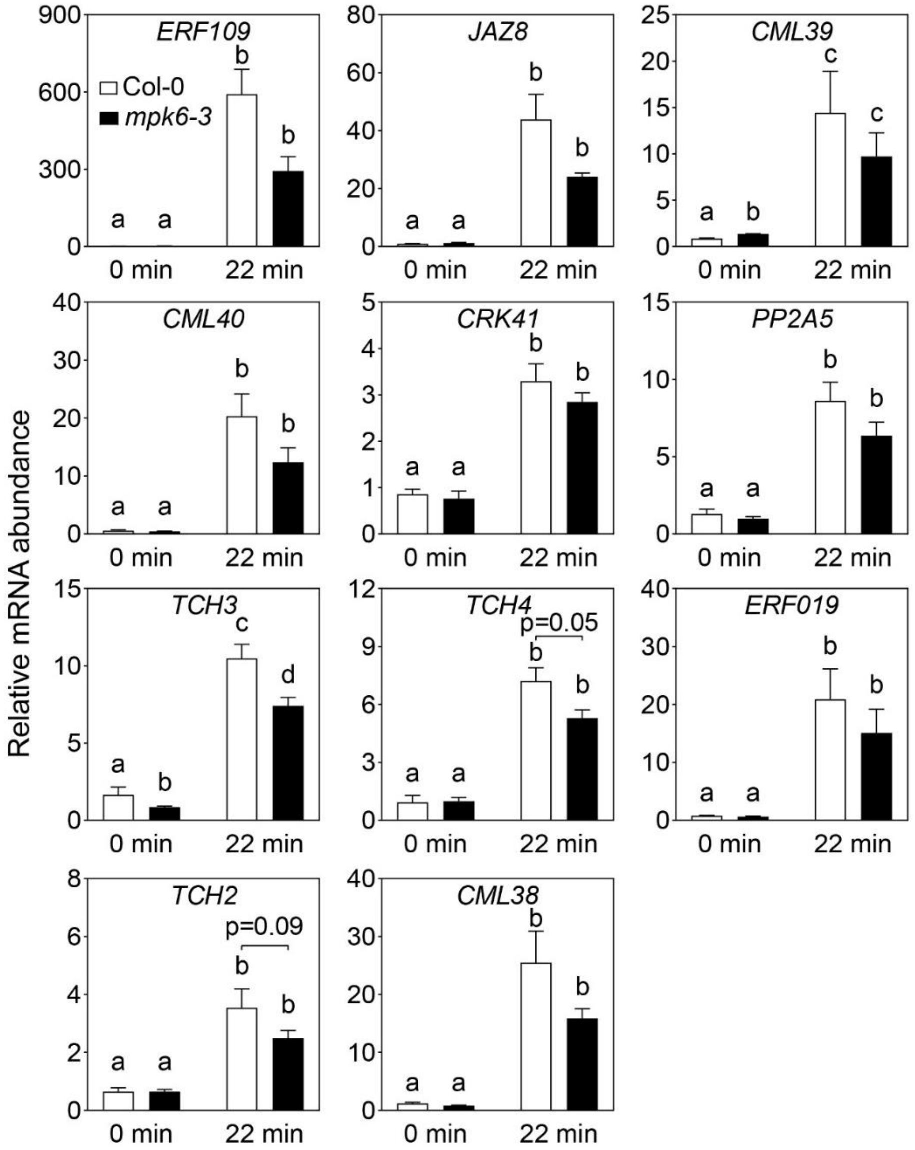

Figure S1. Analysis of touch marker gene expression in the mpk6-3 mutant. 12-day-old seedlings before (0 min) and 22 min after touching by gentle brushing were collected for qRT-PCR. *The expression of selected touch marker genes was measured in Col-0 (WT) versus the mpk6-3 mutant. The y axis represents the relative mRNA level (n=5, ±SE). Statistical significance was based on Kruskal-Wallis test followed by Wilcoxon signed-rank tests. Different letters represent the significant differences between genotypes (p < 0.05).*

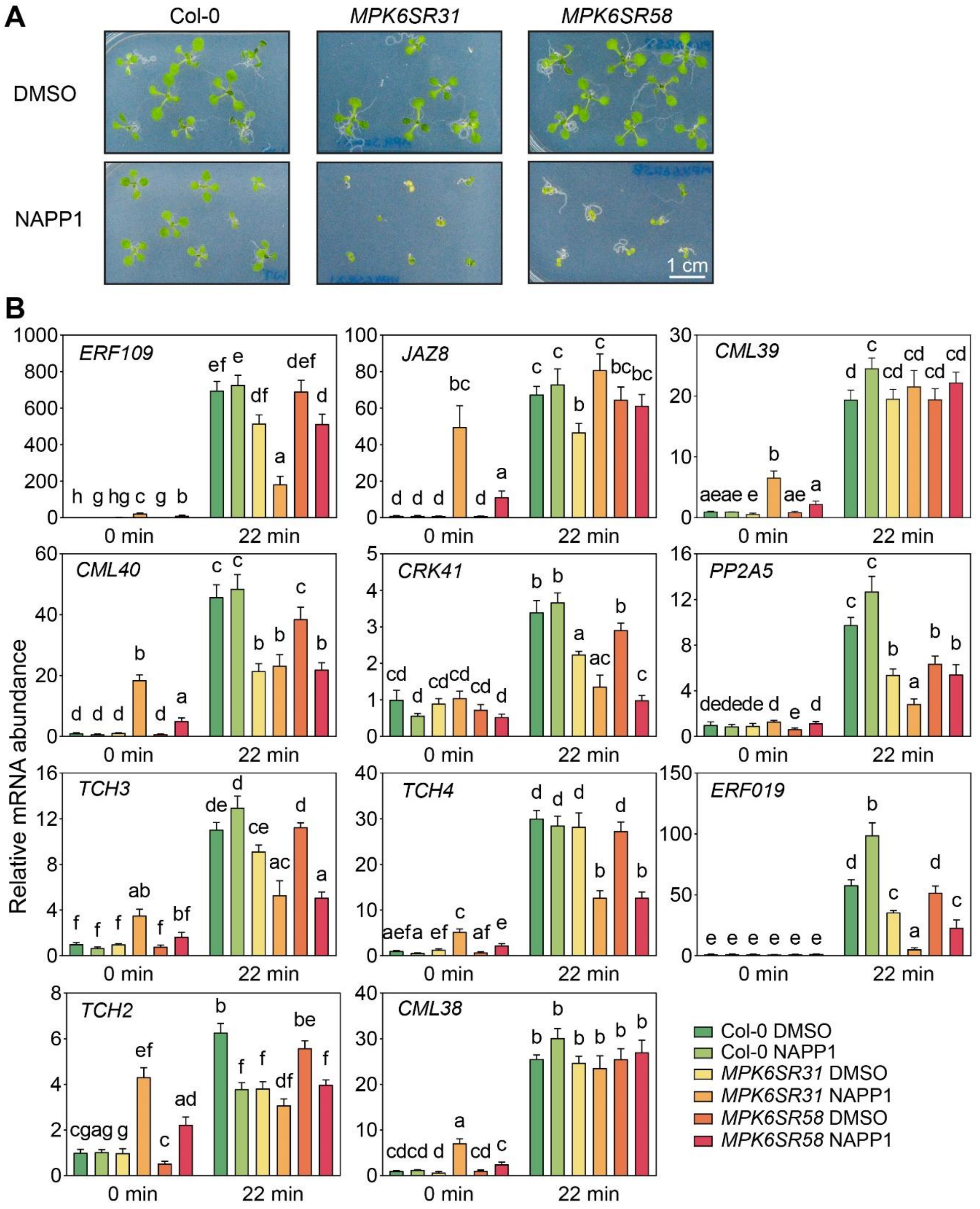

Figure S2. Analysis of touch marker gene expression in the MPK6SR lines. A. *A representative picture of 10-day-old WT and MPK6SR lines seedlings grown on half-strength MS containing either DMSO (mock) or 1 µM NAPP1. Scale bar = 1 cm.* ***B.*** *Touch marker gene expression in the MPK6SR lines. 12-day-old seedlings before (0 min) and 22 min after touching by gentle brushing were collected for qRT-PCR. The expression of selected touch marker genes was measured in Col-0 (WT) versus the MPK6SR lines (MPK6SR31 and MPK6SR58). The y axis represents the relative mRNA level (n=6, ±SE). Statistical significance was based on Kruskal-Wallis test followed by Wilcoxon signed-rank tests. Different letters represent the significant differences between genotypes (p < 0.05).*

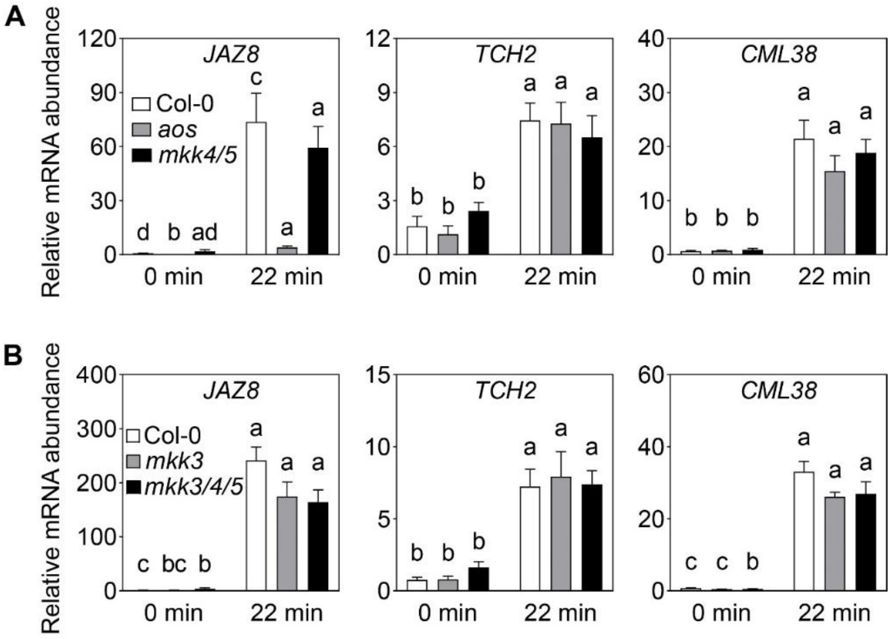

Figure S3. Analysis of touch marker gene expression in the mutants aos, mkk4/5, mkk3 and mkk3/4/5. 12-day-old seedlings before (0 min) and 22 min after touching by gentle brushing were collected for qRT-PCR. *The expression of selected touch marker genes was measured in Col-0 (WT) versus the aos and mkk4/5 mutants* ***(A)****, and WT versus the mkk3 and mkk3/4/5 mutants* ***(B)****. The y axis represents the relative mRNA level (n=5, ±SE). Statistical significance was based on Kruskal-Wallis test followed by Wilcoxon signed-rank tests. Different letters represent the significant differences between genotypes (p < 0.05).*

4. In Fig. 4B and 4C, differentially phosphorylated sites are shown, however different sets of phosphorylated proteins/peptides were selected in 4B and 4B, how did the authors select the candidates?

These are a selection of genotype-dependent phosphosites for either *mkk4/5* or *aos* (Genotype effect), so they are different sets for each individual mutant. The full lists are available in Table S11.

5. Fig. 6B-6C, compared to WT, mkk4/5 showed significant (statistical tests should be performed) decrease phosphorylation levels of MPK3/6 at 1 min after touching. However, the authors performed immunoblotting using plant materials collected 10 min and 20 min after touching! Such immunoblotting assays cannot verify the results of the phosphoproteomic data, the same time points should be used.

Figure 6B represents the non-imputed phosphoproteomics data, as it represents the dynamic range better. However, the statistical analyses were performed on the imputed data (Table S10), where statistical differences between Col-0 and *mkk4/5* were observed for MPK3/6 phosphorylation.

We had performed the Western blots on samples taken 10 and 20 minutes after touching, not only to confirm that MPK3/6 are less phosphorylated within the time span of the phosphoproteomics course (using 10 minutes as an example), but also to give an overview of what happens after the time points covered in the phosphoproteomics data set (20 minutes). We agree that it would be useful to confirm the phosphoproteomics data in a stricter sense, so we have set up new

experiments and performed WBs on plants 1, 3 and 10 minutes after brushing. This data has been added to Figure 6C and shows that MAPK3/6 phosphorylation can already be observed after 1 minute, and peaks after 3 minutes, in line with the phosphoproteomics data. Also, the much weaker MPK3/6 phosphorylation at these very early time points in *mkk4/5* mutants was confirmed.

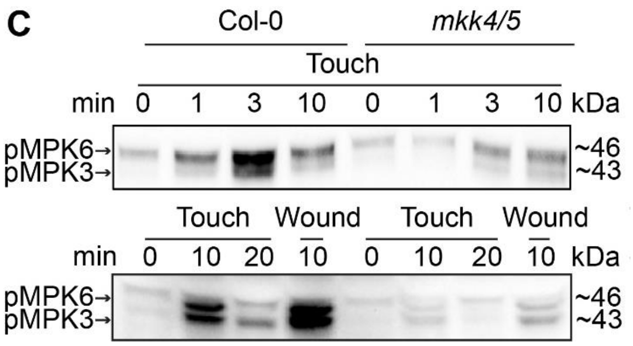

Figure 6C. Immunoblots using an antibody against phosphorylated MAPKs in WT and mkk4/5 before and after touch or wounding treatment.

6. Many MAPKKKs were differentially phosphorylated within minutes after touch including MAPKKK3/4/5/7/9, Why did the authors focus on MAPKKK3/4/5 rather than MAPKKK7/9? Although the authors demonstrated that MAPKKK3/4/5 are required for thigmomorphogenesis, they cannot rule out redundant functions of MAPKKK7/9.

Indeed, given the large number MAPKKKs in Arabidopsis, there is likely to be further redundancy, though our data shows that MAPKKK3/4/5 have a major contribution to MAPK3/6 phosphorylation and downstream gene expression (see e.g. Fig 7A-B). We focused on MAPKKK3/4/5 as there was prior evidence that they may regulate MKK4/5 phosphorylation during other stresses. MKKK9 (MEKK2) on the other hand is a repressor of MAPK4 phosphorylation by directly binding MAPK4 and inhibiting its phosphorylation by upstream MKKs. (https://onlinelibrary.wiley.com/doi/10.1111/tpj.14763)

MKKK7 was found to be redundant with MKKK6 (also known as MKKKepsilon1/2) operating in pollen development. However, the double mutant is lethal, so we did not pursue this further (https://onlinelibrary.wiley.com/doi/10.1111/j.1365-313X.2006.02863.x).

In addition (as described above), we have now analysed quadruple mutants in MAPKKK δ1, 5, 6 and 7. However, we did not find any consistent defects in touch-induced gene expression responses, suggesting that they are not major players (Fig S8A).

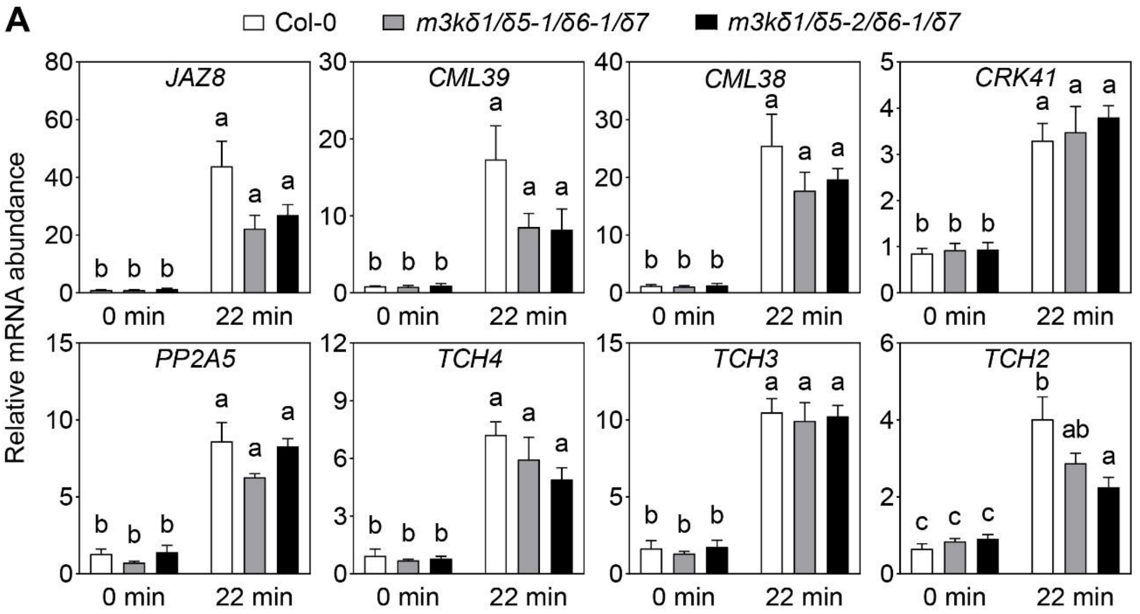

__Figure S8A. Analysis of touch marker gene expression in the m3kδ1/δ5-1/δ6-1/δ7 and m3kδ1/δ5-2/δ6-1/δ7 mutants__. 12-day-old seedlings before (0 min) and 22 min after touching by gentle brushing were collected for qRT-PCR. The expression of selected touch marker genes was measured in Col-0 (WT) versus the m3kδ1/δ5-1/δ6-1/δ7 and m3kδ1/δ5-2/δ6-1/δ7 mutants. The y axis represents the relative mRNA level (n=5, ±SE). Statistical significance was based on Kruskal-Wallis test followed by Wilcoxon signed-rank tests. Different letters represent the significant differences between genotypes (p < 0.05).

7. Fig. 7B, similarly, short time points (1 min after touching) should be used.

We have now also set up new experiments and performed WBs on *mapkkk3/5* and *mapkkk3/4/5* mutants 1, 3 and 10 minutes after brushing. This data has been added to Figure 7B.

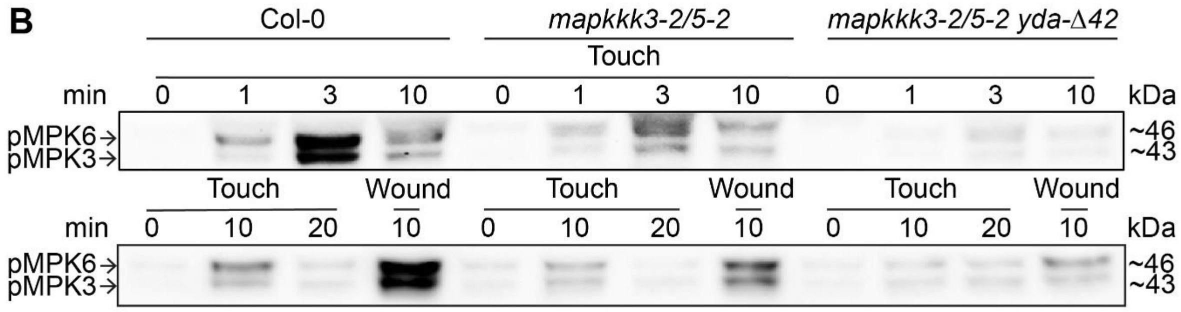

Figure 7B. Immunoblots using an antibody against phosphorylated MAPKs on WT, mapkkk3-2/5-2 and mapkkk3-2/5-2 yda-Δ42 mutants before and after touch or wounding treatment.

Reviewer #2 (Remarks to the Author):

Tran et al. demonstrate that the MKK4/5-MPK3/6 cascade plays a central role in touch-induced signaling (thigmomorphogenesis) in Arabidopsis. By performing RNA-seq, phosphoproteomics, and genetic analyses using mkk4/5 and mapkkk3/5/yda mutants, the authors systematically characterize

how this MAPK cascade mediates mechanotransduction, clarifying previously unresolved aspects of mechanical stimulus signaling and extending prior work that had largely focused on defense and wound responses.

One of the notable aspects of this work is its multi-layered experimental design, which combines transcriptomic, phosphoproteomic, and genetic approaches to examine the role of MKK4/5 in mechanosensory signaling. The loss-of-function analyses show that MPK3/6 activation is almost completely abolished in mkk4/5 mutants (Figure 6), providing molecular evidence consistent with the observed impairment of touch responses. The attenuation of thigmomorphogenetic traits, such as leaf area reduction and bolting delay, in mkk4/5 and mapkkk3/5/yda mutants (Figure 8) supports the link between early signaling events and later morphological adaptation. Transcriptomic data further indicate that approximately 86% of MKK4/5-dependent differentially expressed genes are unchanged under untouched conditions and specifically induced after touch stimulation, suggesting that MKK4/5 primarily acts as a positive regulator of touch responses rather than by broadly altering basal states. Taken together, these findings position the MKK4/5-MPK3/6 cascade as a key component in defense-related, cell wall, and growth-associated processes during mechanosensory adaptation.

Functional validation of key phosphosites, for instance, in RBOHD, MSL5, or PIN3, could clarify whether the phosphorylation events are directly involved in touch responses. Such analysis would help to link the extensive phosphoproteomic dataset to specific functional outcomes, thereby strengthening the interpretation of how the MKK4/5-MPK3/6 cascade mediates mechanotransduction. Clarifying the functional relevance of key phosphosites would enhance the discussion.

We thank the reviewer for the positive evaluation of our work. We agree that the phosphoproteome data will provide a useful starting point for further exploration of important downstream touch-phosphorylated sites. However, finding such key phosphosites is a major undertaking in itself, given the hundreds of proteins that are phosphorylated by touching within minutes. Then subsequent functional validation of such sites (e.g. by complementing mutants with phospho-mimic or phospho-dead variants) is highly complex and time consuming, with often multiple phosphosites on the same protein. We have added a section in the discussion highlighting that the phosphoproteome data set will be a resource for future functional analysis.

As we are also excited to find such downstream key phosphosites, we have explored the roles of RBOHD and PIN3 during touch responses (our previous work showed that even quintuple mutants in MSL4/5/6/9/10 showed no observable defects in touch signalling; Van Moerkercke et al., 2019, PNAS). Therefore, we obtained *rbohd*¸ *pin3* and even *pin3/4/7* (our data set showed that also PIN4 and PIN7 show MKK4/5 and touch-dependent phosphorylation changes) mutants. We have tested gene expression of several markers 22 minutes following touch-treatment on these mutants, but we did not observe major changes compared to the WT (Fig S6), indicating RBOHD and PIN3/4/7 are unlikely to be important players in touch responses. We believe that further identification and functional characterisation of additional key downstream phosphosites based on the phosphoproteome data is indeed highly interesting, but beyond the scope of this manuscript.

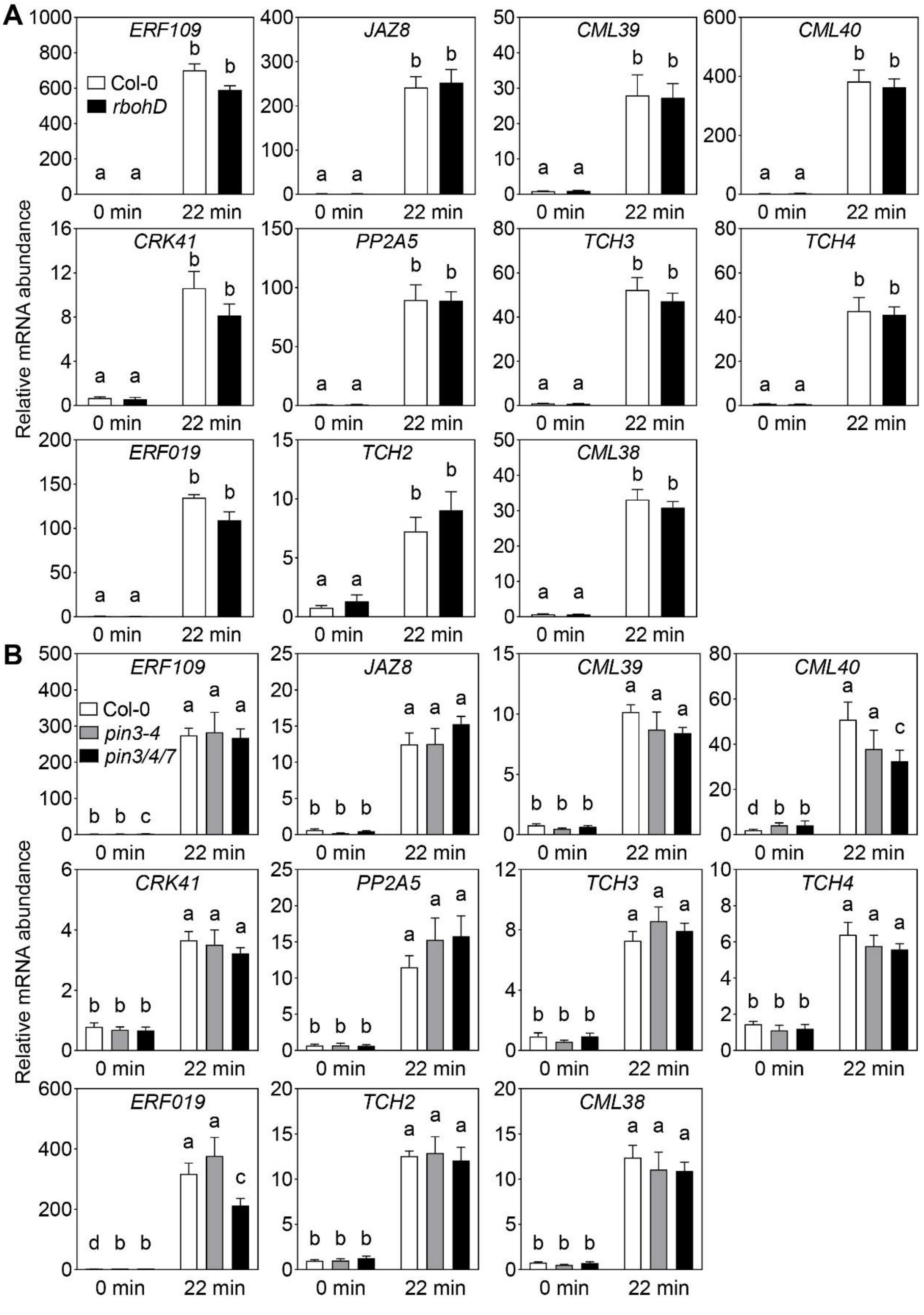

Figure S6. Analysis of touch marker gene expression in the rbohD, pin3-4 and pin3/4/7 mutants.
12-day-old seedlings before (0 min) and 22 min after touching by gentle brushing were collected for qRT-PCR. The expression of selected touch marker genes was measured in Col-0 (WT) versus the mutants rbohD ***(A)****, pin3-4 and pin3/4/7* ***(B)****. The y axis represents the relative mRNA level (n=6, ±SE). Statistical significance was based on Kruskal-Wallis test followed by Wilcoxon signed-rank tests. Different letters represent the significant differences between genotypes (p < 0.05).*

Reviewer #3 (Remarks to the Author):

This study employs transcriptomics and phosphoproteomics to reveal the interplay between MAPKs and CAMTA transcription factors under touch induction. It demonstrates that MKK4/5 serves as a crucial regulator of touch-induced phosphorylation, while also examining phosphorylation alterations in the touch-induced jasmonic acid (JA) signaling pathway. The research has identified, for the first time, the MAPKKK3/4/5-MKK4/5-MPK3/6 cascade as a key touch- and wounding signaling pathway in plants, which regulates plant growth under stress conditions. The study exhibits significant innovation.

We thank the reviewer for the positive evaluation of our work.

The research data robustly support the conclusions, with systematic data analysis and interpretation. The methodology is described in sufficient detail, though two issues require clarification:

1. The use of gentle brushing with soft brushes to simulate mechanical stimulation may have limitations. Does this approach truly replicate natural mechanical stimuli? How were the duration and intensity of leaf brushing determined?

We have measured the approximate pressure exerted by brushing to be around 2 g/cm^2, which is in the same order as previous reports by us and other groups (Darwish et al., 2022; Wang et al., 2018). Around 10 brushings were exerted per plant, taking about 30-45 seconds. This information is now included in the methods section. Whether this truly replicates natural mechanical stimuli is hard to assess, as there would be a very wide range of stimulations from various sources, some being stronger (like heavy winds or rain, larger animals) and others likely weaker (like insects crawling). The brushing therefore represents a moderate mechanical stimulus, without inflicting severe physical damage.

2. Transcript-level analysis used 12-day-old plants treated for 22 minutes, whereas phosphoproteomic analysis employed 14-day-old plants treated for 25 seconds. Please explain the discrepancy in sampling timeframes.

For the transcript analysis, we collect after 22 minutes as that is the peak in gene expression for many marker genes (see also Figure S4). However, the treatment duration itself was approximately the same at 25 s for the phosphoproteomics experiment, and 30-45s for the transcriptome analyses. For the phosphoproteomics experiment, a slightly older set of plants was used (14 days) as the phosphoproteomic study requires as a lot of fresh weight material to do the extractions, phospho-enrichment and proteomics.

Reviewer #4 (Remarks to the Author):

I co-reviewed this manuscript with one of the reviewers who provided the listed reports. This is part of the Nature Communications initiative to facilitate training in peer review and to provide appropriate recognition for Early Career Researchers who co-review manuscripts.

Thank you for taking the time to evaluate our work.

We thank the reviewers for their efforts to evaluate our revision. We have marked the changes in the revised manuscript in yellow, and provided a point-by-point response to all Reviewer comments. Note that smaller changes to formatting etc. requested by the Editorial office are not marked in the manuscript to reduce complexity.

REVIEWERS' COMMENTS

Reviewer #1 (Remarks to the Author):

The authors have carefully addressed my previous concerns, and I am satisfied with their responses. The quality of the manuscript has been greatly improved. Here I have several additional minor concerns that should be addressed:

1. Did the authors compare the phosphorylation levels of MKK4/5 themselves before and after touch, given that the upstream MAPKKK regulators of MKK4/5 were also differentially phosphorylated within minutes after touching?

Thank you for this suggestion. In our phosphoproteomic data, only MKK5 was detected. There is a time effect on MKK5 Serine 12. This site is differentially more phosphorylated after touching. We added the following line to the discussion *'In our phosphoproteomic data, MKK5 itself was also significantly more phosphorylated within one minute after touching on serine S12 (MKK4 phosphopeptides could not be detected).'*

2. L147: "By mining the phosphoproteome data" should be "By mining the phosphoproteomic data".

Thank you for this suggestion. We have rephrased the sentence as suggested.

3. L160-164: "Twenty-six years ago, Ichimura et al. (2000) showed ...... lead to the rapid phosphorylation of MPK3 and MPK6 (within 1 min) (Matsumura et al., 2022)". These sentences should be moved to the last paragraph of the Introduction.

Thank you for this suggestion. We have moved the sentence to the last paragraph of the introduction.

4. L281: What does it mean by "The expression pattern was more straightforward in the mkk4/5 mutant"? Do the authors intend to say "more up-regulated genes in the mkk4/5 mutant"?

We compared the touch-responsive DEGs pattern between *camta1/2/3* and *mkk4/5* mutants. *camta1/2/3* showed 988 DEGs, of which 596 genes (~60%) were positively regulated, 30 genes (~3%) were negatively regulated, and 362 genes (~37%) were attenuated by CAMTA1/2/3. *mkk4/5* showed 905 DEGs, of which 779 genes (~86%) were positively regulated, 86 genes (~10%) were negatively regulated, and only 40 genes (~4%) were attenuated by MKK4/5. What we mean is that there is a more linear type of response in the *mkk4/5*, with most genes being positively regulated by MKK4/5. The patterns in *camta123* were a bit more complicated, as many genes were 'attenuated' which means that they seem to be even more induced by lack of CAMTA123, while others showed a more classical lower induction in the absence of CAMTA123. This explains why "the expression pattern was more straightforward in the mkk4/5 mutant".

The manuscript currently reads: "*The expression pattern was more straightforward in the mkk4/5 mutant as compared to the camta1/2/3 mutant, as the vast majority of DEGs (779 genes, ~86%) required MKK4/5 for their full induction, and 86 (~10%) genes required MKK4/5 for their full downregulation (Fig. 2dD) and (Dataset Supplementary Data 4Bb). Only 4% (40 genes) were attenuated by MKK4/5, meaning they were more induced or more downregulated after touch in mkk4/5 as compared to the WT (Fig. 2D2d) and (Dataset Supplementary Data 4bB).*" We hope that this clarifies the term 'straightforward'

Reviewer #2 (Remarks to the Author):

The authors have addressed my previous comment in a satisfactory manner.

In particular, the authors have clarified the significance of the phosphoproteomic dataset by expanding the discussion and by positioning it as a resource for future functional analyses. They also tested candidate downstream components, including RBOHD and PIN3/4/7, and showed that these factors are unlikely to play a major role in the touch responses examined here. This additional analysis improves the interpretation of the phosphoproteomic results and helps narrow the list of plausible downstream effectors.

Direct site-specific functional validation of individual phosphosites would, of course, further strengthen the mechanistic link between the phosphoproteomic dataset and touch-induced signaling outputs. However, I agree that such work is beyond the scope of the present manuscript. The current revision adequately addresses my original concern at the level expected for this study.

We thank the reviewer for the positive evaluation of our revision.

Reviewer #3 (Remarks to the Author):

The authors have adequately addressed all the concerns I raised in the previous round. I have no further comments.

We thank the reviewer for the positive evaluation of our revision.

Reviewer #4 (Remarks to the Author):

I co-reviewed this manuscript with one of the reviewers who provided the listed reports. This is part of the Nature Communications initiative to facilitate training in peer review and to provide appropriate recognition for Early Career Researchers who co-review manuscripts.

We thank the reviewer for the positive evaluation of our revision.
